# Synthesis of Hydrazone Derivatives of 4-[4-Formyl-3-(2-oxochromen-3-yl)pyrazol-1-yl]benzoic acid as Potent Growth Inhibitors of Antibiotic-resistant *Staphylococcus aureus* and *Acinetobacter baumannii*

**DOI:** 10.3390/molecules24112051

**Published:** 2019-05-29

**Authors:** Jedidiah Whitt, Cameron Duke, Anthony Sumlin, Steven A. Chambers, Rawan Alnufaie, David Gilmore, Todd Fite, Alexei G. Basnakian, Mohammad A. Alam

**Affiliations:** 1 Department of Chemistry and Physics, College of Science and Mathematics, Arkansas State University, Jonesboro, AR 72467, USA; jedidiah.whitt@smail.astate.edu (J.W.); cameron.duke@smail.astate.edu (C.D.); anthony.sumlin@smail.astate.edu (A.S.); steven.chambers@smail.astate.edu (S.A.C.); rawan.alnufaie@smail.astate.edu (R.A.); 2 Department of Biological Sciences, College of Science and Mathematics, Arkansas State University, Jonesboro, AR 72467, USA; dgilmore@astate.edu; 3 Department of Pharmacology and Toxicology, University of Arkansas for Medical Sciences, 4301 W. Markham St., Little Rock, AR 72205, USA; todd.fite@uams.edu (T.F.); basnakianalexeig@uams.edu (A.G.B.); 4 Central Arkansas Veterans Healthcare System, W. 7th St., Little Rock, AR 72205, USA

**Keywords:** Coumarin, pyrazole, hydrazone, MRSA, drug-resistant, *Acinetobacter baumannii*, antimicrobials, ESKAPE pathogen, *Staphylococcus aureus*

## Abstract

Microbial resistance to drugs is an unresolved global concern, which is present in every country. Developing new antibiotics is one of the guidelines of the Centers for Disease Control and Preventions (CDC) to combat bacterial resistance to drugs. Based on our lead molecules, we report the synthesis and antimicrobial studies of 27 new pyrazole derivatives. These new coumarin-pyrazole-hydrazone hybrids are readily synthesized from commercially available starting materials and reagents using benign reaction conditions. All the synthesized molecules were tested against 14 Gram-positive and Gram-negative bacterial strains. Several of these molecules have been found to be potent growth inhibitors of several strains of these tested bacteria with minimum inhibitory concentrations as low as 1.56 μg/mL. Furthermore, active molecules are non-toxic in in vitro and in vivo toxicity studies.

## 1. Introduction

Microbial resistance to antibiotics is a growing public health concern worldwide [1]. Existing antibiotics are losing their effect. There is insufficient investment and research to develop new antibiotics. If this trend continues, the tools to combat antimicrobial resistance will be depleted and many modern medical breakthroughs will be ineffective [2]. Two-thirds of nosocomial drug-resistant infections in the United States are caused by six bacteria informally called the ESKAPE (*Enterococcus faecium*, *Staphylococcus aureus*, *Klebsiella pneumoniae*, *Acinetobacter baumannii*, *Pseudomonas aeruginosa*, and *Enterobacter* species) pathogens. *A. baumannii,* a member of the ESKAPE pathogens, can live in tracheostomy sites or open wounds for several days without causing infection, and this bacterium is life threatening for people with weakened immune systems [3]. Recently, the World Health Organization (WHO) has listed 12 drug resistant bacteria for which new antibiotics are urgently needed. Carbapenem-resistant *A. baumannii* is at the top of the list with critical priority and drug resistant *S. aureus* is in the high priority category [4,5]. *S. aureus*, a Gram-positive bacterium, is found in the nares (nostrils) of about 30% of the population. This bacterium may cause sepsis, pneumonia, endocarditis, and osteomyelitis. *S. aureus* resistance to different antibiotics has evolved into different strains such as methicillin-resistant *S. aureus* (MRSA), vancomycin-intermediate *S. aureus* (VISA), and vancomycin-resistant *S. aureus* (VRSA) [6]. Two percent of the world population are nasal carriers of MRSA. Invasive infection caused by *S. aureus* and its drug resistant strains has decreased over the years but MRSA and other drug resistant strains are still a major threat in healthcare settings [7].

Coumarin derivatives are well-known oxygenated fused bicyclic molecules. These molecules are known for their wide range of biological activities [8,9,10] including activity against Gram-positive [11] bacteria (*S. aureus*, [12] *Bacillus subtilis* [13,14]) and Gram-negative [11] (*E. coli*, [12,14] *P. aeruginosa* [15] and other bacterial species [16]). Coumarin derivatives as anti-*A. baumannii* agents are rare [17]. Pyrazole derivatives are another class of pharmacologically important molecules known for their wide range of therapeutic properties including antimicrobial activities [18,19,20]. Likewise, hydrazone derivatives are known for their wide range of biological activities including antibacterial properties; e.g., rifampicin, an approved drug to treat tuberculosis [21,22].

## 2. Results and Discussion

### 2.1. Chemistry

**Figure 1:**
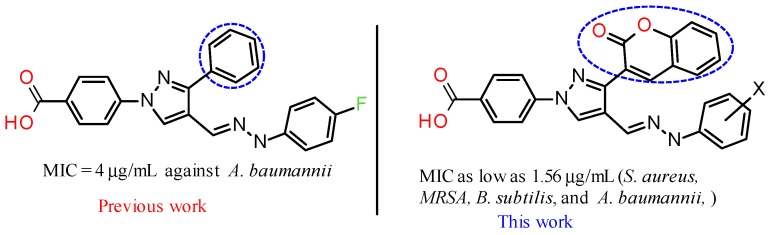
Pyrazole-derived hydrazones as potent antimicrobial agents.

In our quest to develop novel methodologies to synthesize bioactive molecules [23,24,25,26], we have reported the synthesis of novel pyrazole derivatives as potent antimicrobial agents [27,28,29]. Based on our lead molecules and known pharmacological properties of coumarin derivatives, we designed the novel molecules hoping to produce potent antimicrobial agents (Figure 1).

**Scheme 1:**
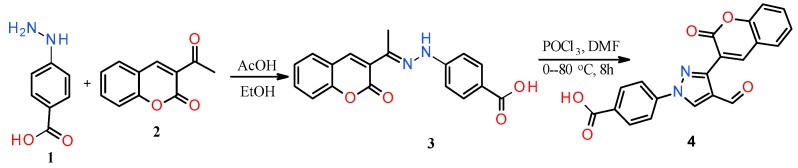
Synthesis of coumarin-substituted pyrazole aldehyde.

The ease of preparation of the aldehyde derivative (**4**) in multi-gram scale without work-up and column purification (Scheme 1) is the key to synthesizing a number of hydrazone derivatives (**5**–**31**) for the study of structure activity relationship (SAR). The reaction of hydrazinobenzoic acid (**1**) with 3-acetylcoumarin (**2**) formed the hydrazone derivative (**3**), which on reaction with in situ generated Vilsmeier reagent afforded the starting material (**4**). 

Designed molecules were synthesized by the reaction of the aldehyde derivative (**4**) with commercially available substituted hydrazines in ethanol to afford products in very good average yield. All the molecules are characterized by ^1^H- and ^13^C-NMR spectroscopy and their structures are confirmed by high resolution mass spectrometry (see Supplementary Materials). Due to the labile nature of the carboxylic acid proton, it does not appear in most of the ^1^H-NMR spectra. All other hydrogens and carbons are accounted for in the spectra. These novel molecules were tested against 14 bacterial strains including ESKAPE pathogens.

### 2.2. Biology

#### 2.2.1. Antimicrobial Studies

The phenyl substituted derivative (**5**) did not show any significant activity against the tested bacteria. *N*-Methyl-*N*-phenyl derivative (**6**) also failed to show any significant activity. *N*-Benzyl-*N*-phenyl derivative (**7**) inhibited the growth of tested Gram-positive strains: *S. aureus* and *B. subtilis* with an MIC value of 6.25 μg/mL. *N*,*N*-Diphenyl substitution (**8**) showed improved activity against *B. subtilis* with an MIC value as low as 3.125 μg/mL. Electron donating substituents such as ethyl (**9**) did not improve the growth inhibition ability of the molecules. Methoxy substitution completely ceased the potency of the resultant compound (**10**). Fluoro substitution showed moderate activity of the products (**11**, **12**, and **13**). Chloro substitution showed mixed results; the meta-chloro derivative (**14**) did not inhibit the bacterial growth significantly but the para-substitution (**15**) showed very good potency of the resultant molecule with MIC values as low as 3.125 μg/mL concentration. A similar pattern was seen for the bromo-substituted compounds (**16** and **17**). Bisfluoro and polyfluorinated compounds (**18**, **19**, and **20**) failed to show any noteworthy activity against the tested bacterial strains. The bischloro substituted derivative (**21**) inhibited the growth of Gram-positive strains with an MIC value of 3.125 μg/mL. The 2-fluoro-3-chloro derivative (**22**) failed to show any remarkable activity while the 3-chloro-4-fluoro substituted compound (**23**) showed moderated activity against *S. aureus*, *B. subtilis*, and *A. baumannii*. The 4-trifluormethyl phenyl hydrazone derivative (**24**) showed selective and potent activity against Gram-positive strains with MIC values as low as 3.125 μg/mL against *B. subtilis.* Other, strong electron withdrawing groups such as nitro (**25**), carboxylic acid (**26** and **27**), and cyano (**28**) terminated the activity of the resultant hydrazones. Alkyl substituted compounds (**29**, **30**, and **31**) also failed to show any activity against the tested strains of bacteria (Table 1).

*Bacillus* species are spore-forming bacteria [30]. Compounds showing activity against *B. subtilis* could have the potential to treat anthrax. Potent compounds such as **8**, **21**, and **24** will be tested against *B. anthracis* Sterne strain in due course. 

Molecules showing activity with MIC values as low as 25 μg/mL against *S. aureus* and *A. baumannii* type strain were tested against four other strains of *Staphylococcus*, three MRSA strains: *S. aureus* ATCC 33591 (MRSA), *S. aureus* ATCC 33592, and *S. aureus* ATCC 43300 (MRSA), plus *S. epidermidis* 700296, and two strains of *A. baumannii*: *A. baumannii* ATCC 747 (human clinical specimen) and *A. baumannii* ATCC BAA-1605 (isolated from a military personnel). It is worth noting that the three MRSA strains possess the SCC mec II or III genomic islands conferring multidrug resistance [31]. The *N*-benzyl-*N*-phenyl derivative (**7**) showed potent activity against two methicillin resistant strains of *S. aureus* with MIC values as low as 3.125 μg/mL. The bisphenyl substituted compound (**8**) inhibited the growth of all the tested strains of *Staphylococcus* with MIC values of 3.125 μg/mL against *S. aureus* ATCC 33592. Potent growth inhibition of this strain is very significant as it is not only methicillin resistant but also gentamicin resistant. Fluoro substituted compounds (**11**, **12**, and **13**) inhibited the tested strains with MIC values as low as 6.25 μg/mL. These molecules showed better growth inhibition activity against Gram-negative bacteria than the Gram-positive strains. Chloro and bromo substitution (**14**, **15**, **16**, and **17**) produced potent activity; para-substituted products showed several times more activity than the meta products with MIC values as low as 1.56 μg/mL. Bis-halo products showed expected activity against the tested strains. Para-chloro and bromo substituted compounds (**15** and **17**) were the best in the series. The bisfluoro derivative (**18**) failed to show any significant activity against these additional strains. Bischloro derivatives (**21**) were found to be potent growth inhibitors of drug resistant *S. aureus* strains with MIC values as low as 3.125 μg/mL. The 3-chloro-2-fluoro derivative (**22**) was active against two strains of *S. aureus* with an MIC value of 3.125 µg/mL. 3-Chloro-4-fluoro substituted compound (**23**) showed broad-spectrum antibiotic activity but with less potency. Last but not least, we found a potent and selective inhibitor of drug resistant Gram-positive strains of *S. aureus*, the 4-trifluoromethyl phenyl derivative (**24**), with an MIC value of 6.25 µg/mL (Table 2).

#### 2.2.2. In vitro Toxicity Studies

**Figure 2:**
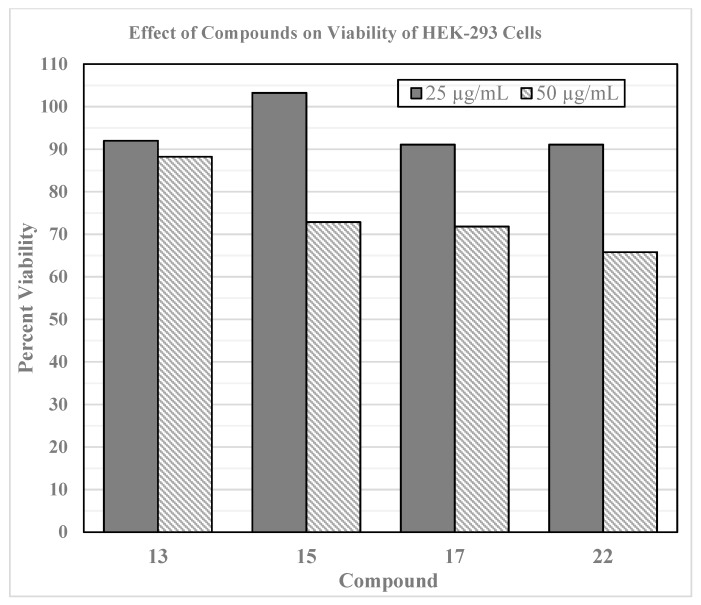
Cytotoxic assay of active compounds on HEK293 cell line by using resazurin assay.

Active molecules were tested against a human embryonic kidney (HEK293) cell line at 25 µg/mL and 50 µg/mL concentrations for in vitro toxicity studies (Figure 2). Potent compounds (**15** and **17**) did not show any significant cytotoxicity at 25 µg/mL concentration. All of the synthesized compounds were submitted to the National Cancer Institute (NCI) for cytotoxicity studies. None of the compounds showed any significant cytotoxicity against NCI-60 cancer cell lines. Thus, these compounds are non-toxic to mammalian cell lines.

#### 2.2.3. Calculated Physicochemical Properties

We calculated the physicochemical properties of the two most potent antimicrobial compounds (**15** and **17**) which are non-toxic to mammalian cell lines (Table 3). The *n*-octanol/water partition coefficient (ilog*P*) is a key parameter for drug design and development. The ilog*P* values are 3.07 and 3.26 for compounds **15** and **17** respectively [32]. Topological total surface area of these molecules is less than 140 Å, which indicates the potential for significant passive transport through the cell membrane [33].

#### 2.2.4. In vivo Toxicity Studies

**Figure 3:**
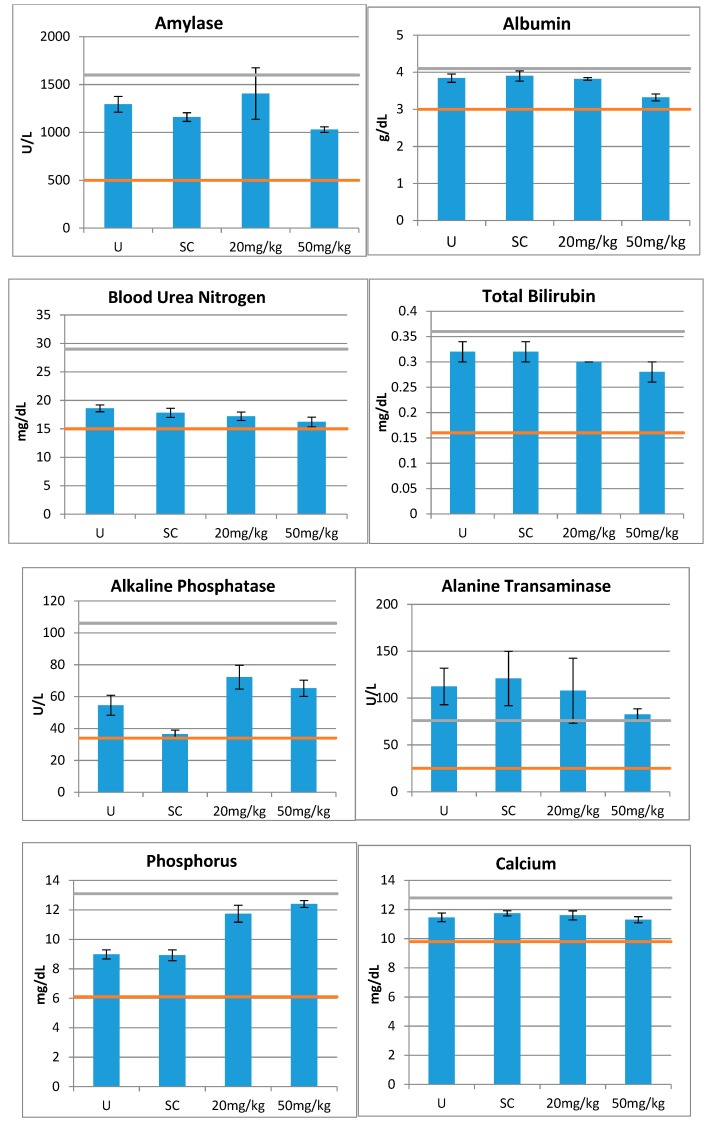

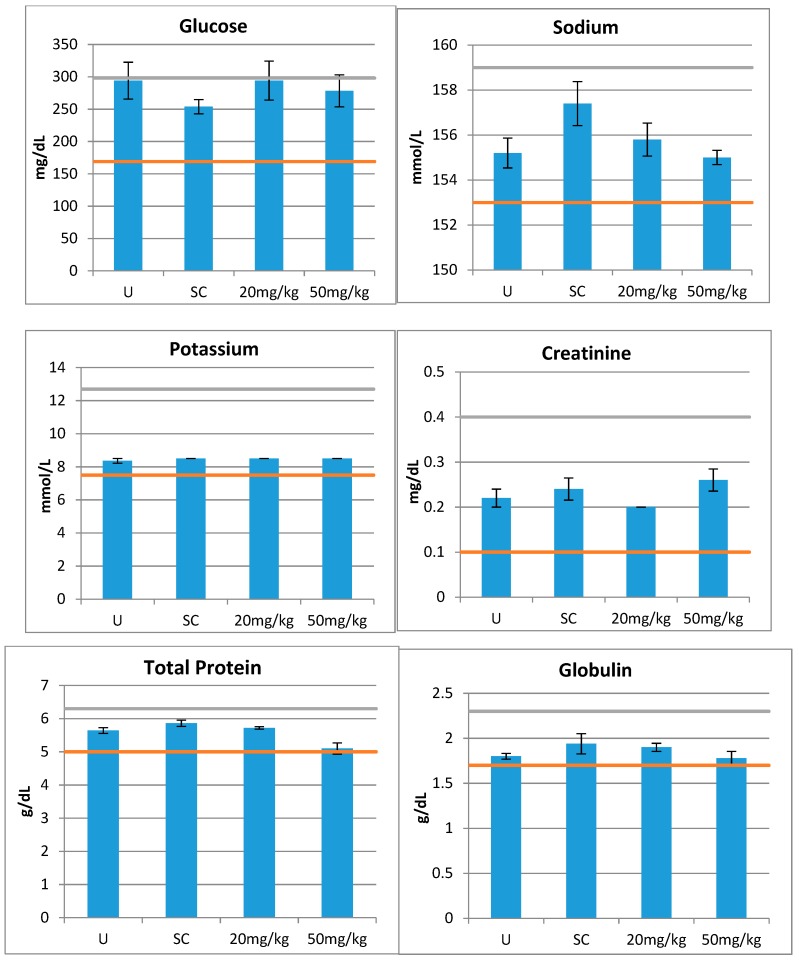
In vivo toxicity of compound **15**. Two doses (20 and 50 mg/kg) were administered IP in CD-1 mice (*n* = 5 for each dose). Blood samples were collected 24 hours later and tested by 14 parameters for organ functions. The orange and green lines indicate the normal ranges for the assays. U = Untreated and SC = Saline Control.

An important condition for eventual use of this compound in humans is the absence of toxicity in vivo animal model studies. Therefore, after demonstrating the absence of toxicity in vitro, we next examined whether compound **15** is toxic to mice. This compound was selected for its potent anti-*A. baumannii* activity, low ilog*P*, and non-toxicity in in vitro studies. In vivo effects of a single IP injection of the compound were assessed by 14 different parameters for organs’ functions as shown in Figure 3. This measurement clearly showed that none of the organ function markers indicated a toxicity by the used criteria. The majority of tests after the administration showed no significant difference from control samples, and none of them were beyond the normal ranges. 

We tested 14 blood markers of organ functions that gave us a toxicity profile of **15** indicative of the complete absence of toxicity at the tested doses. Therefore, we can conclude that this compound is likely to be non-toxic in therapeutic doses. Colistin, the drug used to treat drug-resistant *A. baumannii* infection, will be extremely toxic at these doses [34]. This is a very important discovery, having in mind further potential application of this agent in humans. Certainly, for the development of a final pharmaceutical product, additional tests would be needed to detect pharmacological responses such as absorption, distribution, metabolism, and excretion studies.

## 3. Experimental Section

### 3.1. General Procedures

All the chemicals were purchased from Fisher Chemical (Hanover Park, IL, USA.) and Oakwoochemical (Estill, SC, USA). Commercially available solvents were used without drying. ^1^H- and ^13^C-NMR spectra were obtained with a Varian Mercury-300 MHz in DMSO-*d*_6_ with TMS as internal standard. Purity of the compounds were determined by the ^1^H- and ^13^C-NMR spectroscopy. The ESI-FTMS Mass spectra were recorded Bruker ApexII-FTMS system (Bremen, Germany). 

#### 3.1.1. Synthesis of Hydrazone Derivatives 

A mixture of 4-hydrazinobenzoic acid hydrochloride (**1**, 1.980 g, 10.5 mmol), 3-acetylcoumarin (1.881 g, 10 mmol), and sodium acetate (0.82 g, 10 mmol) in ethanol. Ethanol was evaporated under reduced pressure and the hydrazone derivative was dried in vacuum. The dried material was subjected to further reaction without isolation or purification. The coumarin-derived hydrazone was dissolved in anhydrous *N,N*-dimethyl formamide (30 mL) and sealed by a rubber septum. The solution was cooled under ice for ~10 min, followed by the dropwise addition of phosphorous oxychloride (POCl_3_, 4.67 mL, 50 mmol). The reaction mixture was brought to room temperature and heated 80 °C for eight hours. After completion of the reaction, the reaction mixture was poured onto ice and was stirred for 12 h. The solid product was filtered and washed with water repeatedly followed by drying the product under vacuum to get the pure product in a very good overall yield [27,28,29]. 

#### 3.1.2. Synthesis of Hydrazones

A mixture of the coumarin-derived aldehyde (360 mg, 1 mmol), hydrazine derivative (1.05 mmol) and sodium acetate (86 mg, 1.05 mmol), in case of the hydrochloride salt of the hydrazine derivatives, in anhydrous ethanol was refluxed for 8 h. The solid product was filtered and washed with water (~20 mL), followed by washing with ethanol (~15 mL) to get the pure product, which was dried under vacuum for biological studies.

### 3.2. MIC Studies 

The Minimum Inhibitory Concentration (MIC) of the synthesized compounds was determined by a broth microdilution plate-based technique as per Clinical and Laboratory Standards Institute (CLSI) procedures for antimicrobial susceptibility testing of aerobic bacteria. In brief, bacteria were streaked onto Tryptic Soy Agar (TSA) or TSA with 5% sheep blood and incubated at 35 °C overnight. Colonies were suspended in sterile saline (0.9% NaCl) to match a 0.5 MacFarland standard then diluted 1:100 with Mueller Hinton broth to an estimated 1 × 10^6^ CFU/mL concentration. Compounds to be tested were dissolved in DMSO to a 2 mg/mL concentration and diluted serially 1:2 in microplates such that after the addition of bacteria in broth the final concentrations began at 50 µg/mL. Inhibition of bacterial growth was determined using resazurin as a marker for cell viability [35]. Resazurin was added with bacteria to 8 µg/mL final concentration, and plates were incubated at 35 °C for 20–24 h and read visually. Negative controls (bacteria without inhibitors) and positive controls (bacteria plus a serially diluted antibiotic) were included on every plate. The MIC was determined as the lowest concentration where there was no color change of resazurin from purple to red (evidence of bacterial growth) [28,29].

### 3.3. Cytotoxicity Studies

In vitro toxicity of the active compounds were carried out according to our recently reported modified procedure [28,29]. HEK293 cells were counted using a Countess automated cell counter and 5000 cells/well were plated in black 96 well plates. Compounds were added in triplicate at 50 µg/mL and 25 µg/mL concentrations and incubated for 24 h followed by adding the 5% resazurin solution for a final volume of 200 µL in each well. Resazurin containing plates were incubated for four and six hours before the taking the readings for cell viability. Excitation and emission for fluorescence were measured at 544 nm and 590 nm respectively using a BMG Labtech Fluostar Optima plate reader. 

### 3.4. In Vivo Toxicity Assessment

All animal experiments were performed at the Central Arkansas Veterans Healthcare System (John L. McClellan Memorial Veterans Hospital in Little Rock, AR, USA) and have been approved by the Institutional Animal Care and Use Committee. CD1 male mice (8 weeks old, 33–37 g) were purchased from Charles River Laboratories (Wilmington, MA, USA). Compound (15) was freshly dissolved in 0.9% saline, sterilized by ultrafiltration, and injected intraperitoneally (IP) in mice at two doses of 20 or 50 mg/kg (*n* = 5 per dose). The two control groups (*n* = 5/group) were untreated of administered with the vehicle (saline). The mice were euthanized 24 h after the injection, and blood was collected by cardiac puncture. Toxicity was assessed by measuring 14 blood markers of various organ function available in the Comprehensive Diagnosis Kit (Abaxis, Union City, CA, USA) and using VetScan VS2 instrument (Abaxis). The markers included: alanine aminotransferase (ALT), albumin (ALB), alkaline phosphatase (ALP), amylase (AMY), calcium (CA), creatinine (CRE), globulin (GLOB), glucose (GLU), phosphorus (PHOS), potassium (K^+^), sodium (Na^+^), total bilirubin (TBIL), total protein (TP), and blood urea nitrogen (BUN) measured by VetScan VS2 (Abaxis). Our criteria for toxicity included measurements being beyond the normal ranges, plus statistically significantly different from the untreated and vehicle (saline) controls. 

### 3.5. Experimental Data 

*4-[4-Formyl-3-(2-oxochromen-3-yl)pyrazol-1-yl]benzoic acid* (**4**). Whitish solid, yield: 92% (3.31 g) ^1^H-NMR (300 MHz, DMSO-*d*_6_): δ 9.93 (s, 1H), 9.35 (s, 1H), 8.47 (s, 1H), 8.12–8.05 (m, 4H), 7.87 (d, *J* = 6.7 Hz, 1H), 7.72–7.68 (m, 1H), 7.51 (d, *J* = 8.2 Hz, 1H), 7.45–7.40 (m, 1H); ^13^C-NMR (75 MHz, DMSO-*d*_6_) δ 186.0, 167.3, 159.8, 154.0, 148.2, 143.7, 141.4, 133.5, 133.2, 131.3, 129.6, 125.4, 124.2, 120.1, 119.3, 119.2, 116.7, 115.2. HRMS (ESI-FTMS Mass (*m*/*z*): calcd for C_20_H_12_N_2_O_5_ [M + H]^+^ = 361.0819, found 361.0816. 

*4-[3-(2-Oxochromen-3-yl)-4-[(E)-(phenylhydrazono)methyl]pyrazol-1-yl]benzoic acid* (**5**). Yellowish; yield: 82% (369 mg). ^1^H-NMR (300 MHz, DMSO-*d*_6_): δ 10.20 (s, 1H), 8.97 (s, 1H), 8.35 (s, 1H), 8.08 (s, 5H), 7.87–7.83 (m, 2H), 7.71–7.68 (m, 1H), 7.54–7.51 (m, 1H), 7.43 (t, *J* = 6.8 Hz, 1H), 7.00–6.95 (m, 2H), 6.75 (d, *J* = 7.4 Hz, 2H), 6.62 (t, *J* = 6.3 Hz, 1H); ^13^C-NMR (75 MHz, DMSO-*d*_6_) δ 167.1, 159.4, 153.9, 146.3, 145.6, 143.0, 142.4, 132.8, 131.4, 129.3, 129.2, 129.0, 127.2, 125.3, 122.1, 121.3, 119.3, 118.7, 118.3, 116.5, 112.0. HRMS (ESI-FTMS Mass (*m*/*z*): calcd for C_26_H_19_N_4_O_4_ [M + H]^+^ = 451.1401, found 451.1395.

*4-[4-[(E)-[Methyl(phenyl)hydrazono]methyl]-3-(2-oxochromen-3-yl)pyrazol-1-yl]benzoic acid* (**6**). Shiny yellow; yield: 90% (417 mg). ^1^H-NMR (300 MHz, DMSO-*d*_6_): δ 8.93 (s, 1H), 8.35 (s, 1H), 8.09 (s, 4H), 7.84–7.73 (m, 1H), 7.71–7.68 (m, 1H), 7.62 (s, 1H), 7.51–7.41 (m, 2H), 7.08–7.05 (m, 2H), 6.96–6.72 (m, 2H), 6.72 (t, *J* = 7.1 Hz, 1H), 3.30 (s, 3H); ^13^C-NMR (75 MHz, DMSO-*d*_6_) δ 167.1, 159.5, 153.9, 147.5, 146.3, 142.9, 142.5, 132.6, 131.4, 129.2, 129.0, 128.8, 127.2, 125.4, 125.2, 122.6, 122.1, 120.0, 119.4, 118.3, 116.6, 114.5, 32.7. HRMS (ESI-FTMS Mass (*m*/*z*): calcd for C_27_H_20_N_4_O_4_ [M + H]^+^ = 465.1557, found 465.1545.

*4-[4-[(E)-[Benzyl(phenyl)hydrazono]methyl]-3-(2-oxochromen-3-yl)pyrazol-1-yl]benzoic acid***(7).** Yellow solid; yield: 92% (496 mg). ^1^H-NMR (300 MHz, DMSO-*d*_6_): δ 8.87 (s, 1H), 8.31 (s, 1H), 8.09-8.04 (m, 4H), 7.74 (d, *J* = 7.3 Hz, 1H), 7.74–7.69 (m, 1H), 7.58 (s, 1H), 7.51–7.42 (m, 2H), 7.30–7.21 (m, 3H), 7.13 (d, *J* = 7.0 Hz, 2H), 6.98–6.96 (m, 2H), 6.87–6.83 (m, 2H), 6.71 (t, *J* = 6.4 Hz, 1H), 5.19 (s, 2H); ^13^C-NMR (75 MHz, DMSO-*d*_6_) δ 167.1, 159.3, 153.8, 147.1, 146.2, 142.7, 142.4, 139.8, 136.2, 132.7, 131.4, 129.2, 129.1, 128.8, 127.8, 127.4, 126.6, 125.2, 122.8, 121.7, 120.1, 119.3, 118.3, 116.6, 113.9, 48.2. HRMS (ESI-FTMS Mass (*m*/*z*): calcd for C_20_H_12_N_2_O_5_ [M + H]^+^ = 541.1870, found 541.1860.

*4-[4-[(E)-(Diphenylhydrazono)methyl]-3-(2-oxochromen-3-yl)pyrazol-1-yl]benzoic acid* (**8**). Orange; yield: 91% (478 mg). ^1^H-NMR (300 MHz, DMSO-*d*_6_): δ 8.92 (s, 1H), 8.35 (s, 1H), 8.09–7.98 (m, 4H), 7.87 (d, *J* = 7.4 Hz, 1H), 7.75–7.70 (m, 1H), 7.52–7.43 (m, 2H), 7.27–7.22 (m, 5H), 7.16–7.09 (m, 2H), 6.93 (d, *J* = 7.7 Hz, 4H); ^13^C-NMR (75 MHz, DMSO-*d*_6_) δ 167.0, 159.3, 153.8, 146.1, 143.1, 142.9, 142.4, 132.8, 131.4, 130.2, 129.3, 128.9, 128.5, 128.0, 125.3, 124.8, 122.6, 122.2, 121.0, 119.3, 118.2, 116.6. HRMS (ESI-FTMS Mass (*m*/*z*): calcd for C_32_H_22_N_4_O_4_ [M + H]^+^ = 527.1714, found 527.1699.

*4-[4-[(E)-[(2-Ethylphenyl)hydrazono]methyl]-3-(2-oxochromen-3-yl)pyrazol-1-yl]benzoic acid* (**9**). Yellow solid; yield: 80% (382 mg). ^1^H-NMR (300 MHz, DMSO-*d*_6_): δ 9.48 (s, 1H), 8.98 (s, 1H), 8.53 (s, 1H), 8.12–8.09 (m, 6H), 7.86 (d, *J* = 7.6 Hz, 1H), 7.71 (t, *J* = 8.1 Hz, 1H), 7.52 (d, *J* = 8,2 Hz,1H), 7.44 (t, *J* = 7.4 Hz,1H), 6.96 (t, *J* = 7.2 Hz, 2H), 6.69–6.59 (m, 2H), 1.11 (t, *J* = 7.3 Hz, 3H); ^13^C-NMR (75 MHz, DMSO-*d*_6_) δ 167.1, 159.4, 153.9, 146.4, 143.1, 142.8, 142.4, 132.8, 131.4, 130.3, 129.3, 128.6, 127.1, 126.5, 125.3, 122.2, 121.5, 119.3, 119.0, 118.3, 116.6, 112.3. HRMS (ESI-FTMS Mass (*m*/*z*): calcd for C_28_H_22_N_4_O_4_ [M + H]^+^ = 479.1714, found 479.1700.

*4-[4-[(E)-[(4-Methoxyphenyl)hydrazono]methyl]-3-(2-oxochromen-3-yl)pyrazol-1-yl]benzoic acid* (**10**). Orange solid; yield: 83% (398 mg). ^1^H-NMR (300 MHz, DMSO-*d*_6_): δ 9.96 (s, 1H), 8.93 (s,1H), 8.34 (s, 1H), 8.08 (s, 4H), 7.85 (d, *J* = 7.5 Hz, 1H), 7.77–7.69 (m, 2H), 7.53 (d, *J* = 8.2 Hz, 1H), 7.44 (t, *J* = 7.4 Hz,1H), 6.72–6.69 (m, 2H), 6.61–6.58 (m,1H); ^13^C-NMR (75 MHz, DMSO-*d*_6_) δ 167.1, 159.4, 153.9, 152.7, 146.2, 143.0, 142.5, 139.7, 132.7, 131.4, 129.3, 128.9, 127.9, 126.8, 125.3, 122.2, 121.5, 119.4, 118.3, 116.6, 114.7, 113.0. HRMS (ESI-FTMS Mass (*m*/*z*): calcd for C_27_H_20_N_4_O_5_ [M + H]^+^ = 481.1506, found 481.1495.

*4-[4-[(E)-[(2-Fluorophenyl)hydrazono]methyl]-3-(2-oxochromen-3-yl)pyrazol-1-yl]benzoic acid* (**11**). Orange solid; yield: 80% (374 mg). ^1^H-NMR (300 MHz, DMSO-*d*_6_): δ 10.12 (s, 1H), 9.01 (s, 1H), 8.35 (s, 1H), 8.11–8.09 (m, 5H), 7.86 (d, *J* = 7.6 Hz, 1H), 7.71 (t, *J* = 7.7 Hz, 1H), 7.53 (d, *J* = 8.3 Hz, 1H), 7.44 (t, *J* = 7.3 Hz, 1H) 7.09–6.96 (m, 2H), 6.71–6.66 (m, 1H); ^13^C-NMR (75 MHz, DMSO-*d*_6_) δ 167.1, 159.4, 153.9, 150.9, 147.7, 146.4, 143.1, 142.3, 133.7 (d, *J* = 9.8 Hz), 132.4 (d, *J* = 64.2 Hz), 131.4, 129.3, 127.6, 125.3, 124.8, 122.1, 121.0, 119.3, 118.4, 116.5, 115.3 (d, *J* = 16.9 Hz), 113.8. HRMS (ESI-FTMS Mass (*m*/*z*): calcd for C_26_H_17_N_4_O_4_F [M + H]^+^ = 469.1307, found 469.1292.

*4-[4-[(E)-[(3-Fluorophenyl)hydrazono]methyl]-3-(2-oxochromen-3-yl)pyrazol-1-yl]benzoic acid* (**12**). Off orange; yield: 78% (365 mg). ^1^H-NMR (300 MHz, DMSO-*d*_6_): δ 10.42 (s, 1H), 9.02 (s, 1H), 8.36 (s, 1H), 8.09 (s, 4H), 7.89–7.84 (m, 2H), 7.69 (t, *J* = 7.8 Hz, 1H), 7.51–7.39 (m, 2H), 7.07–6.99 (m, 1H), 6.59–6.54 (m, 2H), 6.40 (t, *J* = 7.9 Hz, 1H); ^13^C-NMR (75 MHz, DMSO-*d*_6_) δ 167.1, 165.3, 162.1, 159.4, 153.8, 147.6 (d, *J* = 11.0 Hz), 146.4, 142.8 (d, *J* = 54.5 Hz), 132.8, 131.4, 130.8 (d, *J* = 9.9 Hz), 130.4, 129.3, 129.0, 127.6, 125.3, 121.9, 120.9, 119.3, 118.4, 116.6, 108.2, 104.8 (d, *J* = 21.3 Hz), 98.5 (d, *J* = 26.3 Hz). HRMS (ESI-FTMS Mass (*m*/*z*): calcd for C_26_H_17_N_4_O_4_F [M + H]^+^ = 469.1307, found 469.1294.

*4-[4-[(E)-[(4-Fluorophenyl)hydrazono]methyl]-3-(2-oxochromen-3-yl)pyrazol-1-yl]benzoic acid* (**13**). Yellow solid; yield: 75% (355 mg). ^1^H-NMR (300 MHz, DMSO-*d*_6_): δ 10.18 (s, 1H), 8.97 (s, 1H), 8.35 (s, 1H), 8.08 (s, 4H), 7.87–7.82 (m, 2H), 7.71 (t, *J* = 7.6 Hz, 1H), 7.54 (d, *J* = 8.3 Hz, 1H), 7.44 (t, *J* = 7.4 Hz, 1H), 6.84–6.78 (m, 4H); ^13^C-NMR (75 MHz, DMSO-*d*_6_) δ 167.1, 159.4, 153.8, 146.3, 143.1, 142.4, 142.3, 132.8, 131.4, 129.3, 129.0, 127.1, 125.3, 122.0, 121.2, 119.3, 118.4, 116.6, 115.7 (d, *J* = 22.2 Hz), 112.9 (d, *J* = 7.2 Hz). HRMS (ESI-FTMS Mass (*m*/*z*): calcd for C_26_H_17_N_4_O_4_F [M + H]^+^ = 469.1307, found 469.1294.

*4-[4-[(E)-[(3-Chlorophenyl)hydrazono]methyl]-3-(2-oxochromen-3-yl)pyrazol-1-yl]benzoic acid* (**14**). Brown solid; yield: 81% (382 mg). ^1^H-NMR (300 MHz, DMSO-*d*_6_): δ 10.57 (s, 1H), 9.02 (s, 1H), 8.36 (s, 1H), 8.08 (s, 4H), 7.90 (s, 1H), 7.86 (d, *J* = 7.5 Hz, 1H), 7.71–7.66 (m, 1H), 7.50 (d, *J* = 8.2 Hz, 1H), 7.42 (t, *J* = 7.4 Hz, 1H), 7.02 (t, *J* = 7.9 Hz, 1H), 6.79 (s, 1H), 6.67 (d, *J* = 8.2 Hz, 1H), 6.63 (d, *J* = 7.7 Hz, 1H); ^13^C-NMR (75 MHz, DMSO-*d*_6_) δ 167.1, 159.4, 153.9, 147.0, 146.3, 143.1, 142.3, 134.2, 132.7, 131.4, 130.8, 130.6, 129.3, 129.2, 127.8, 125.3, 122.0, 120.9, 119.3, 118.4, 118.1, 116.8, 111.1, 110.8. HRMS (ESI-FTMS Mass (*m*/*z*): calcd for C_26_H_17_N_4_O_4_Cl [M + H]^+^ = 469.1307, found 469.1296.

*4-[4-[(E)-[(4-Chlorophenyl)hydrazono]methyl]-3-(2-oxochromen-3-yl)pyrazol-1-yl]benzoic acid* (**15**). Orange solid; yield: 77% (372 mg). ^1^H-NMR (300 MHz, DMSO-*d*_6_): δ 13.10 (br s, 1H), 10.35 (s, 1H), 9.00 (s, 1H), 8.37 (s,1H), 8.09 (s, 4H), 7.87–7.84 (m, 2H), 7.72 (t, *J* = 7.5 Hz, 1H), 7.54 (d, *J* = 8.2 Hz, 1H), 7.44 (t, *J* = 7.4 Hz, 1H), 7.02 (d, *J* = 8.7 Hz, 2H), 6.78 (d, *J* = 8.7 Hz, 2H); ^13^C-NMR (75 MHz, DMSO-*d*_6_) δ 167.0, 159.4, 153.9, 146.4, 144.5, 143.2, 142.4, 132.9, 131.4, 130.1, 129.3, 129.05, 129.01, 127.4, 125.3, 121.94, 121.90, 121.0, 119.3,118.4, 116.6, 113.4. HRMS (ESI-FTMS Mass (*m*/*z*): calcd for C_26_H_17_N_4_O_4_Cl [M + H]^+^ = 469.1307, found 469.1292, Yield = 95%.

*4-[4-[(E)-[(3-Bromo)hydrazono]methyl]-3-(2-oxochromen-3-yl)pyrazol-1-yl]benzoic acid* (**16**). Brown solid; yield: 83% (438 mg). ^1^H-NMR (300 MHz, DMSO-*d*_6_): δ 13.12 (br s, 1H), 10.40 (s, 1H), 9.03 (s, 1H), 8.37 (s, 1H), 8.09 (s, 4H), 7.87–7.84 (m, 2H), 7.68 (t, *J* = 7.7 Hz, 1H), 7.52 (d, *J* = 8.2 Hz, 1H), 7.42 (t, *J* = 7.4 Hz, 1H), 7.00–6.95 (m, 2H), 6.78 (d, *J* = 7.8 Hz, 1H), 6.72 (d, *J* = 8.1 Hz, 1H); ^13^C-NMR (75 MHz, DMSO-*d*_6_) δ 167.0, 159.4, 153.9, 147.1, 146.4, 143.2, 142.4, 132.7, 131.4, 131.1, 130.8, 129.3, 129.0, 127.8, 125.3, 122.9, 121.9, 121.0, 120.8, 119.3, 118.4, 116.8, 114.0, 111.2. HRMS (ESI-FTMS Mass (*m*/*z*): calcd for C_26_H_17_N_4_O_4_Br [M + H]^+^ = 531.0487, found 531.0473.

*4-[4-[(E)-[(4-Bromophenyl)hydrazono]methyl]-3-(2-oxochromen-3-yl)pyrazol-1-yl]benzoic acid* (**17**). Yellow solid; yield: 81% (427 mg). ^1^H-NMR (300 MHz, DMSO-*d*_6_): δ 10.35 (s, 1H), 9.00 (s, 1H), 8.37 (s, 1H), 8.09 (s, 4H), 7.87–7.85 (m, 2H), 7.72 (t, *J* = 7.1 Hz, 1H), 7.55 (d, *J* = 8.2 Hz, 1H), 7.44 (t, *J* = 7.4 Hz, 1H), 7.14 (d, *J* = 8.7 Hz, 2H), 6.73 (d, *J* = 8.8 Hz, 2H); ^13^C-NMR (75 MHz, DMSO-*d*_6_) δ 167.0, 159.4, 153.9, 146.4, 144.9, 143.2, 142.4, 132.9, 131.8, 131.4, 130.2, 129.3, 129.0, 127.4, 125.3, 121.8, 121.0, 119.3, 118.4, 116.6, 113.9, 109.5. HRMS (ESI-FTMS Mass (*m*/*z*): calcd for C_26_H_17_N_4_O_4_Br [M + H]^+^ = 531.0487, found 531.0471

*4-[4-[(E)-[(2,5-Difluorophenyl)hydrazono]methyl]-3-(2-oxochromen-3-yl)pyrazol-1-yl]benzoic acid* (**18**). Reddish solid; yield: 78% (377 mg). ^1^H-NMR (300 MHz, DMSO-*d*_6_): δ 10.36 (s,1H), 9.09 (s, 1H), 8.37 (s, 1H), 8.13–8.09 (m, 5H), 7.85 (d, *J* = 7.2 Hz, 1H), 7.68 (t, *J* = 7.2 Hz, 1H), 7.49–7.41 (m, 2H), 7.10 (br s, 1H), 6.77 (br s, 1H), 6.40 (br s, 1H); ^13^C-NMR (75 MHz, DMSO-*d*_6_) δ 167.0, 159.5 (d, *J* = 235.7 Hz), 159.4, 153.8, 147.0, 146.5, 143.9, 143.3, 142.4, 135.1 (t, *J* = 11.8 Hz), 133.3, 132.9, 131.4, 129.3, 129.0, 128.0, 125.3, 121.7, 120.7, 119.2, 118.4, 116.6-116.0 (m), 103.8 (d, *J* = 24.0 Hz), 100.2 (d, *J* = 26.0 Hz). HRMS (ESI-FTMS Mass (*m*/*z*): calcd for C_26_H_17_N_4_O_4_F_2_ [M + H]^+^ = 487.1212, found 487.1199.

*4-[3-(2-Oxochromen-3-yl)-4-[(E)-[(2,3,5,6-tetrafluorophenyl)hydrazono]methyl]pyrazol-1-yl]benzoic acid* (**19**). Yellow solid; yield: 89% (464 mg). ^1^H-NMR (300 MHz, DMSO-*d*_6_): δ 10.28 (s, 1H), 8.94 (s, 1H), 8.31 (s, 1H), 8.17 (s, 1H), 8.05 (s, 3H), 7.82 (d, *J* = 7.5 Hz, 1H), 7.67 (t, *J* = 7.4 Hz, 1H), 7.46–7.37 (m, 2H), 7.14–7.02 (m, 1H); ^13^C-NMR (75 MHz, DMSO-*d*_6_) δ 167.0, 159.4, 154.0, 148.1, 142.1, 142.3, 135.1, 132.6, 131.4, 129.3-129.2 (m), 128.1, 125.1, 121.7, 120.2, 119.3, 118.5, 116.4, 95.2 (t, *J* = 23.5 Hz). HRMS (ESI-FTMS Mass (*m*/*z*): calcd for C_26_H_15_N_4_O_4_F_4_ [M + H]^+^ = 523.1024, found 523.1008.

*4-[3-(2-Oxochromen-3-yl)-4-[(E)-[(2,3,4,5,6-pentafluorophenyl)hydrazono]methyl]pyrazol-1-yl]benzoic acid* (**20**). Yellow solid; yield: 90% (486 mg). ^1^H-NMR (300 MHz, DMSO-*d*_6_): δ 10.12 (s, 1H), 8.92 (s, 1H), 8.30 (s, 1H), 8.13–8.07 (m, 5H), 7.82 (d, *J* = 7.6 Hz, 1H), 7.67 (t, *J* = 7.6 Hz, 1H), 7.47–7.37 (m, 2H); ^13^C-NMR (75 MHz, DMSO-*d*_6_) δ 167.1, 159.4, 154.0, 146.6, 143.1, 142.3, 135.1, 132.6, 131.3, 129.5, 129.2, 128.0, 125.1, 121.6, 119.6, 119.3, 118.5, 116. HRMS (ESI-FTMS Mass (*m*/*z*): calcd for C_26_H_14_N_4_O_4_F_5_ [M + H]^+^ = 541.0930, found 541.0919.

*4-[4-[(E)-[(2,4-Dichlorophenyl)hydrazono]methyl]-3-(2-oxochromen-3-yl)pyrazol-1-yl]benzoic acid* (**21**). Brownish solid; yield: 85% (440 mg). ^1^H-NMR (300 MHz, DMSO-*d*_6_): δ 9.94 (s, 1H), 9.05 (s, 1H), 8.37 (s, 1H), 8.27 (s, 1H), 8.09 (s, 4H), 7.86 (d, *J* = 7.7 Hz, 1H), 7.72 (t, *J* = 7.4 Hz, 1H), 7.54 (d, *J* = 8.1 Hz, 1H), 7.47–7.39 (m, 2H), 7.11 (d, *J* = 8.8 Hz, 1H), 6.89 (d, *J* = 8.8 Hz, 1H); ^13^C-NMR (75 MHz, DMSO-*d*_6_) δ 167.1, 159.4, 153.9, 146.6, 143.4, 142.3, 140.9 133.9, 132.9, 131.4, 129.4, 129.0, 127.8, 125.4, 122.2, 121.7, 120.7, 119.3, 118.5, 116.8, 116.6, 114.9. HRMS (ESI-FTMS Mass (*m*/*z*): calcd for C_26_H_17_N_4_O_4_Cl_2_ [M + H]^+^ = 519.0621, found 519.0615.

*4-[4-[(E)-[(3-Chloro-2-fluoro-phenyl)hydrazono]methyl]-3-(2-oxochromen-3-yl)pyrazol-1-yl]benzoic acid* (**22**). Orange solid; yield: 72% (361 mg). ^1^H-NMR (300 MHz, DMSO-*d*_6_): δ 10.37 (s, 1H), 9.04 (s, 1H), 8.35 (s, 1H), 8.14 (s, 1H), 8.09 (s, 4H), 7.86 (d, *J* = 7.3 Hz, 1H), 7.73–7.68 (m, 1H), 7.53 (d, *J* = 8.1 Hz, 1H), 7.45–7.41 (m, 1H), 6.98 (m, 1H), 6.78–6.67 (m, 2H); ^13^C-NMR (75 MHz, DMSO-*d*_6_) δ 167.1, 159.4, 153.8, 146.5, 144.68 (^1^*J*_C-F_ = 239.8 Hz), 143.2, 142.4, 135.3, 135.1, 133.1 (^2^*J*_C-F_ = 22.3 Hz), 131.4, 129.3 (^2^*J*_C-F_ = 22.7 Hz), 127.9, 125.3, 121.8, 120.7, 120.0, 119.8, 119.3, 118.7, 118.4, 116.5, 112.5. HRMS (ESI-FTMS Mass (*m*/*z*): calcd for C_26_H_17_N_4_O_4_ClF [M + H]^+^ = 503.0917, found 503.0903.

*4-[4-[(E)-[(3-chloro-4-fluoro-phenyl)hydrazono]methyl]-3-(2-oxochromen-3-yl)pyrazol-1-yl]benzoic acid* (**23**). Yellow solid; yield: 76% (381 mg). ^1^H-NMR (300 MHz, DMSO-*d*_6_): δ 10.35 (s, 1H), 9.01 (s, 1H), 8.36 (s, 1H), 8.08–8.07 (m, 4H), 7.86–7.84 (m, 2H), 7.69 (t, *J* = 7.5 Hz, 1H), 7.51 (d, *J* = 8.2 Hz, 1H), 7.42 (t, *J* = 7.4 Hz, 1H), 7.09 (t, *J* = 8.9 Hz, 1H), 6.88–6.87 (m, 1H), 6.71–6.68 (m, 1H); ^13^C-NMR (75 MHz, DMSO-*d*_6_) δ 167.1, 159.4, 153.9, 152.4, 149.2, 146.3, 143.3, 142.3, 132.8, 131.4, 130.7, 129.4, 129.3, 127.7, 125.3, 121.9, 120.8, 120.2 (d, *J* = 18.3 Hz), 119.3, 118.4, 117.4 (d, *J* = 21.6 Hz), 116.8, 112.4, 111.8 (d, *J* = 6.4 Hz). HRMS (ESI-FTMS Mass (*m*/*z*): calcd for C_26_H_17_N_4_O_4_ClF [M + H]^+^ = 503.0917, found 503.0910.

*4-[3-(2-oxochromen-3-yl)-4-[(E)-[[4-(trifluoromethyl)phenyl]hydrazono]methyl]pyrazol-1-yl]benzoic acid* (**24**). Yellow solid; yield: 81% (419 mg). ^1^H-NMR (300 MHz, DMSO-*d*_6_): δ 10.68 (s, 1H), 9.03 (s, 1H), 8.37 (s, 1H), 8.09 (s, 4H), 7.92 (s, 1H), 7.87 (d, *J* = 7.5 Hz, 1H), 7.74–7.69 (m, 1H), 7.54 (d, *J* = 8.1 Hz, 1H), 7.46–7.41 (m, 1H), 7.31 (d, *J* = 8.3 Hz, 2H), 6.91 (d, *J* = 8.4 Hz, 2H); ^13^C-NMR (75 MHz, DMSO-*d*_6_) δ 167.1, 159.5, 153.9, 148.5, 146.5, 143.3, 142.4, 132.9, 131.9, 131.4, 129.4, 129.1, 127.7, 127.1, 126.6, 125.3, 121.7, 120.7, 119.3, 118.2, 116.6, 111.6. HRMS (ESI-FTMS Mass (*m*/*z*): calcd for C_27_H_18_N_4_O_4_F_3_ [M + H]^+^ = 519.1275, found 519.1262.

*4-[4-[(E)-[(4-nitrophenyl)hydrazono]methyl]-3-(2-oxochromen-3-yl)pyrazol-1-yl]benzoic acid* (**25**). Reddish; yield: 89% (440 mg). ^1^H-NMR (300 MHz, DMSO-*d*_6_): δ 11.23 (s, 1H), 9.13 (s, 1H), 8.42 (s, 1H), 8.11 (s, 4H), 8.04 (s, 1H), 7.96–7.87 (m, 3H), 7.74 (t, *J* = 7.3 Hz, 1H), 7.56 (d, *J* = 8.3 Hz, 1H), 7.46 (t, *J* = 7.4 Hz, 1H), 6.91 (d, *J* = 9.2, 2H); ^13^C-NMR (75 MHz, DMSO-*d*_6_) δ 167.0, 159.5, 153.9, 150.8, 146.7, 143.4, 142.3, 138.4, 134.9, 133.0, 131.4, 129.4, 129.2, 128.3, 126.3, 125.4, 121.4, 120.2, 119.3, 118.5, 116.6, 111.2. HRMS (ESI-FTMS Mass (*m*/*z*): calcd for C_26_H_18_N_5_O_6_ [M + H]^+^ = 496.1252, found 496.1240.

*4-[(2E)-2-[[1-(4-carboxyphenyl)-3-(2-oxochromen-3-yl)pyrazol-4-yl]methylene]hydrazino]benzoic acid* (**26**). Brownish solid; yield: 83% (410 mg). ^1^H-NMR (300 MHz, DMSO-*d*_6_): δ 10.39 (s, 1H) 8.99 (s, 1H), 8.38 (s, 1H), 8.09 (s, 4H), 7.86–7.85 (m, 2H), 7.72–7.68 (m, 1H), 7.52 (d, *J* = 8.2 Hz, 1H), 7.45–7.42 (m, 2H), 7.24 (d, *J* = 7.3 Hz, 1H), 7.10–6.99 (m, 2H); ^13^C-NMR (75 MHz, DMSO-*d*_6_) δ 167.9, 167.1, 159.5, 153.9, 146.4, 145.8, 143.3, 142.4, 132.9, 132.0, 131.4, 130.3, 129.4, 129.3, 129.2, 127.3, 125.3, 121.7, 120.9, 119.7, 119.3, 118.4, 116.6, 115.9, 112.9. HRMS (ESI-FTMS Mass (*m*/*z*): calcd for C_27_H_19_N_4_O_6_ [M + H]^+^ = 495.1299, found 495.1284.

*4-[(2E)-2-[[1-(4-carboxyphenyl)-3-(2-oxochromen-3-yl)pyrazol-4-yl]methylene]hydrazino]benzoic acid* (**27**). Orange solid; yield: 82% (405 mg). ^1^H-NMR (300 MHz, DMSO-*d*_6_): δ 10.73 (s, 1H), 8.97 (s, 1H), 8.37 (s, 1H), 8.08 (d, *J* = 8.5 Hz, 2H), 8.01 (d, *J* = 9.0 Hz, 3H), 7.87 (d, *J* = 7.1 Hz, 1H), 7.72 (t, *J* = 8.1 Hz, 1H), 7.62–7.52 (m, 3H), 7.44 (t, *J* = 7.5 Hz, 1H), 6.81 (d, *J* = 8.6 Hz, 2H); ^13^C-NMR (75 MHz, DMSO-*d*_6_) δ 168.3, 167.9, 159.5, 153.9, 149.0, 146.0, 143.1, 141.0, 134.3, 132.8, 131.3, 131.1, 129.3, 127.6, 125.3, 122.1, 121.0, 120.5, 119.4, 118.0, 116.5, 111.1. HRMS (ESI-FTMS Mass (*m*/*z*): calcd for C_27_H_19_N_4_O_6_ [M + H]^+^ = 495.1299, found 495.1290.

*4-[(2E)-2-[[1-(4-cyanophenyl)-3-(2-oxochromen-3-yl)pyrazol-4-yl]methylene]hydrazino]benzoic acid* (**28**). Off yellow solid; yield: 78% (370 mg). ^1^H-NMR (300 MHz, DMSO-*d*_6_): δ 10.87 (s, 1H), 9.07 (s, 1H), 8.39 (s, 1H), 8.12-8.09 (m, 4H), 7.95 (s, 1H), 7.87 (d, *J* = 7.5 Hz, 1H), 7.75–7.70 (m, 1H), 7.56 (d, *J* = 8.2 Hz, 1H), 7.47–7.41 (m, 3H), 6.89 (d, *J* = 8.7 Hz, 2H); ^13^C-NMR (75 MHz, DMSO-*d*_6_) δ 167.1, 159.5, 153.9, 148.9, 146.6, 143.3, 142.3, 133.8, 133.0, 133.0, 131.4, 129.4, 129.2, 128.0, 125.4, 121.5, 120.5, 119.3, 118.5, 116.6, 112.1, 99.3. HRMS (ESI-FTMS Mass (*m*/*z*): calcd for C_27_H_18_N_5_O_4_ [M + H]^+^ = 476.1353, found 476.1349.

*4-[4-[(E)-(methylhydrazono)methyl]-3-(2-oxochromen-3-yl)pyrazol-1-yl]benzoic acid* (**29**). Brownish solid; yield: 69% (267 mg). ^1^H-NMR (300 MHz, DMSO-*d*_6_): δ 8.63 (s, 1H), 8.30 (s, 1H), 7.99 (d, *J* = 8.3 Hz, 2H), 7.84-7.81 (m, 3H), 7.66 (t, *J* = 7.5 Hz, 1H), 7.48–7.36 (m, 3H), 2.69 (s, 3H), 2.50 (s, 4H), 1.86 (s, 1H); ^13^C-NMR (75 MHz, DMSO-*d*_6_) δ 169.0, 159.4, 153.8, 154.3 142.9, 139.9, 138.3, 132.5, 130.7, 129.2, 125.4, 125.3, 125.1, 121.9, 121.8, 119.5, 117.5, 116.5. HRMS (ESI-FTMS Mass (*m*/*z*): calcd for C_21_H_17_N_4_O_4_ [M + H]^+^ = 389.1244, found 389.1239.

*4-[3-(2-Oxochromen-3-yl)-4-[(E)-1-piperidyliminomethyl]pyrazol-1-yl]benzoic acid* (**30**). Yellowish solid; yield: 86% (380 mg). ^1^H-NMR (300 MHz, DMSO-*d*_6_): δ 8.75 (s, 1H), 8.29 (s, 1H), 8.05 (s, 4H), 7.83 (d, *J* = 7.6 Hz, 1H), 7.68–7.63 (m, 1H), 7.50–7.46 (m, 2H), 7.42–7.37 (m, 1H), 2.94 (br s, 4H), 1.58 (br s, 4H), 1.43 (br s, 2H); ^13^C-NMR (75 MHz, DMSO-*d*_6_) δ ^13^C-NMR (75 MHz, DMSO-*d*_6_) δ 167.1, 159.5, 153.9, 146.6, 142.8, 142.4, 132.6, 131.3, 129.2, 129.0, 127.4, 126.0, 125.1, 122.2, 121.8, 119.4, 118.4, 116.5, 51.9, 24.9, 24.0. HRMS (ESI-FTMS Mass (*m*/*z*): calcd for C_25_H_23_N_4_O_4_ [M + H]^+^ = 443.1714, found 443.1701.

*4-[4-[(E)-(Benzylhydrazono)methyl]-3-(2-oxochromen-3-yl)pyrazol-1-yl]benzoic acid* (**31**). Yellowish solid; yield: 84% (389 mg). ^1^H-NMR (300 MHz, DMSO-*d*_6_): δ 8.75 (s, 1H), 8.25 (s, 1H), 8.04 (s, 4H), 7.81 (s, 1H), 7.66 (s, 1H), 7.56 (s, 1H), 7.44 (d, *J* = 25.6 Hz, 2H), 7.22 (s, 5H), 4.18 (s, 2H); ^13^C-NMR (75 MHz, DMSO-*d*_6_) δ 167.0, 159.3, 153.9, 146.3, 143.2, 142.5, 139.6, 132.7, 131.3, 129.3, 128.8, 128.5, 128.3, 127.1, 125.9, 125.1, 121.9, 121.6, 119.3, 118.3, 116.5. HRMS (ESI-FTMS Mass (*m*/*z*): calcd for C_27_H_21_N_4_O_4_ [M + H]^+^ = 465.1557, found 465.1542.

## 4. Conclusions

We have reported the synthesis of new hydrazone derivatives of coumarin-derived pyrazoles. These new molecules are the potent growth inhibitors of Gram-positive and Gram-negative bacterial strains. We found several molecules that inhibited *A. baumannii* growth with an MIC value as low as 1.56 μg/mL. Some of them are also good inhibitors of MRSA and *B. subtilis*. Absence of toxicity in both in vitro and In vivo models are making them good candidates for further therapeutic development. 

## Figures and Tables

**Table 1:**
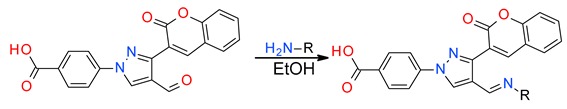
Synthesis and antimicrobial activity of coumarin-substituted hydrazone derivatives, Gram-positive bacteria: *S. aureus* ATCC 25923 (Sa) and *B. subtilis* ATCC 6623 (Bs), Gram-negative bacteria: *Escherichia coli* ATCC 25922 (Ec), *E. aerogenes* ATCC 13048 (Ea), *A. baumannii* ATCC 19606 (type strain), *Pseudomonas aeruginosa* 27833 (Pa), *Klebsiella pneumoniae* ATCC 700603 (Kp), and NA = no activity (compounds did not show any noticeable activity up to 50 μg/mL concentration). Values are the average of two closely related experimental values.

SN	Structure	Sa	Bs	Ec	Ea	Ab	Pa	Kp
**5**	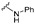	NA	NA	NA	NA	25	NA	NA
**6**	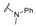	NA	>25	NA	NA	NA	NA	NA
**7**	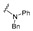	6.25	6.25	NA	NA	NA	NA	NA
**8**	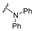	6.25	3.125	NA	NA	NA	NA	NA
**9**	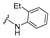	>25	12.5	NA	NA	NA	NA	NA
**10**	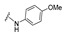	NA	NA	NA	NA	NA	NA	NA
**11**	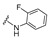	>25	>25	NA	NA	25	NA	NA
**12**	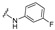	25	25	NA	NA	6.25	NA	NA
**13**	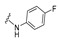	>25	25	NA	NA	25	NA	NA
**14**	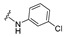	>25	>25	NA	NA	25	NA	NA
**15**	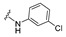	25	12.5	NA	NA	3.125	NA	NA
**16**	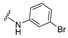	NA	NA	NA	NA	12.5	NA	NA
**17**	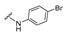	25	12.5	NA	NA	6.25	NA	NA
**18**	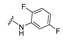	12.5	NA	NA	NA	12.5	NA	NA
**19**	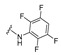	NA	NA	NA	NA	NA	NA	NA
**20**	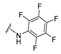	NA	NA	NA	NA	NA	NA	NA
**21**	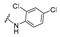	3.125	3.125	NA	NA	NA	NA	NA
**22**	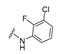	NA	NA	NA	NA	>25	NA	NA
**23**	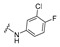	25	25	NA	NA	25	NA	NA
**24**	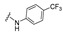	6.25	3.125	NA	NA	NA	NA	NA
**25**	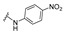	NA	NA	NA	NA	25	NA	NA
**26**	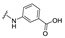	NA	NA	NA	NA	NA	NA	NA
**27**	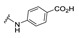	NA	NA	NA	NA	NA	NA	NA
**28**	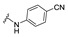	NA	NA	NA	NA	NA	NA	NA
**29**	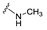	NA	NA	NA	NA	>25	NA	NA
**30**	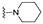	NA	NA	NA	NA	NA	NA	NA
**31**	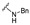	NA	NA	NA	NA	NA	NA	NA
Vancomycin		0.78						
Colistin				0.78				

**Table 2:** Antimicrobial properties of the lead molecules against *S. aureus* ATCC 33591 (Sa91), *S. aureus* ATCC 33592 (Sa92), *S. aureus* ATCC 43300 (Sa00), *S. epidermidis* 700296 (Se), *A. baumannii* ATCC BAA-1605 (Ab05) and *A. baumannii* ATCC 747 (Ab47). NA = no activity (compounds did not show any noticeable activity up to 50 μg/mL concentration).

SN	Sa91	Sa92	Sa12	Sa00	Se	Ab05	Ab47
**7**	NA	3.125	NA	3.125	NA	NA	>25
**8**	6.25	3.125	6.25	6.25	6.25	NA	NA
**11**	NA	>25	NA	>25	NA	>25	12.5
**12**	25	25	>25	25	25	25	6.25
**13**	>25	25	>25	>25	NA	12.5	12.5
**14**	NA	25	NA	>25	>25	>25	>25
**15**	12.5	12.5	12.5	12.5	12.5	3.125	3.125
**16**	NA	25	>25	25	25	12.5	12.5
**17**	12.5	12.5	NA	25	25	6.25	1.56
**18**	NA	>25	NA	NA	NA	NA	>25
**21**	NA	3.125	NA	3.125	NA	NA	NA
**22**	NA	6.25	NA	3.125	NA	NA	NA
**23**	25	25	12.5	12.5	25	25	>25
**24**	NA	6.25	6.25	6.25	6.25	NA	NA
Vancomycin						0.78	
Colistin						0.78	

**Table 3:** Calculated physicochemical properties.

Property	15	17
ilog*P*	3.07	3.26
TPSA (A^2^)	109.72	103.59

## Supplementary Materials

Supplementary Materials are available online.
