# Supplementary material for: Synthesis of Hydrazone Derivatives of 4-[4-Formyl-3-(2-oxochromen-3-yl)pyrazol-1-yl]benzoic acid as Potent Growth Inhibitors of Antibiotic-resistant Staphylococcus aureus and Acinetobacter baumannii
Source: Molecules. 2019 May 29;24(11):2051. doi: 10.3390/molecules24112051 (PMC6600224; doi:10.3390/molecules24112051)
Supplement: Supplementary file 1 [file molecules-24-02051-s001.pdf]

Synthesis of Hydrazone Derivatives of 4-[4-Formyl-3-(2-oxochromen-3-yl)pyrazol-1-yl]benzoic acid as Potent Growth Inhibitors of Antibiotic-resistant *Staphylococcus aureus* and *Acinetobacter baumannii*

Whitt,[a] J.; Duke,[a] C.; Sumlin,[a] A.; Chambers, A.; Alnufaie, R.; Gilmore,[b] D. F.; Fite, [c,d] T.; Basnakian, [c,d] A.G.; Alam*[a], M. A.

✉ Mohammad A. Alam

malam@astate.edu

[a] Department of Chemistry and Physics, College of Science and Mathematics, Arkansas State University, Jonesboro, AR 72467

[b] Department of Biological Sciences, College of Science and Mathematics, Arkansas State University, Jonesboro, AR 72467

[c] Department of Pharmacology and Toxicology, University of Arkansas for Medical Sciences, 4301 W. Markham St., Little Rock, AR 72205

[d] Central Arkansas Veterans Healthcare System, W. 7th St., Little Rock, AR 72205

Experimental methods

All the chemicals were purchased from Fisher Chemical and Oakwoochemical. Commercially available solvents were used without drying. ^{1}H and $^{13}CNMR$ spectra were obtained with a Varian Mercury-300MHz in DMSO-d_6 with TMS as internal standard. Purity of the compounds were determined by the ^{1}H and ^{13}C NMR spectroscopy. The ESI-FTMS Mass spectra were recorded Bruker ApexII-FTMS system.

Synthesis of hydrazone derivatives:[1-3] A mixture of 4-hydrazinobenzoic acid hydrochloride (**1**, 1.980 g, 10.5 mmol), 3-acetylcoumarin (1.881 g, 10 mmol), and sodium acetate (0.82 g, 10 mmol) in ethanol. Ethanol was evaporated under the reduced pressure and the hydrazone derivative was dried in vacuum. The dried material was subjected to further reaction without isolation or purification. The coumarin-derived hydrazone was dissolved in anhydrous N,N-dimethyl formamide (30 mL) and sealed by a rubber septum. The solution was cooled under ice for ~10 minutes followed by the dropwise addition of phosphorous oxychloride ($POCl_3$, 4.67 mL, 50 mmol). The reaction mixture was brought to room temperature and heated 80 ^{o}C for eight hours. After the completion of the reaction, the reaction mixture was poured onto ice and the mixture was stirred for 12 hours. The solid product was filtered and washed with water repeatedly followed by drying the product under vacuum to get the pure product in a very good overall yield.

4-[4-formyl-3-(2-oxochromen-3-yl)pyrazol-1-yl]benzoic acid (4). Whitish solid, yield: 92% (3.31 g) ^{1}H NMR (300MHz, DMSO-d_6): δ 9.93 (s, 1H), 9.35 (s, 1H), 8.47 (s, 1H), 8.12-8.05 (m, 4H), 7.87 (d, J = 6.7 Hz, 1H), 7.72-7.68 (m, 1H), 7.51 (d, J = 8.2 Hz, 1H), 7.45-7.40 (m, 1H); ^{13}C NMR (75 MHz, DMSO-d_6) δ 186.0, 167.3, 159.8, 154.0, 148.2, 143.7, 141.4, 133.5, 133.2, 131.3, 129.6, 125.4, 124.2, 120.1, 119.3, 119.2, 116.7, 115.2. HRMS (ESI-FTMS Mass (m/z): calcd for $C_{20}H_{12}N_2O_5$ $[M+H]^+$ = 361.0819, found 361.0816.

Synthesis of hydrazones: A mixture of the coumarin-derived aldehyde (360 mg, 1 mmol), hydrazine derivative (1.05 mmol) and sodium acetate (86 mg, 1. 05 mmol) in case of the hydrochloride salt of the hydrazine derivatives in anhydrous ethanol was refluxed for 8 hours. The solid product was filtered and washed with water (~20 mL) followed by washing with ethanol (~15 mL) to get the pure product, which was dried under vacuum for biological studies.

4-[3-(2-oxochromen-3-yl)-4-[(E)-(phenylhydrazono)methyl]pyrazol-1-yl]benzoic acid (5). Yellowish; yield: 82% (369 mg). ^{1}H NMR (300MHz, DMSO-d_6): δ 10.20 (s, 1H), 8.97 (s, 1H), 8.35 (s, 1H), 8.08 (s, 5H), 7.87-7.83 (m, 2H), 7.71-7.68 (m, 1H), 7.54-7.51 (m, 1H), 7.43 (t, J=6.8 Hz, 1H), 7.00-6.95 (m, 2H), 6.75 (d, J = 7.4 Hz, 2H), 6.62 (t, J = 6.3 Hz, 1H); ^{13}C NMR (75 MHz, DMSO-d_6) δ 167.1, 159.4, 153.9, 146.3, 145.6, 143.0, 142.4, 132.8, 131.4, 129.3, 129.2, 129.0, 127.2, 125.3, 122.1, 121.3, 119.3, 118.7, 118.3, 116.5, 112.0. HRMS (ESI-FTMS Mass (m/z): calcd for $C_{26}H_{19}N_4O_4$ $[M+H]^+$ = 451.1401, found 451.1395.

4-[4-[(E)-[methyl(phenyl)hydrazono]methyl]-3-(2-oxochromen-3-yl)pyrazol-1-yl]benzoic acid (6). Shiny yellow; yield: 90% (417 mg). ^{1}H NMR (300MHz, DMSO-d_6): δ 8.93 (s, 1H), 8.35 (s, 1H), 8.09 (s, 4H), 7.84-7.73 (m, 1H), 7.71-7.68 (m, 1H), 7.62 (s, 1H), 7.51-7.41 (m, 2H), 7.08-7.05 (m, 2H), 6.96-6.72 (m, 2H), 6.72 (t, J = 7.1 Hz, 1H), 3.30 (s, 3H); ^{13}C NMR (75 MHz, DMSO-d_6) δ 167.1, 159.5, 153.9, 147.5, 146.3, 142.9, 142.5, 132.6, 131.4, 129.2, 129.0, 128.8, 127.2, 125.4, 125.2, 122.6, 122.1, 120.0, 119.4, 118.3, 116.6, 114.5, 32.7. HRMS (ESI-FTMS Mass (m/z): calcd for $C_{27}H_{20}N_4O_4$ $[M+H]^+$ = 465.1557, found 465.1545.

4-[4-[(E)-[benzyl(phenyl)hydrazono]methyl]-3-(2-oxochromen-3-yl)pyrazol-1-yl]benzoic acid (7). Yellow solid; yield: 92% (496 mg). ^{1}H NMR (300MHz, DMSO-d_6): δ 8.87 (s, 1H), 8.31 (s, 1H), 8.09-8.04 (m, 4H), 7.74 (d, J = 7.3 Hz, 1H), 7.74-7.69 (m, 1H), 7.58 (s, 1H), 7.51-7.42 (m, 2H), 7.30-7.21 (m, 3H), 7.13 (d, J = 7.0 Hz, 2H), 6.98-6.96 (m, 2H), 6.87-6.83 (m, 2H), 6.71 (t, J = 6.4 Hz, 1H), 5.19 (s, 2H); ^{13}C NMR (75 MHz, DMSO-d_6) δ 167.1, 159.3, 153.8, 147.1, 146.2, 142.7, 142.4, 139.8, 136.2, 132.7, 131.4, 129.2, 129.1, 128.8, 127.8, 127.4, 126.6, 125.2, 122.8, 121.7, 120.1, 119.3, 118.3, 116.6, 113.9, 48.2. HRMS (ESI-FTMS Mass (m/z): calcd for $C_{20}H_{12}N_2O_5$ $[M+H]^+$ = 541.1870, found 541.1860.

4-[4-[(E)-(diphenylhydrazono)methyl]-3-(2-oxochromen-3-yl)pyrazol-1-yl]benzoic acid (8). Orange; yield: 91% (478 mg). ^{1}H NMR (300MHz, DMSO-d_6): δ 8.92 (s, 1H), 8.35 (s, 1H), 8.09-7.98 (m, 4H), 7.87 (d, J = 7.4 Hz, 1H), 7.75-7.70 (m, 1H), 7.52-7.43 (m, 2H), 7.27-7.22 (m, 5H), 7.16-7.09 (m, 2H), 6.93 (d, J = 7.7 Hz, 4H); ^{13}C NMR (75 MHz, DMSO-d_6) δ 167.0, 159.3, 153.8, 146.1, 143.1, 142.9, 142.4, 132.8, 131.4, 130.2, 129.3, 128.9, 128.5, 128.0, 125.3, 124.8, 122.6, 122.2, 121.0, 119.3, 118.2, 116.6. HRMS (ESI-FTMS Mass (m/z): calcd for $C_{32}H_{22}N_4O_4$ $[M+H]^+$ = 527.1714, found 527.1699.

4-[4-[(E)-[(2-ethylphenyl)hydrazono]methyl]-3-(2-oxochromen-3-yl)pyrazol-1-yl]benzoic acid (9). Yellow solid; yield: 80% (382 mg). ^{1}H NMR (300MHz, DMSO-d_6): δ 9.48 (s, 1H), 8.98 (s, 1H), 8.53 (s, 1H), 8.12-8.09 (m, 6H), 7.86 (d, J=7.6 Hz, 1H), 7.71 (t, J=8.1 Hz, 1H), 7.52 (d, J=8,2 Hz,1H), 7.44 (t, J=7.4 Hz,1H), 6.96 (t, J=7.2 Hz, 2H), 6.69-6.59 (m, 2H), 1.11 (t, J=7.3 Hz, 3H); ^{13}C NMR (75 MHz, DMSO-d_6) δ 167.1, 159.4, 153.9, 146.4, 143.1, 142.8, 142.4, 132.8, 131.4, 130.3, 129.3, 128.6, 127.1, 126.5, 125.3, 122.2, 121.5, 119.3, 119.0, 118.3, 116.6, 112.3. HRMS (ESI-FTMS Mass (m/z): calcd for $C_{28}H_{22}N_4O_4$ $[M+H]^+$ = 479.1714, found 479.1700.

4-[4-[(E)-[(4-methoxyphenyl)hydrazono]methyl]-3-(2-oxochromen-3-yl)pyrazol-1-yl]benzoic acid (10). Orange solid; yield: 83% (398 mg). ^{1}H NMR (300MHz, DMSO-d_6): δ 9.96 (s, 1H), 8.93 (s,1H), 8.34 (s, 1H), 8.08 (s, 4H), 7.85 (d, J=7.5 Hz, 1H), 7.77-7.69 (m, 2H), 7.53 (d, J=8.2 Hz, 1H), 7.44 (t, J=7.4 Hz,1H), 6.72-6.69 (m, 2H), 6.61-6.58 (m,1H); ^{13}C NMR (75 MHz, DMSO-d_6) δ 167.1, 159.4, 153.9, 152.7, 146.2, 143.0, 142.5, 139.7, 132.7, 131.4, 129.3, 128.9, 127.9, 126.8, 125.3, 122.2, 121.5, 119.4, 118.3, 116.6, 114.7, 113.0. HRMS (ESI-FTMS Mass (m/z): calcd for $C_{27}H_{20}N_4O_5$ $[M+H]^+$ = 481.1506, found 481.1495.

4-[4-[(E)-[(2-fluorophenyl)hydrazono]methyl]-3-(2-oxochromen-3-yl)pyrazol-1-yl]benzoic acid (11). Orange solid; yield: 80% (374 mg). ^{1}H NMR (300MHz, DMSO-d_6): δ 10.12 (s, 1H), 9.01 (s, 1H), 8.35 (s, 1H), 8.11-8.09 (m, 5H), 7.86 (d, J=7.6 Hz, 1H), 7.71 (t, J=7.7 Hz, 1H), 7.53 (d, J=8.3 Hz, 1H), 7.44 (t, J=7.3 Hz, 1H) 7.09-6.96 (m, 2H), 6.71-6.66 (m, 1H); ^{13}C NMR (75 MHz, DMSO-d_6) δ 167.1, 159.4, 153.9, 150.9, 147.7, 146.4, 143.1, 142.3, 133.7 (d, J=9.8 Hz), 132.4 (d, J=64.2 Hz), 131.4, 129.3, 127.6, 125.3, 124.8, 122.1, 121.0, 119.3, 118.4, 116.5, 115.3 (d, J=16.9 Hz), 113.8. HRMS (ESI-FTMS Mass (m/z): calcd for $C_{26}H_{17}N_4O_4F$ $[M+H]^+$ = 469.1307, found 469.1292.

4-[4-[(E)-[(3-fluorophenyl)hydrazono]methyl]-3-(2-oxochromen-3-yl)pyrazol-1-yl]benzoic acid (12). Off orange; yield: 78% (365 mg). ^{1}H NMR (300MHz, DMSO-d_6): δ 10.42 (s, 1H), 9.02 (s, 1H), 8.36 (s, 1H), 8.09 (s, 4H), 7.89-7.84 (m, 2H), 7.69 (t, J=7.8 Hz, 1H), 7.51-7.39 (m, 2H), 7.07-6.99 (m, 1H), 6.59-6.54 (m, 2H), 6.40 (t, J=7.9 Hz, 1H); ^{13}C NMR (75 MHz, DMSO-d_6) δ 167.1, 165.3, 162.1, 159.4, 153.8, 147.6 (d, J=11.0 Hz), 146.4, 142.8 (d, J=54.5 Hz), 132.8, 131.4, 130.8 (d, J=9.9 Hz), 130.4, 129.3, 129.0, 127.6, 125.3, 121.9,

120.9, 119.3, 118.4, 116.6, 108.2, 104.8 (d, J=21.3 Hz), 98.5 (d, J=26.3 Hz). HRMS (ESI-FTMS Mass (m/z): calcd for $C_{26}H_{17}N_4O_4F$ $[M+H]^+$ = 469.1307, found 469.1294.

4-[4-[(E)-[(4-fluorophenyl)hydrazono]methyl]-3-(2-oxochromen-3-yl)pyrazol-1-yl]benzoic acid (13). Yellow solid; yield: 75% (355 mg). ^{1}H NMR (300MHz, DMSO-d_6): δ 10.18 (s, 1H), 8.97 (s, 1H), 8.35 (s, 1H), 8.08 (s, 4H), 7.87-7.82 (m, 2H), 7.71 (t, J=7.6 Hz, 1H), 7.54 (d, J=8.3 Hz, 1H), 7.44 (t, J=7.4 Hz, 1H), 6.84-6.78 (m, 4H); ^{13}C NMR (75 MHz, DMSO-d_6) δ 167.1, 159.4, 153.8, 146.3, 143.1, 142.4, 142.3, 132.8, 131.4, 129.3, 129.0, 127.1, 125.3, 122.0, 121.2, 119.3, 118.4, 116.6, 115.7 (d, J=22.2 Hz), 112.9 (d, J=7.2 Hz). HRMS (ESI-FTMS Mass (m/z): calcd for $C_{26}H_{17}N_4O_4F$ $[M+H]^+$ = 469.1307, found 469.1294.

4-[4-[(E)-[(3-chlorophenyl)hydrazono]methyl]-3-(2-oxochromen-3-yl)pyrazol-1-yl]benzoic acid (14). Brown solid; yield: 81% (382 mg). ^{1}H NMR (300MHz, DMSO-d_6): δ 10.57 (s, 1H), 9.02 (s, 1H), 8.36 (s, 1H), 8.08 (s, 4H), 7.90 (s, 1H), 7.86 (d, J = 7.5 Hz, 1H), 7.71-7.66 (m, 1H), 7.50 (d, J = 8.2 Hz, 1H), 7.42 (t, J = 7.4 Hz, 1H), 7.02 (t, J = 7.9 Hz, 1H), 6.79 (s, 1H), 6.67 (d, J = 8.2 Hz, 1H), 6.63 (d, J = 7.7 Hz, 1H); ^{13}C NMR (75 MHz, DMSO-d_6) δ 167.1, 159.4, 153.9, 147.0, 146.3, 143.1, 142.3, 134.2, 132.7, 131.4, 130.8, 130.6, 129.3, 129.2, 127.8, 125.3, 122.0, 120.9, 119.3, 118.4, 118.1, 116.8, 111.1, 110.8. HRMS (ESI-FTMS Mass (m/z): calcd for $C_{26}H_{17}N_4O_4Cl$ $[M+H]^+$ = 469.1307, found 469.1296.

4-[4-[(E)-[(4-chlorophenyl)hydrazono]methyl]-3-(2-oxochromen-3-yl)pyrazol-1-yl]benzoic acid (15). Orange solid; yield: 77% (372 mg). ^{1}H NMR (300MHz, DMSO-d_6): δ 13.10 (br s, 1H), 10.35 (s, 1H), 9.00 (s, 1H), 8.37 (s,1H), 8.09 (s, 4H), 7.87-7.84 (m, 2H), 7.72 (t, J = 7.5 Hz, 1H), 7.54 (d, J = 8.2 Hz, 1H), 7.44 (t, J = 7.4 Hz, 1H), 7.02 (d, J = 8.7 Hz, 2H), 6.78 (d, J = 8.7 Hz, 2H); ^{13}C NMR (75 MHz, DMSO-d_6) δ 167.0, 159.4, 153.9, 146.4, 144.5, 143.2, 142.4, 132.9, 131.4, 130.1, 129.3, 129.05, 129.01, 127.4, 125.3, 121.94, 121.90, 121.0, 119.3,118.4, 116.6, 113.4. HRMS (ESI-FTMS Mass (m/z): calcd for $C_{26}H_{17}N_4O_4Cl$ $[M+H]^+$ = 469.1307, found 469.1292, Yield = 95%.

4-[4-[(E)-[(3-bromo)hydrazono]methyl]-3-(2-oxochromen-3-yl)pyrazol-1-yl]benzoic acid (16). Brown solid; yield: 83% (438 mg). ^{1}H NMR (300MHz, DMSO-d_6): δ 13.12 (br s, 1H), 10.40 (s, 1H), 9.03 (s, 1H), 8.37 (s, 1H), 8.09 (s, 4H), 7.87-7.84 (m, 2H), 7.68 (t, J = 7.7 Hz, 1H), 7.52 (d, J = 8.2 Hz, 1H), 7.42 (t, J = 7.4 Hz, 1H), 7.00-

6.95 (m, 2H), 6.78 (d, J = 7.8 Hz, 1H), 6.72 (d, J = 8.1 Hz, 1H); ^{13}C NMR (75 MHz, DMSO-d_6) δ 167.0, 159.4, 153.9, 147.1, 146.4, 143.2, 142.4, 132.7, 131.4, 131.1, 130.8, 129.3, 129.0, 127.8, 125.3, 122.9, 121.9, 121.0, 120.8, 119.3, 118.4, 116.8, 114.0, 111.2. HRMS (ESI-FTMS Mass (m/z): calcd for $C_{26}H_{17}N_4O_4Br$ $[M+H]^+$ = 531.0487, found 531.0473.

4-[4-[(E)-[(4-bromophenyl)hydrazono]methyl]-3-(2-oxochromen-3-yl)pyrazol-1-yl]benzoic acid (17). Yellow solid; yield: 81% (427 mg). ^{1}H NMR (300MHz, DMSO-d_6): δ 10.35 (s, 1H), 9.00 (s, 1H), 8.37 (s, 1H), 8.09 (s, 4H), 7.87-7.85 (m, 2H), 7.72 (t, J=7.1 Hz, 1H), 7.55 (d, J=8.2 Hz, 1H), 7.44 (t, J=7.4 Hz, 1H), 7.14 (d, J=8.7 Hz, 2H), 6.73 (d, J=8.8 Hz, 2H); ^{13}C NMR (75 MHz, DMSO-d_6) δ 167.0, 159.4, 153.9, 146.4, 144.9, 143.2, 142.4, 132.9, 131.8, 131.4, 130.2, 129.3, 129.0, 127.4, 125.3, 121.8, 121.0, 119.3, 118.4, 116.6, 113.9, 109.5. HRMS (ESI-FTMS Mass (m/z): calcd for $C_{26}H_{17}N_4O_4Br$ $[M+H]^+$ = 531.0487, found 531.0471

4-[4-[(E)-[(2,5-difluorophenyl)hydrazono]methyl]-3-(2-oxochromen-3-yl)pyrazol-1-yl]benzoic acid (18). Reddish solid; yield: 78% (377 mg). ^{1}H NMR (300MHz, DMSO-d_6): δ 10.36 (s,1H), 9.09 (s, 1H), 8.37 (s, 1H), 8.13-8.09 (m, 5H), 7.85 (d, J=7.2 Hz, 1H), 7.68 (t, J=7.2 Hz, 1H), 7.49-7.41 (m, 2H), 7.10 (br s, 1H), 6.77 (br s, 1H), 6.40 (br s, 1H); ^{13}C NMR (75 MHz, DMSO-d_6) δ 167.0, 159.5 (d, J=235.7 Hz), 159.4, 153.8, 147.0, 146.5, 143.9, 143.3, 142.4, 135.1 (t, J=11.8 Hz), 133.3, 132.9, 131.4, 129.3, 129.0, 128.0, 125.3, 121.7, 120.7, 119.2, 118.4, 116.6-116.0 (m), 103.8 (d, J=24.0 Hz), 100.2 (d, J=26.0 Hz). HRMS (ESI-FTMS Mass (m/z): calcd for $C_{26}H_{17}N_4O_4F_2$ $[M+H]^+$ = 487.1212, found 487.1199.

4-[3-(2-oxochromen-3-yl)-4-[(E)-[(2,3,5,6-tetrafluorophenyl)hydrazono]methyl]pyrazol-1-yl]benzoic acid (19). Yellow solid; yield: 89% (464 mg). ^{1}H NMR (300MHz, DMSO-d_6): δ 10.28 (s, 1H), 8.94 (s, 1H), 8.31 (s, 1H), 8.17 (s, 1H), 8.05 (s, 3H), 7.82 (d, J=7.5 Hz, 1H), 7.67 (t, J=7.4 Hz, 1H), 7.46-7.37 (m, 2H), 7.14-7.02 (m, 1H); ^{13}C NMR (75 MHz, DMSO-d_6) δ 167.0, 159.4, 154.0, 148.1, 142.1, 142.3, 135.1, 132.6, 131.4, 129.3-129.2 (m), 128.1, 125.1, 121.7, 120.2, 119.3, 118.5, 116.4, 95.2 (t, J=23.5 Hz). HRMS (ESI-FTMS Mass (m/z): calcd for $C_{26}H_{15}N_4O_4F_4$ $[M+H]^+$ = 523.1024, found 523.1008.

4-[3-(2-oxochromen-3-yl)-4-[(E)-[(2,3,4,5,6-pentafluorophenyl)hydrazono]methyl]pyrazol-1-yl]benzoic acid (20). Yellow solid; yield: 90% (486 mg). ^{1}H NMR (300MHz, DMSO-d_6): δ 10.12 (s, 1H), 8.92 (s, 1H), 8.30

(s, 1H), 8.13-8.07 (m, 5H), 7.82 (d, J=7.6 Hz, 1H), 7.67 (t, J=7.6 Hz, 1H), 7.47-7.37 (m, 2H); ^{13}C NMR (75 MHz, DMSO-d_6) δ 167.1, 159.4, 154.0, 146.6, 143.1, 142.3, 135.1, 132.6, 131.3, 129.5, 129.2, 128.0, 125.1, 121.6, 119.6, 119.3, 118.5, 116. HRMS (ESI-FTMS Mass (m/z): calcd for $C_{26}H_{14}N_4O_4F_5$ $[M+H]^+$ = 541.0930, found 541.0919.

4-[4-[(E)-[(2,4-dichlorophenyl)hydrazono]methyl]-3-(2-oxochromen-3-yl)pyrazol-1-yl]benzoic acid (21). Brownish solid; yield: 85% (440 mg). ^{1}H NMR (300MHz, DMSO-d_6): δ 9.94 (s, 1H), 9.05 (s, 1H), 8.37 (s, 1H), 8.27 (s, 1H), 8.09 (s, 4H), 7.86 (d, J=7.7 Hz, 1H), 7.72 (t, J=7.4 Hz, 1H), 7.54 (d, J=8.1 Hz, 1H), 7.47-7.39 (m, 2H), 7.11 (d, J=8.8 Hz, 1H), 6.89 (d, J=8.8 Hz, 1H); ^{13}C NMR (75 MHz, DMSO-d_6) δ 167.1, 159.4, 153.9, 146.6, 143.4, 142.3, 140.9 133.9, 132.9, 131.4, 129.4, 129.0, 127.8, 125.4, 122.2, 121.7, 120.7, 119.3, 118.5, 116.8, 116.6, 114.9. HRMS (ESI-FTMS Mass (m/z): calcd for $C_{26}H_{17}N_4O_4Cl_2$ $[M+H]^+$ = 519.0621, found 519.0615.

4-[4-[(E)-[(3-chloro-2-fluoro-phenyl)hydrazono]methyl]-3-(2-oxochromen-3-yl)pyrazol-1-yl]benzoic acid (22). Orange solid; yield: 72% (361 mg). ^{1}H NMR (300MHz, DMSO-d_6): δ 10.37 (s, 1H), 9.04 (s, 1H), 8.35 (s, 1H), 8.14 (s, 1H), 8.09 (s, 4H), 7.86 (d, J = 7.3 Hz, 1H), 7.73-7.68 (m, 1H), 7.53 (d, J = 8.1 Hz, 1H), 7.45-7.41 (m, 1H), 6.98 (m, 1H), 6.78-6.67 (m, 2H); ^{13}C NMR (75 MHz, DMSO-d_6) δ 167.1, 159.4, 153.8, 146.5, 144.68 ($^1J_{C-F}$ = 239.8 Hz), 143.2, 142.4, 135.3, 135.1, 133.1 ($^2J_{C-F}$ = 22.3 Hz), 131.4, 129.3 ($^2J_{C-F}$ = 22.7 Hz), 127.9, 125.3, 121.8, 120.7, 120.0, 119.8, 119.3, 118.7, 118.4, 116.5, 112.5. HRMS (ESI-FTMS Mass (m/z): calcd for $C_{26}H_{17}N_4O_4ClF$ $[M+H]^+$ = 503.0917, found 503.0903.

4-[4-[(E)-[(3-chloro-4-fluoro-phenyl)hydrazono]methyl]-3-(2-oxochromen-3-yl)pyrazol-1-yl]benzoic acid (23). Yellow solid; yield: 76% (381 mg). ^{1}H NMR (300MHz, DMSO-d_6): δ 10.35 (s, 1H), 9.01 (s, 1H), 8.36 (s, 1H), 8.08-8.07 (m, 4H), 7.86-7.84 (m, 2H), 7.69 (t, J=7.5 Hz, 1H), 7.51 (d, J=8.2 Hz, 1H), 7.42 (t, J=7.4 Hz, 1H), 7.09 (t, J=8.9 Hz, 1H), 6.88-6.87 (m, 1H), 6.71-6.68 (m, 1H); ^{13}C NMR (75 MHz, DMSO-d_6) δ 167.1, 159.4, 153.9, 152.4, 149.2, 146.3, 143.3, 142.3, 132.8, 131.4, 130.7, 129.4, 129.3, 127.7, 125.3, 121.9, 120.8, 120.2 (d, J=18.3 Hz), 119.3, 118.4, 117.4 (d, J=21.6 Hz), 116.8, 112.4, 111.8 (d, J=6.4 Hz). HRMS (ESI-FTMS Mass (m/z): calcd for $C_{26}H_{17}N_4O_4ClF$ $[M+H]^+$ = 503.0917, found 503.0910.

4-[3-(2-oxochromen-3-yl)-4-[(E)-[[4-(trifluoromethyl)phenyl]hydrazono]methyl]pyrazol-1-yl]benzoic acid (24). Yellow solid; yield: 81% (419 mg). ^{1}H NMR (300MHz, DMSO-d$_6$): δ 10.68 (s, 1H), 9.03 (s, 1H), 8.37 (s, 1H), 8.09 (s, 4H), 7.92 (s, 1H), 7.87 (d, J = 7.5 Hz, 1H), 7.74-7.69 (m, 1H), 7.54 (d, J = 8.1 Hz, 1H), 7.46-7.41 (m, 1H), 7.31 (d, J = 8.3 Hz, 2H), 6.91 (d, J = 8.4 Hz, 2H); ^{13}C NMR (75 MHz, DMSO-d$_6$) δ 167.1, 159.5, 153.9, 148.5, 146.5, 143.3, 142.4, 132.9, 131.9, 131.4, 129.4, 129.1, 127.7, 127.1, 126.6, 125.3, 121.7, 120.7, 119.3, 118.2, 116.6, 111.6. HRMS (ESI-FTMS Mass (m/z): calcd for $C_{27}H_{18}N_4O_4F_3$ $[M+H]^+$ = 519.1275, found 519.1262.

4-[4-[(E)-[(4-nitrophenyl)hydrazono]methyl]-3-(2-oxochromen-3-yl)pyrazol-1-yl]benzoic acid (25). Reddish; yield: 89% (440 mg). ^{1}H NMR (300MHz, DMSO-d$_6$): δ 11.23 (s, 1H), 9.13 (s, 1H), 8.42 (s, 1H), 8.11 (s, 4H), 8.04 (s, 1H), 7.96-7.87 (m, 3H), 7.74 (t, J=7.3 Hz, 1H), 7.56 (d, J=8.3 Hz, 1H), 7.46 (t, J=7.4 Hz, 1H), 6.91 (d, J=9.2, 2H); ^{13}C NMR (75 MHz, DMSO-d$_6$) δ 167.0, 159.5, 153.9, 150.8, 146.7, 143.4, 142.3, 138.4, 134.9, 133.0, 131.4, 129.4, 129.2, 128.3, 126.3, 125.4, 121.4, 120.2, 119.3, 118.5, 116.6, 111.2. HRMS (ESI-FTMS Mass (m/z): calcd for $C_{26}H_{18}N_5O_6$ $[M+H]^+$ = 496.1252, found 496.1240.

4-[(2E)-2-[[1-(4-carboxyphenyl)-3-(2-oxochromen-3-yl)pyrazol-4-yl]methylene]hydrazino]benzoic acid (26). Brownish solid; yield: 83% (410 mg). ^{1}H NMR (300MHz, DMSO-d$_6$): δ 10.39 (s, 1H) 8.99 (s, 1H), 8.38 (s, 1H), 8.09 (s, 4H), 7.86-7.85 (m, 2H), 7.72-7.68 (m, 1H), 7.52 (d, J = 8.2 Hz, 1H), 7.45-7.42 (m, 2H), 7.24 (d, J = 7.3 Hz, 1H), 7.10-6.99 (m, 2H); ^{13}C NMR (75 MHz, DMSO-d$_6$) δ 167.9, 167.1, 159.5, 153.9, 146.4, 145.8, 143.3, 142.4, 132.9, 132.0, 131.4, 130.3, 129.4, 129.3, 129.2, 127.3, 125.3, 121.7, 120.9, 119.7, 119.3, 118.4, 116.6, 115.9, 112.9. HRMS (ESI-FTMS Mass (m/z): calcd for $C_{27}H_{19}N_4O_6$ $[M+H]^+$ = 495.1299, found 495.1284.

4-[(2E)-2-[[1-(4-carboxyphenyl)-3-(2-oxochromen-3-yl)pyrazol-4-yl]methylene]hydrazino]benzoic acid (27). Orange solid; yield: 82% (405 mg). ^{1}H NMR (300MHz, DMSO-d$_6$): δ 10.73 (s, 1H), 8.97 (s, 1H), 8.37 (s, 1H), 8.08 (d, J=8.5 Hz, 2H), 8.01 (d, J=9.0 Hz, 3H), 7.87 (d, J=7.1 Hz, 1H), 7.72 (t, J=8.1 Hz, 1H), 7.62-7.52 (m, 3H), 7.44 (t, J=7.5 Hz, 1H), 6.81 (d, J=8.6 Hz, 2H); ^{13}C NMR (75 MHz, DMSO-d$_6$) δ 168.3, 167.9, 159.5, 153.9, 149.0, 146.0, 143.1, 141.0, 134.3, 132.8, 131.3, 131.1, 129.3, 127.6, 125.3, 122.1, 121.0, 120.5, 119.4, 118.0, 116.5, 111.1. HRMS (ESI-FTMS Mass (m/z): calcd for $C_{27}H_{19}N_4O_6$ $[M+H]^+$ = 495.1299, found 495.1290.

4-[(2E)-2-[[1-(4-cyanophenyl)-3-(2-oxochromen-3-yl)pyrazol-4-yl]methylene]hydrazino]benzoic acid (28). Off yellow solid; yield: 78% (370 mg). ^{1}H NMR (300MHz, DMSO-d_6): δ 10.87 (s, 1H), 9.07 (s, 1H), 8.39 (s, 1H), 8.12-8.09 (m, 4H), 7.95 (s, 1H), 7.87 (d, J = 7.5 Hz, 1H), 7.75-7.70 (m, 1H), 7.56 (d, J = 8.2 Hz, 1H), 7.47-7.41 (m, 3H), 6.89 (d, J = 8.7 Hz, 2H); ^{13}C NMR (75 MHz, DMSO-d_6) δ 167.1, 159.5, 153.9, 148.9, 146.6, 143.3, 142.3, 133.8, 133.0, 133.0, 131.4, 129.4, 129.2, 128.0, 125.4, 121.5, 120.5, 119.3, 118.5, 116.6, 112.1, 99.3. HRMS (ESI-FTMS Mass (m/z): calcd for $C_{27}H_{18}N_5O_4$ $[M+H]^+$ = 476.1353, found 476.1349.

4-[4-[(E)-(methylhydrazono)methyl]-3-(2-oxochromen-3-yl)pyrazol-1-yl]benzoic acid (29). Brownish solid; yield: 69% (267 mg). ^{1}H NMR (300MHz, DMSO-d_6): δ 8.63 (s, 1H), 8.30 (s, 1H), 7.99 (d, J=8.3 Hz, 2H), 7.84-7.81 (m, 3H), 7.66 (t, J=7.5 Hz, 1H), 7.48-7.36 (m, 3H), 2.69 (s, 3H), 2.50 (s, 4H), 1.86 (s, 1H); ^{13}C NMR (75 MHz, DMSO-d_6) δ 169.0, 159.4, 153.8, 154.3 142.9, 139.9, 138.3, 132.5, 130.7, 129.2, 125.4, 125.3, 125.1, 121.9, 121.8, 119.5, 117.5, 116.5. HRMS (ESI-FTMS Mass (m/z): calcd for $C_{21}H_{17}N_4O_4$ $[M+H]^+$ = 389.1244, found 389.1239.

4-[3-(2-oxochromen-3-yl)-4-[(E)-1-piperidyliminomethyl]pyrazol-1-yl]benzoic acid (30). Yellowish solid; yield: 86% (380 mg). ^{1}H NMR (300MHz, DMSO-d_6): δ 8.75 (s, 1H), 8.29 (s, 1H), 8.05 (s, 4H), 7.83 (d, J = 7.6 Hz, 1H), 7.68-7.63 (m, 1H), 7.50-7.46 (m, 2H), 7.42-7.37 (m, 1H), 2.94 (br s, 4H), 1.58 (br s, 4H), 1.43 (br s, 2H); ^{13}C NMR (75 MHz, DMSO-d_6) δ ^{13}C NMR (75 MHz, DMSO-d_6) δ 167.1, 159.5, 153.9, 146.6, 142.8, 142.4, 132.6, 131.3, 129.2, 129.0, 127.4, 126.0, 125.1, 122.2, 121.8, 119.4, 118.4, 116.5, 51.9, 24.9, 24.0. HRMS (ESI-FTMS Mass (m/z): calcd for $C_{25}H_{23}N_4O_4$ $[M+H]^+$ = 443.1714, found 443.1701.

4-[4-[(E)-(benzylhydrazono)methyl]-3-(2-oxochromen-3-yl)pyrazol-1-yl]benzoic acid (31). Yellowish solid; yield: 84% (389 mg). ^{1}H NMR (300MHz, DMSO-d_6): δ 8.75 (s, 1H), 8.25 (s, 1H), 8.04 (s, 4H), 7.81 (s, 1H), 7.66 (s, 1H), 7.56 (s, 1H), 7.44 (d, J=25.6 Hz, 2H), 7.22 (s, 5H), 4.18 (s, 2H); ^{13}C NMR (75 MHz, DMSO-d_6) δ 167.0, 159.3, 153.9, 146.3, 143.2, 142.5, 139.6, 132.7, 131.3, 129.3, 128.8, 128.5, 128.3, 127.1, 125.9, 125.1, 121.9, 121.6, 119.3, 118.3, 116.5. HRMS (ESI-FTMS Mass (m/z): calcd for $C_{27}H_{21}N_4O_4$ $[M+H]^+$ = 465.1557, found 465.1542.

MIC studies:[2-3] The Minimum Inhibitory Concentration (MIC) of the synthesized compounds was determined by a broth microdilution plate-based technique as per Clinical and Laboratory Standards Institute (CLSI) procedures for antimicrobial susceptibility testing of aerobic bacteria. In brief, bacteria were streaked onto Tryptic Soy Agar (TSA) or TSA with 5% sheep blood and incubated at 35 ℃ overnight. Colonies were suspended in sterile saline (0.9% NaCl) to match a 0.5 MacFarland standard then diluted 1:100 with Mueller Hinton broth to an estimated 1 x 10^6 CFU/ml concentration. Compounds to be tested were dissolved in DMSO to a 2 mg/ml concentration and diluted serially 1:2 in microplates such that after the addition of bacteria in broth the final concentrations began at 50 µg/ml. Inhibition of bacterial growth was determined using resazurin as a marker for cell viability.[4] Resazurin was added with bacteria to 8 µg/ml final concentration, and plates were incubated at 35 ℃ for 20-24 hours and read visually. Negative controls (bacteria without inhibitors) and positive controls (bacteria plus a serially diluted antibiotic) were included on every plate. The MIC was determined as the lowest concentration where there was no color change of resazurin from purple to red (evidence of bacterial growth).

Cytotoxicity studies: In vitro toxicity of the active compounds were carried out according to our recently reported modified procedure.[2-3] HEK 293 cells were counted using a Countess automated cell counter and 5000 cells/well were plated in black 96 well plates. Compounds were added in triplicate at 50 µg/mL and 25 µg/mL concentrations and incubated for 24 hours followed by adding the 5% resazurin solution for a final volume of 200 µL in each well. Resazurin containing plates were incubated for four and six hours before the taking the readings for cell viability. Excitation and emission for fluorescence were measured at 544nm and 590nm respectively using a BMG Labtech Fluostar Optima plate reader.

In vivo toxicity assessment. All animal experiments were performed at the Central Arkansas Veterans Healthcare System (John L. McClellan Memorial Veterans Hospital in Little Rock, AR) and have been approved by the Institutional Animal Care and Use Committee. CD1 male mice (8 weeks old, 33-37 g) were purchased from Charles River Laboratories (Wilmington, MA). CM-Cl was freshly dissolved in 0.9% saline, sterilized by ultrafiltration, and injected intraperitoneally (IP) in mice at two doses of 20 or 50 mg/kg (n=5 per dose). The two control groups (n=5/group) were untreated of administered with the vehicle (saline). The mice were euthanized 24 hours after the injection, and blood was collected by cardiac puncture. Toxicity was assessed by measuring 14

blood markers of various organ function available in the Comprehensive Diagnosis Kit (Abaxis, Union City, CA) and using VetScan VS2 instrument (Abaxis). The markers included: alanine aminotransferase (ALT), albumin (ALB), alkaline phosphatase (ALP), amylase (AMY), calcium (CA), creatinine (CRE), globulin (GLOB), glucose (GLU), phosphorus (PHOS), potassium (K^+), sodium (Na^+), total bilirubin (TBIL), total protein (TP), and blood urea nitrogen (BUN) measured by VetScan VS2 (Abaxis, Union City, CA). Our criteria for toxicity included: measurements being beyond the normal ranges, plus statistically significantly different from the untreated and vehicle (saline) controls.

4-[4-formyl-3-(2-oxochromen-3-yl)pyrazol-1-yl]benzoic acid (4)

^{1}H NMR spectrum

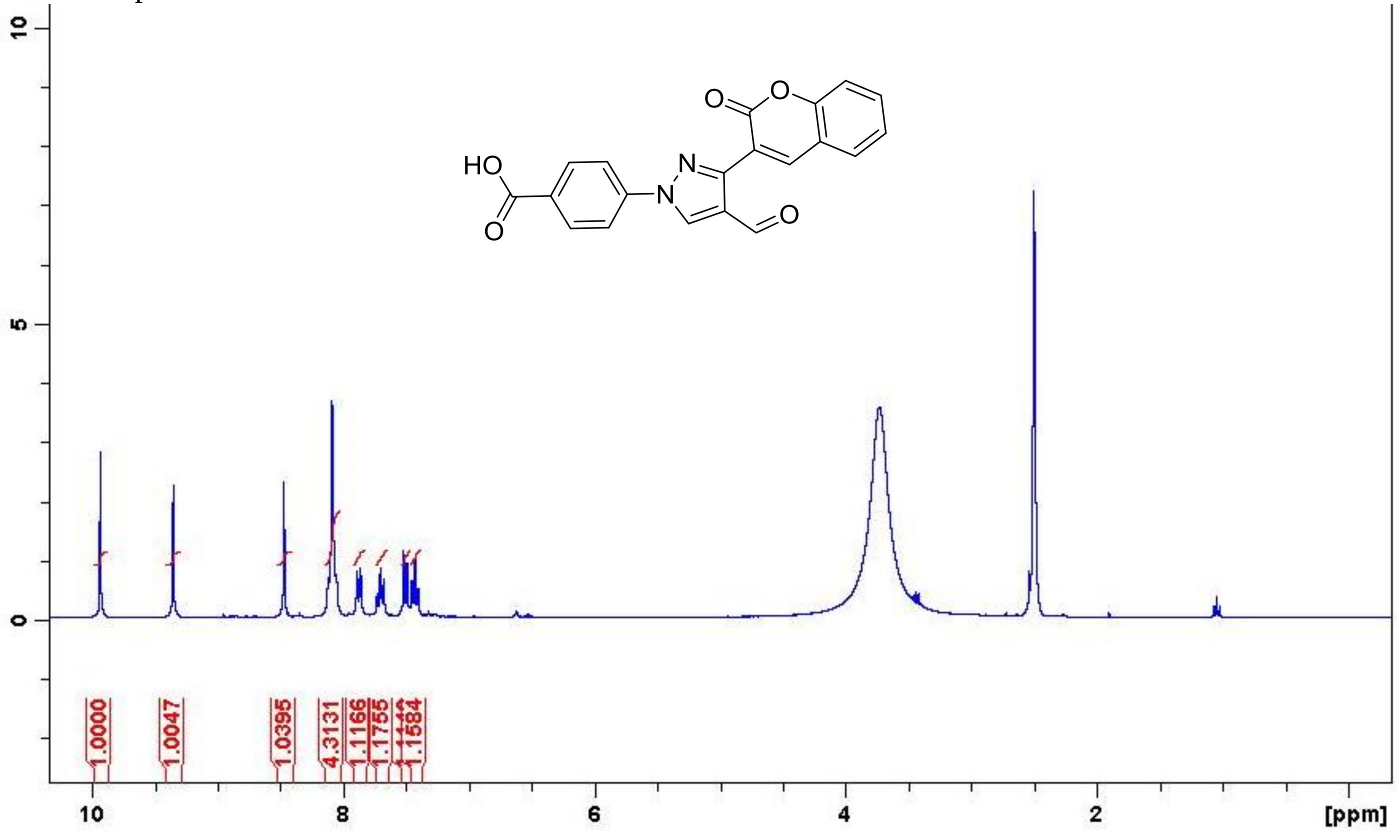

^{13}C NMR spectrum of compound **4**

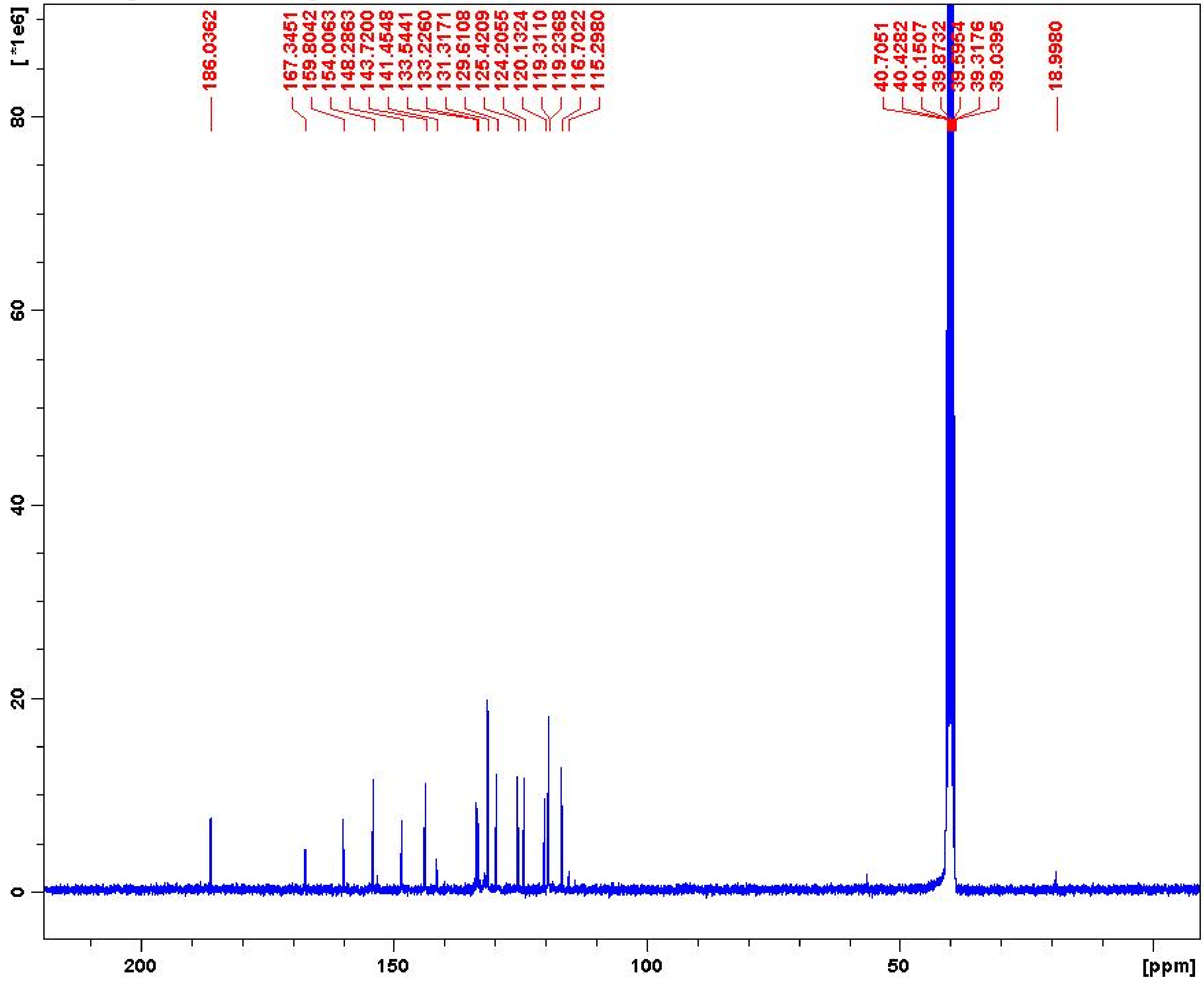

4-[3-(2-oxochromen-3-yl)-4-[(E)-(phenylhydrazono)methyl]pyrazol-1-yl]benzoic acid (5)

^{1}H NMR spectrum

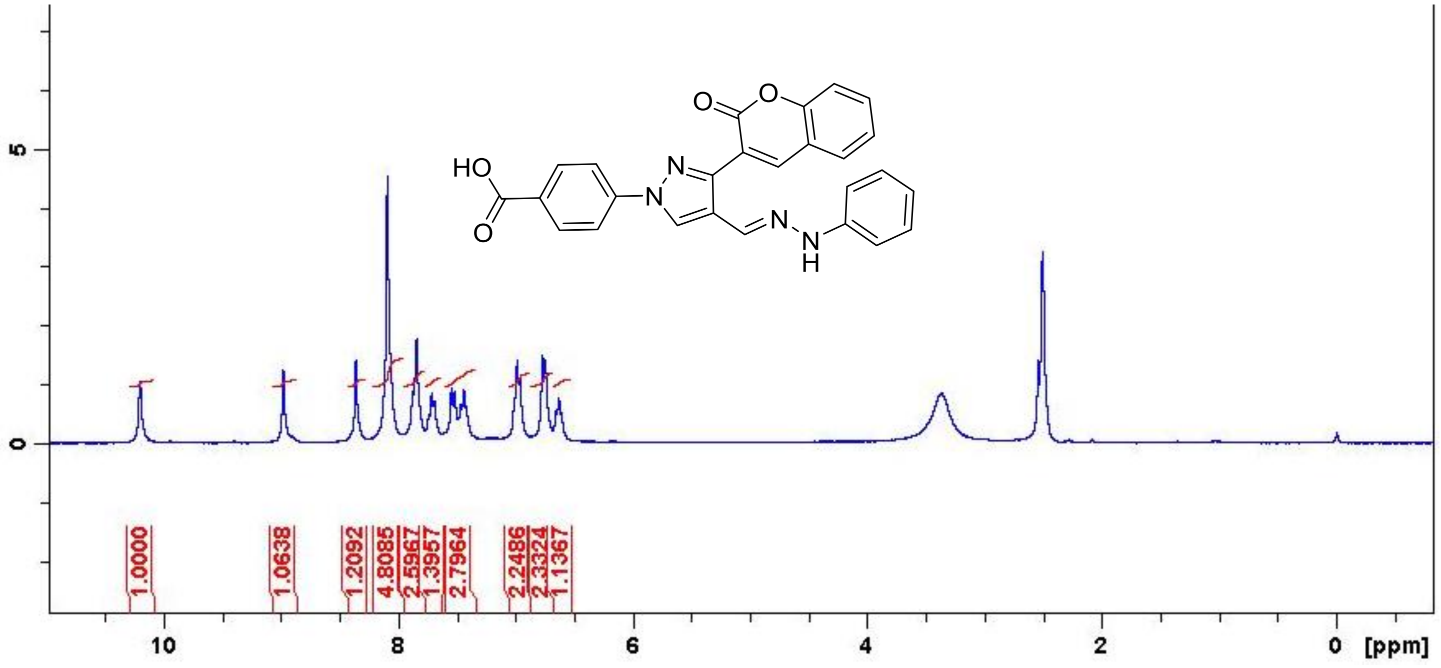

^{13}C NMR spectrum of compound **5**

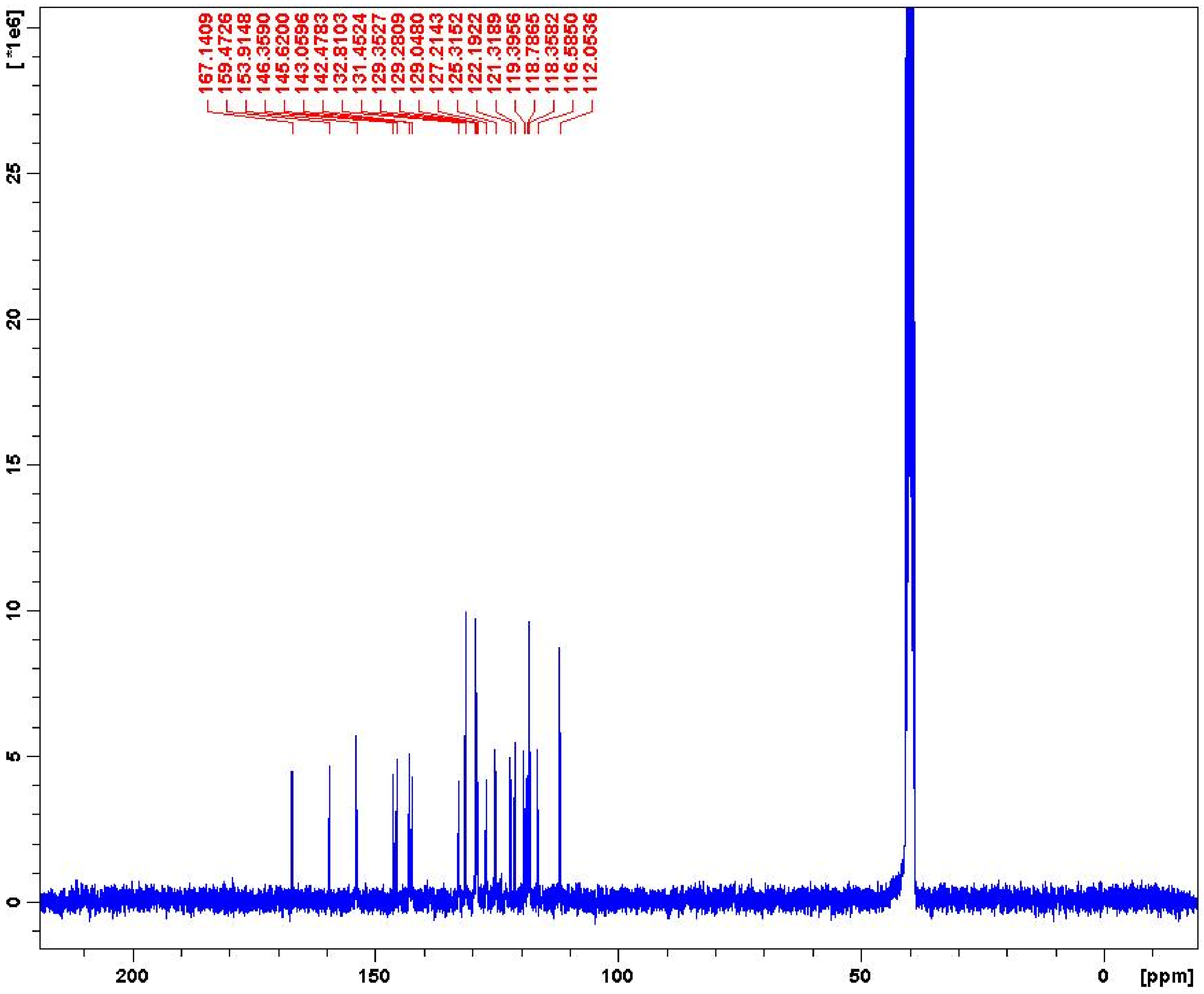

167.1409
159.4726
153.9148
146.3590
145.6200
143.0596
142.4783
132.8103
131.4524
129.3527
129.2809
129.0480
127.2143
125.3152
122.1922
121.3189
119.3956
118.7865
118.3582
116.5850
112.0536
[*1e6]
25
20
15
10
5
0
200
150
100
50
0
[ppm]

4-[4-[(E)-[methyl(phenyl)hydrazono]methyl]-3-(2-oxochromen-3-yl)pyrazol-1-yl]benzoic acid (6)

^{1}H NMR spectrum

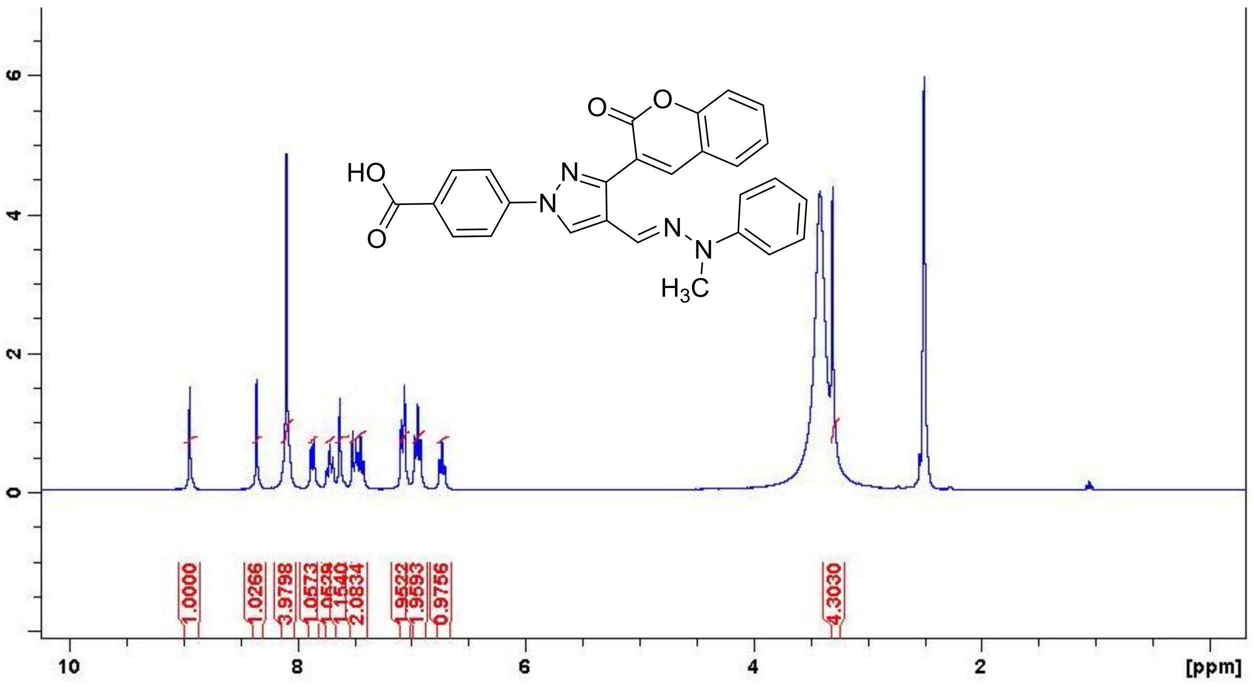

^{13}C NMR spectrum of compound **6**

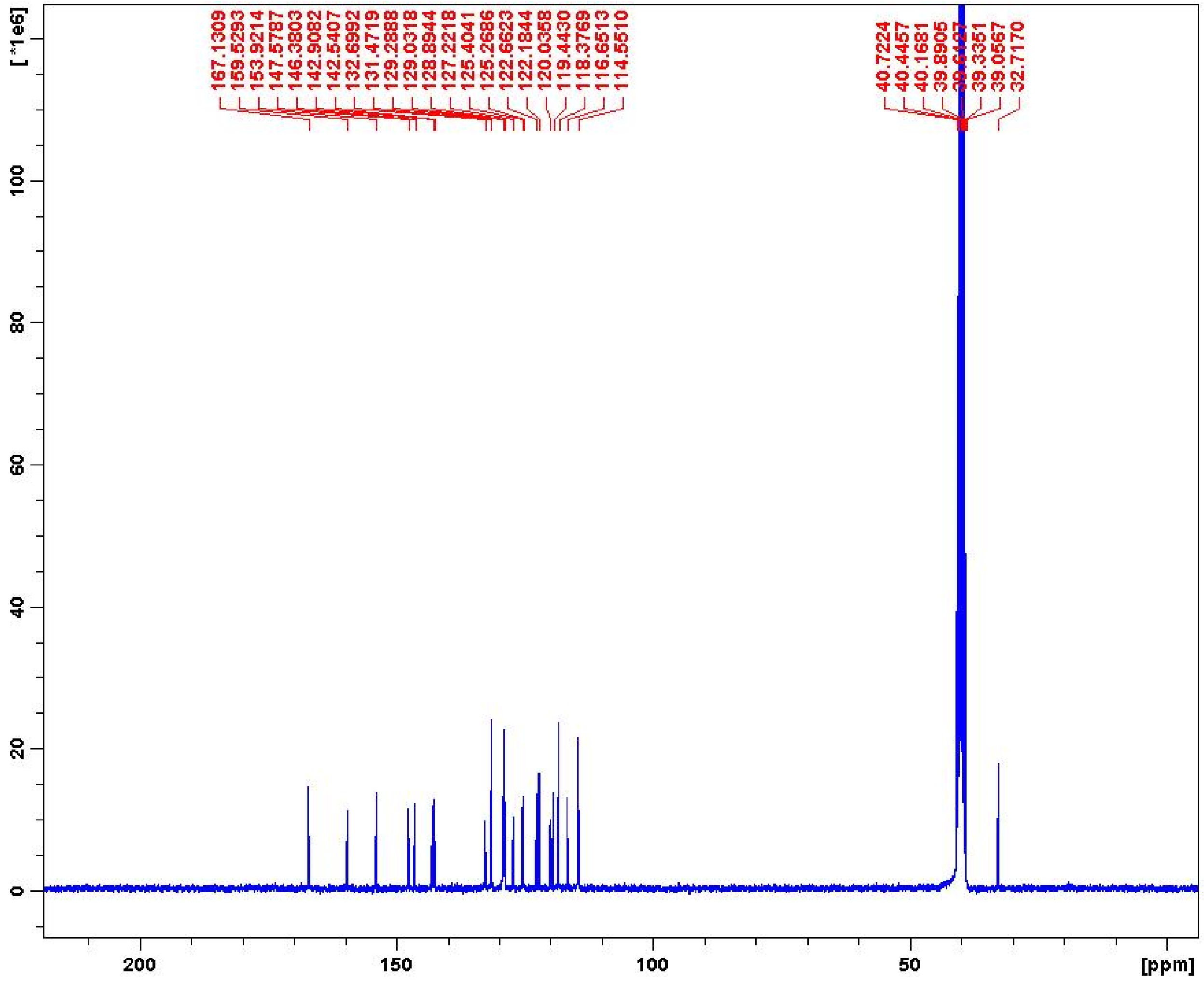

167.1309
159.5293
153.9214
147.5787
146.3803
142.9082
142.5407
132.6992
131.4719
129.2888
129.0318
128.8944
127.2218
125.4041
125.2686
122.6623
122.1844
120.0358
119.4430
118.3769
116.6513
114.5510
40.7224
40.4457
40.1681
39.8905
39.6127
39.3351
39.0567
32.7170
[*1e6]
[ppm]

4-[4-[(E)-[benzyl(phenyl)hydrazono]methyl]-3-(2-oxochromen-3-yl)pyrazol-1-yl]benzoic acid (7)

^{1}H NMR spectrum

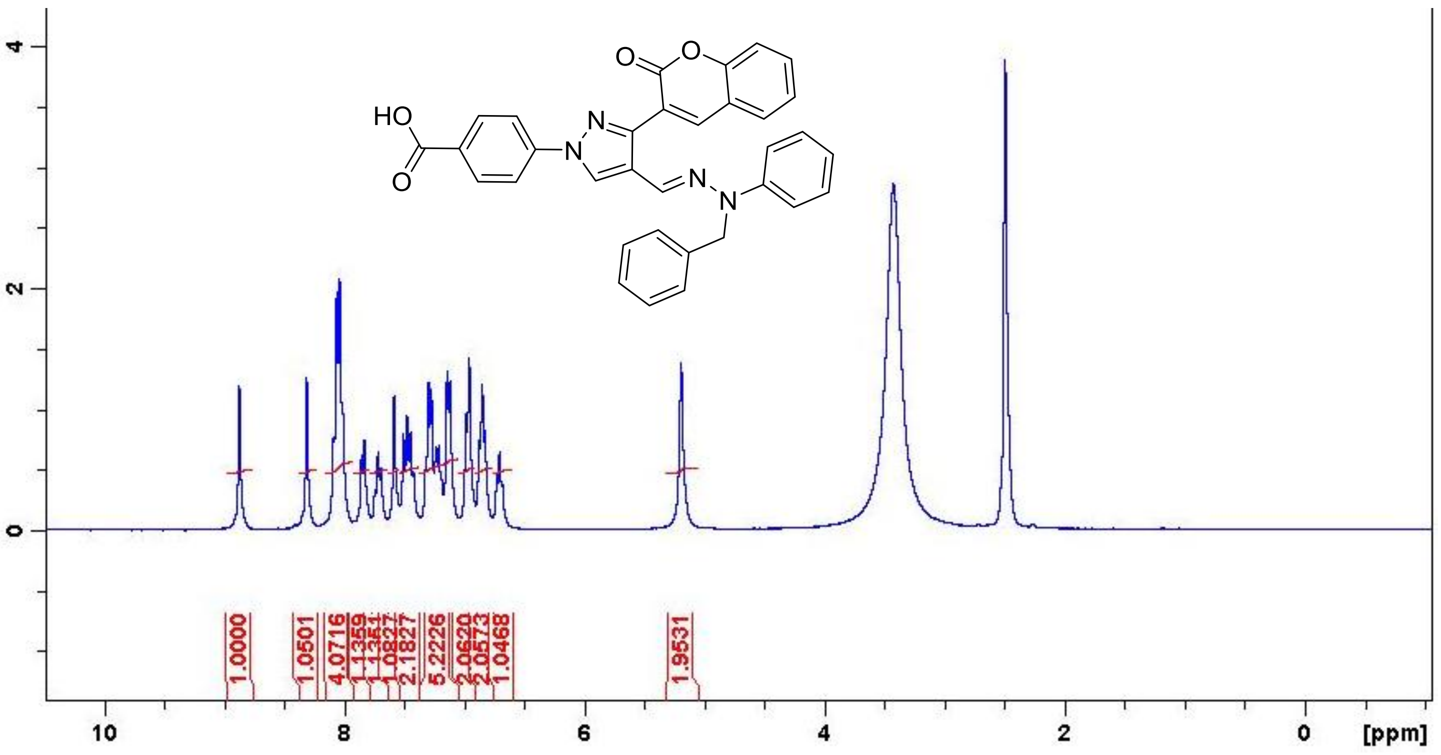

^{13}C NMR spectrum of compound **7**

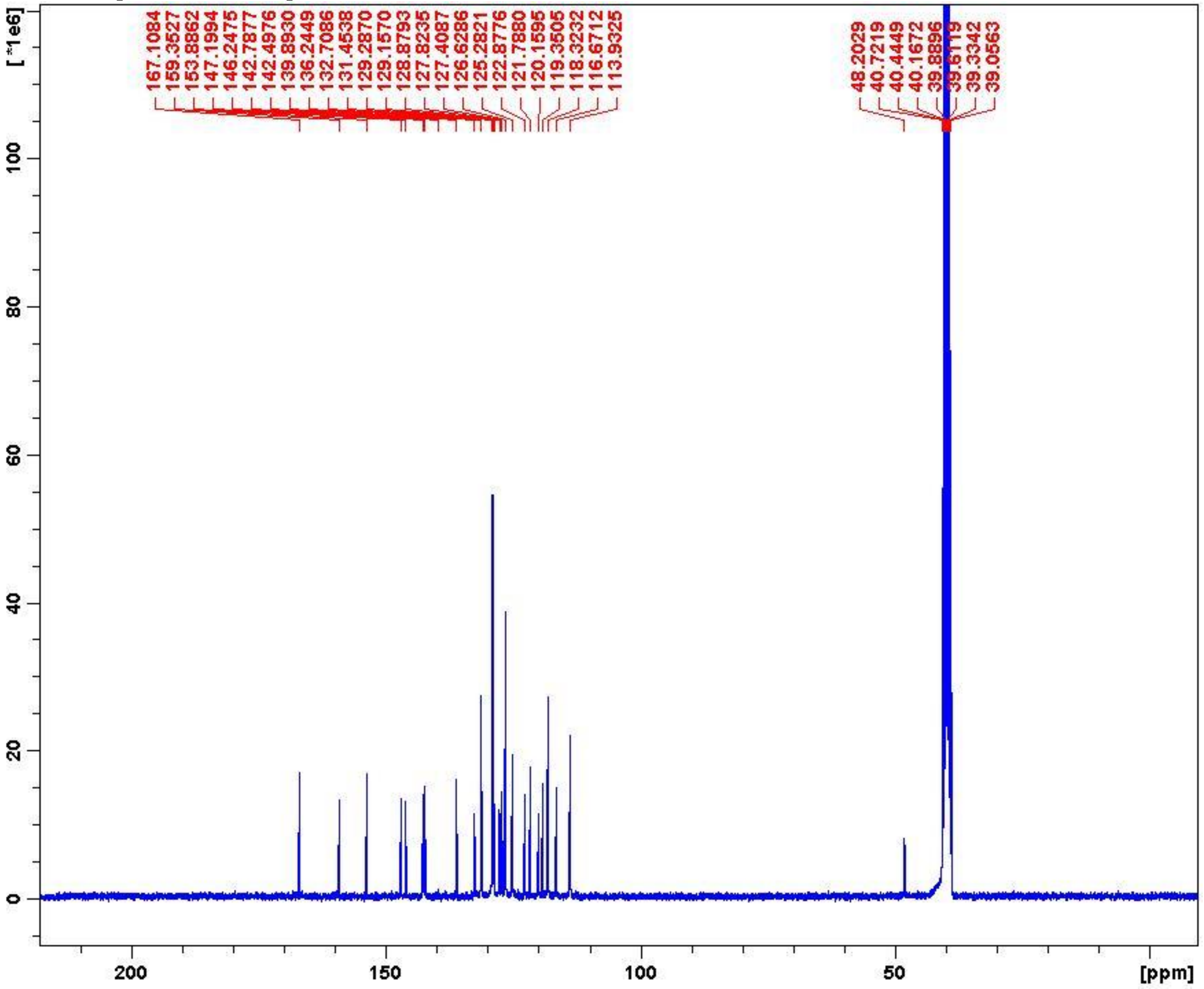

4-[4-[(E)-(diphenylhydrazono)methyl]-3-(2-oxochromen-3-yl)pyrazol-1-yl]benzoic acid (8)

^{1}H NMR spectrum

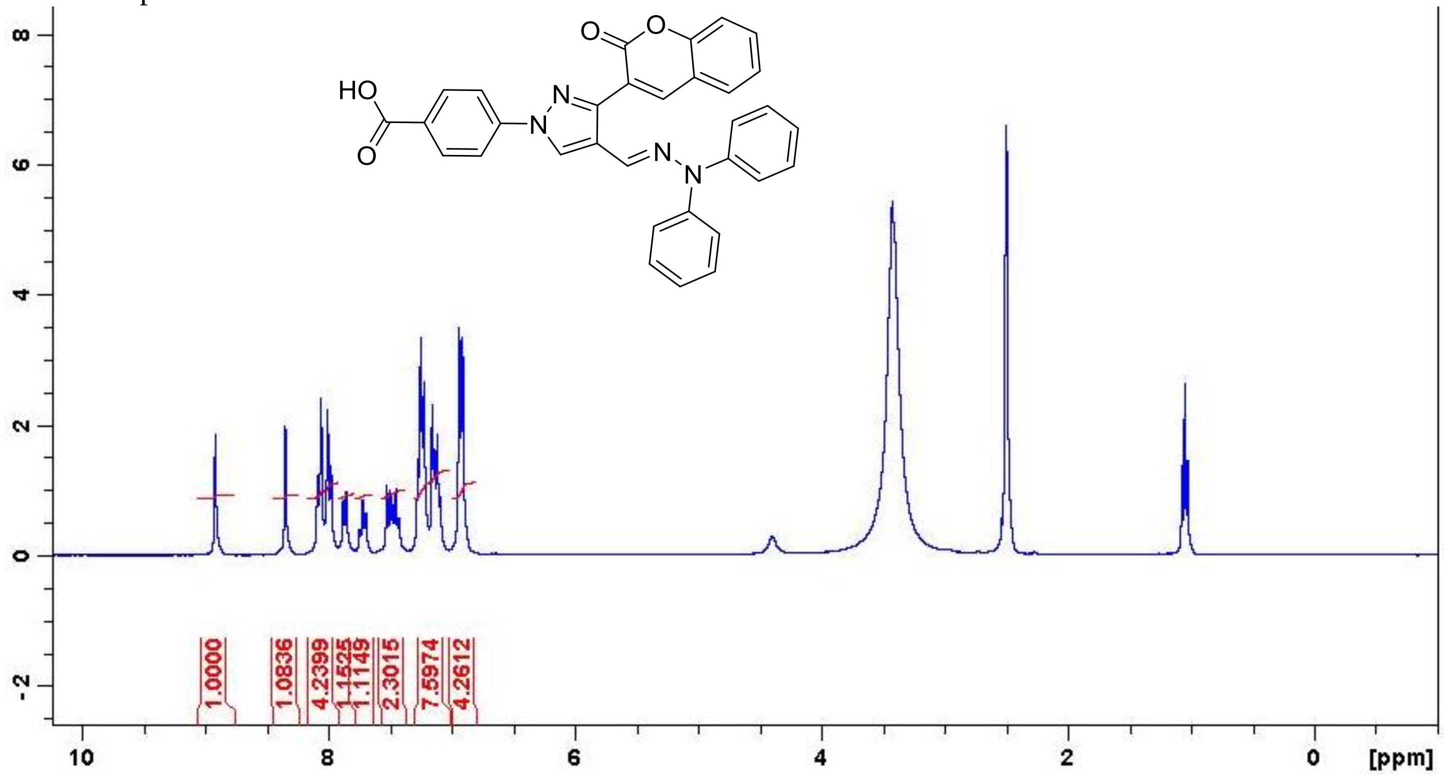

^{13}C NMR spectrum of compound **8**

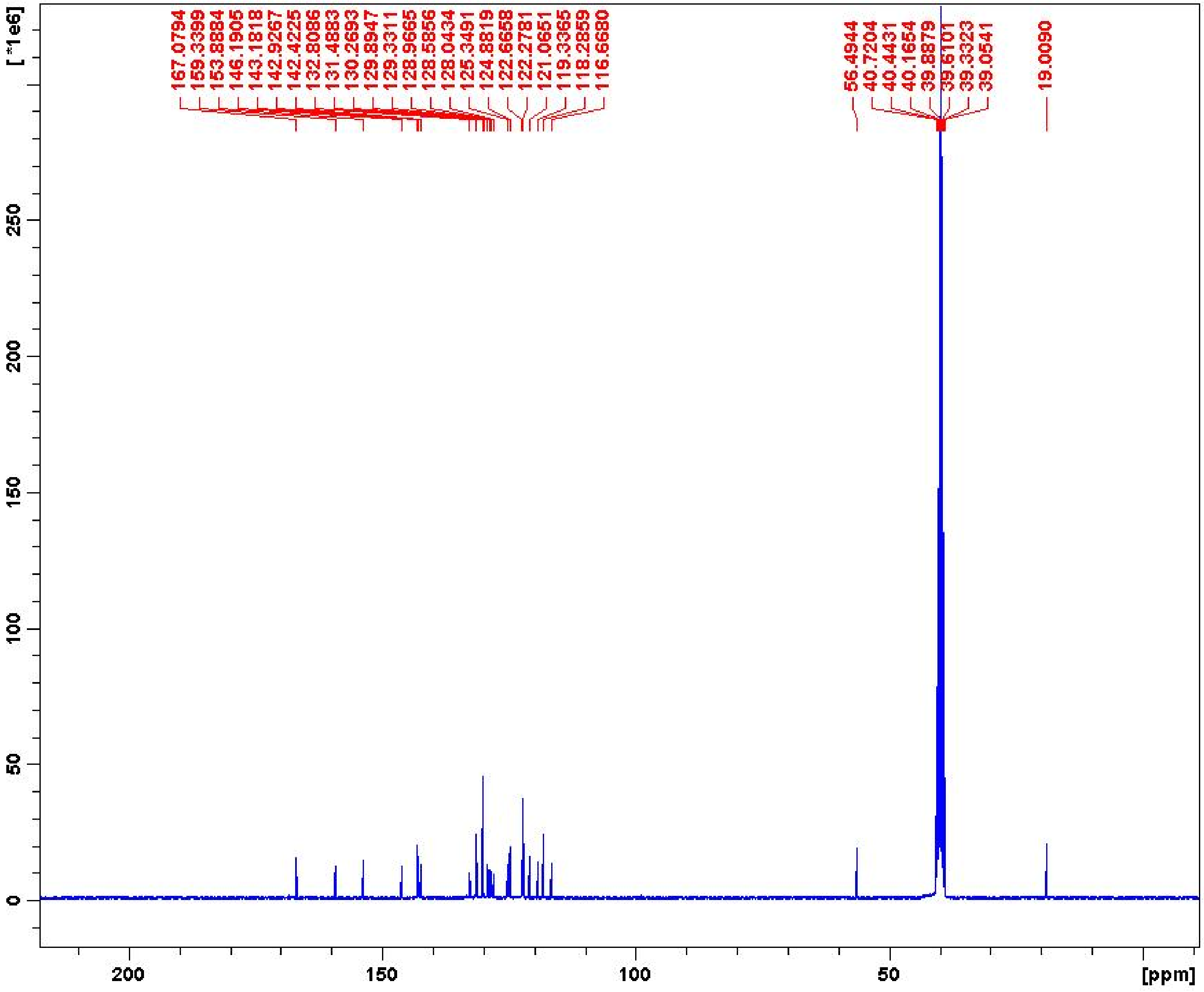

4-[4-[(E)-[(2-ethylphenyl)hydrazono]methyl]-3-(2-oxochromen-3-yl)pyrazol-1-yl]benzoic acid (9)

^{1}H NMR spectrum

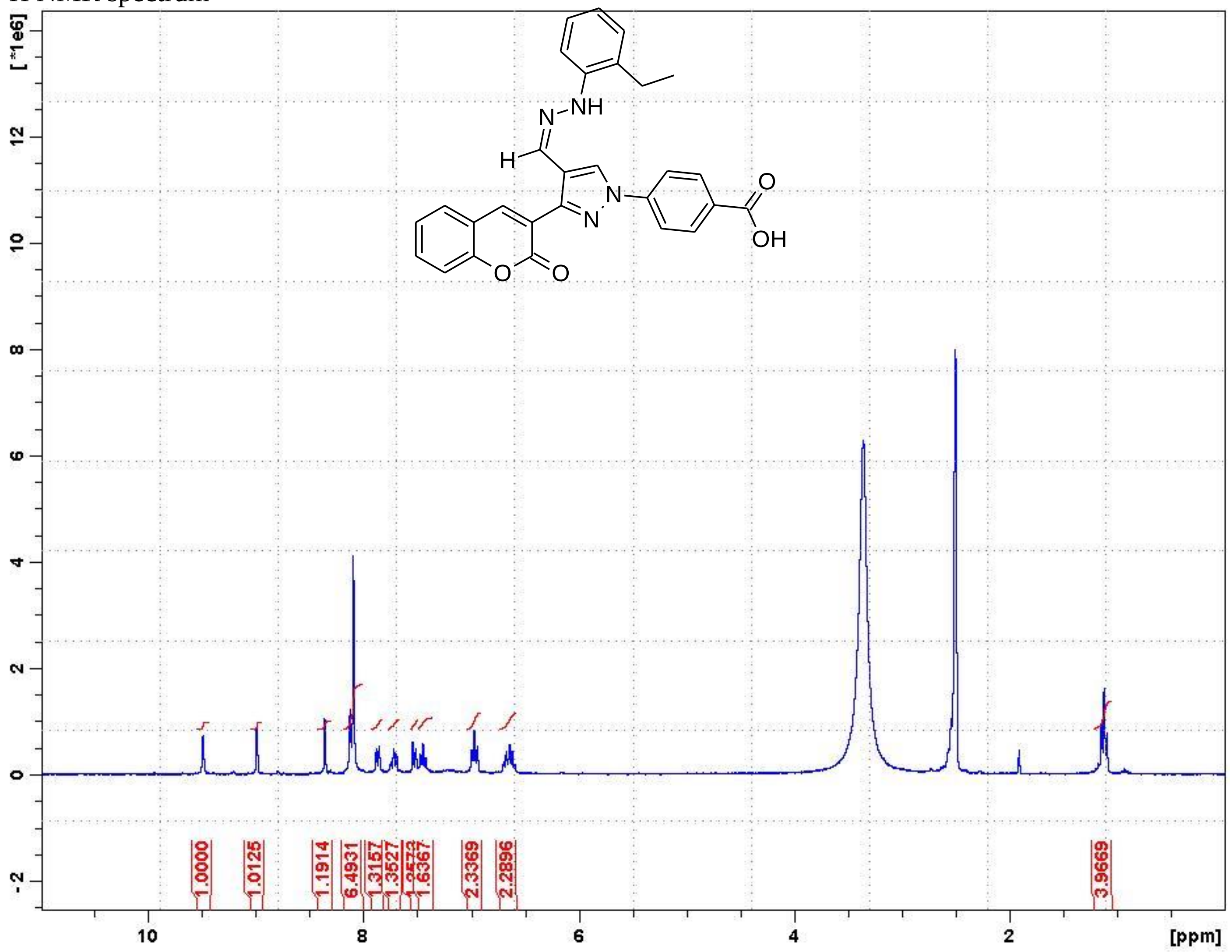

^{13}C NMR spectrum of compound **9**

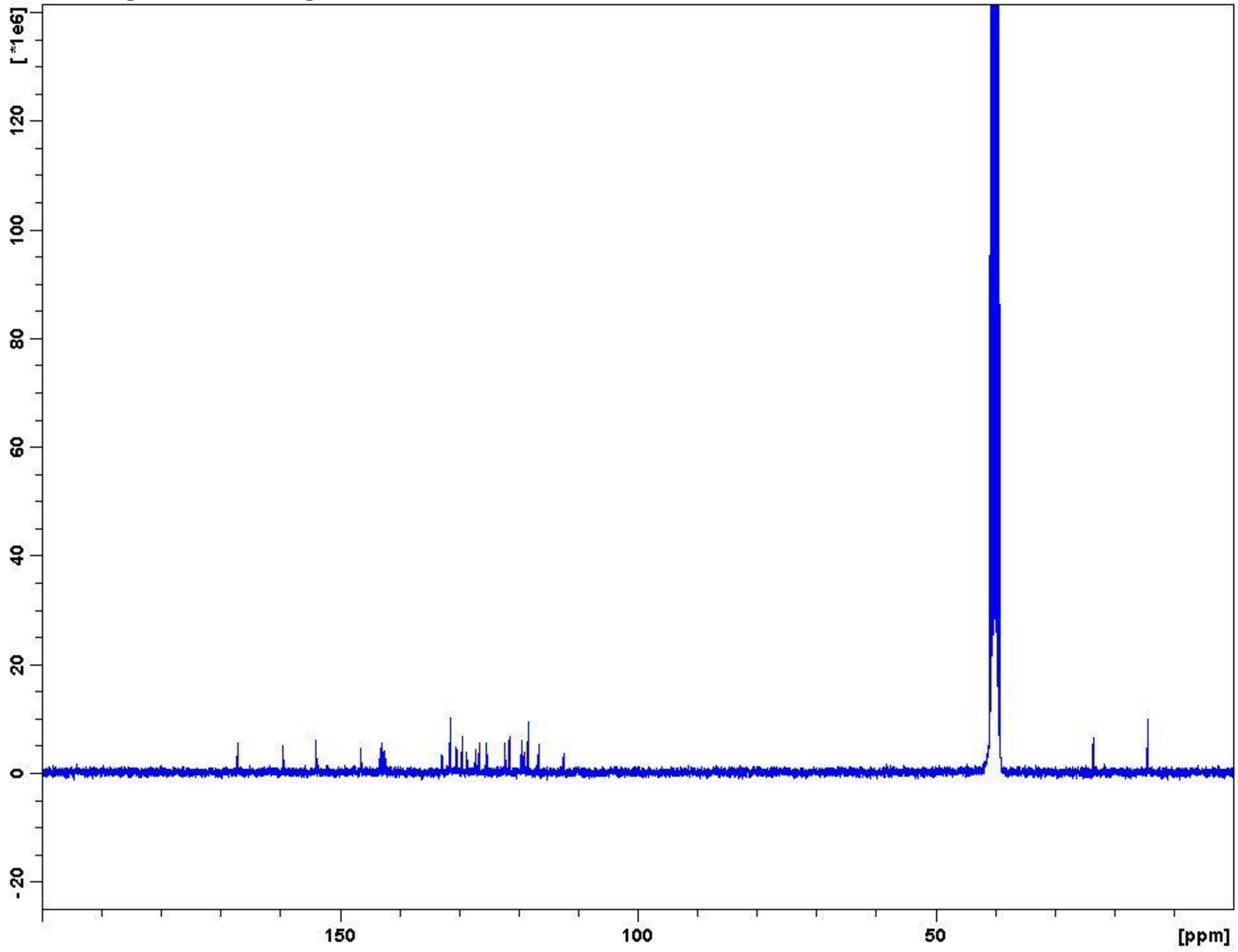

4-[4-[(E)-[(4-methoxyphenyl)hydrazono]methyl]-3-(2-oxochromen-3-yl)pyrazol-1-yl]benzoic acid (10)

^{1}H NMR spectrum

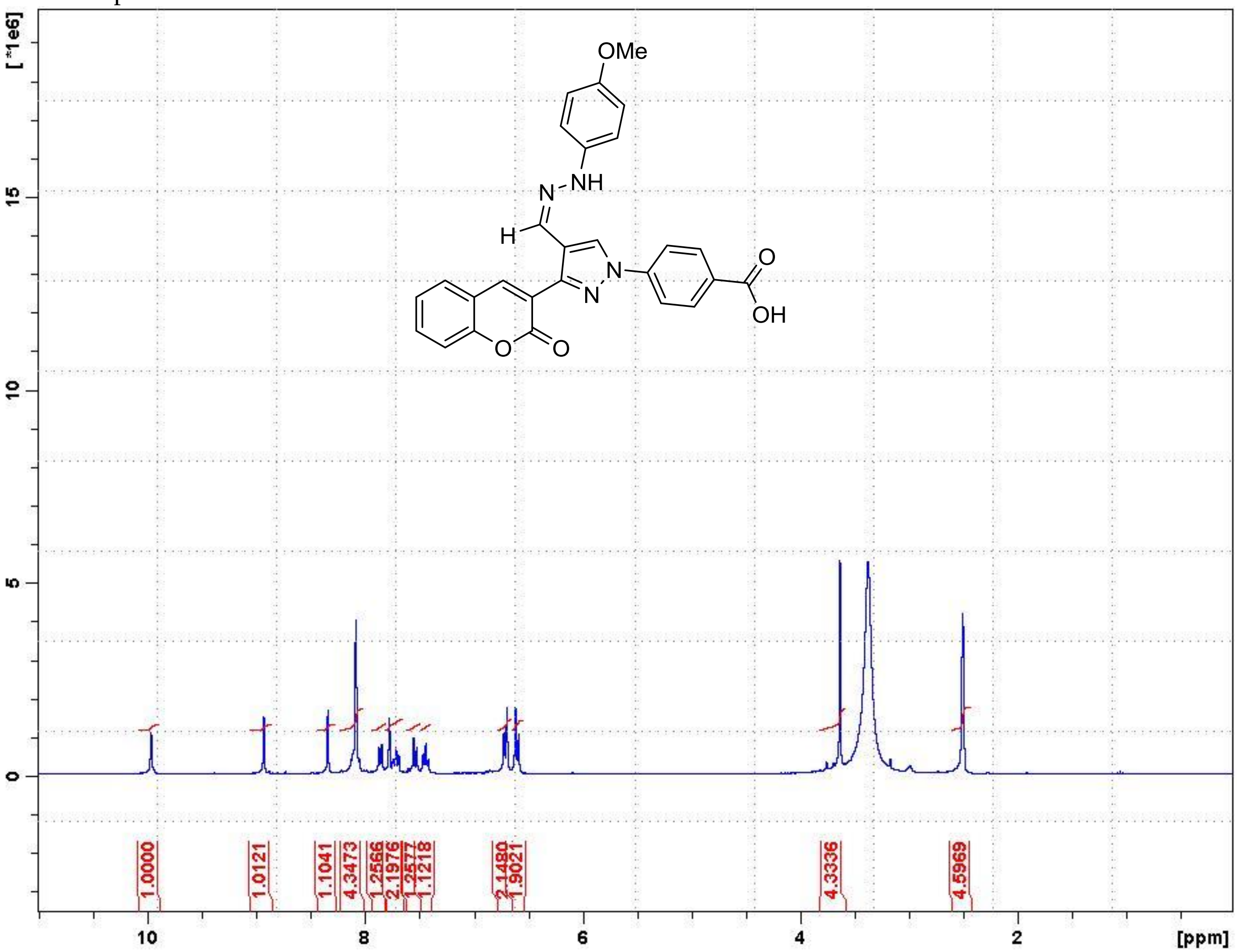

^{13}C NMR spectrum of compound **10**

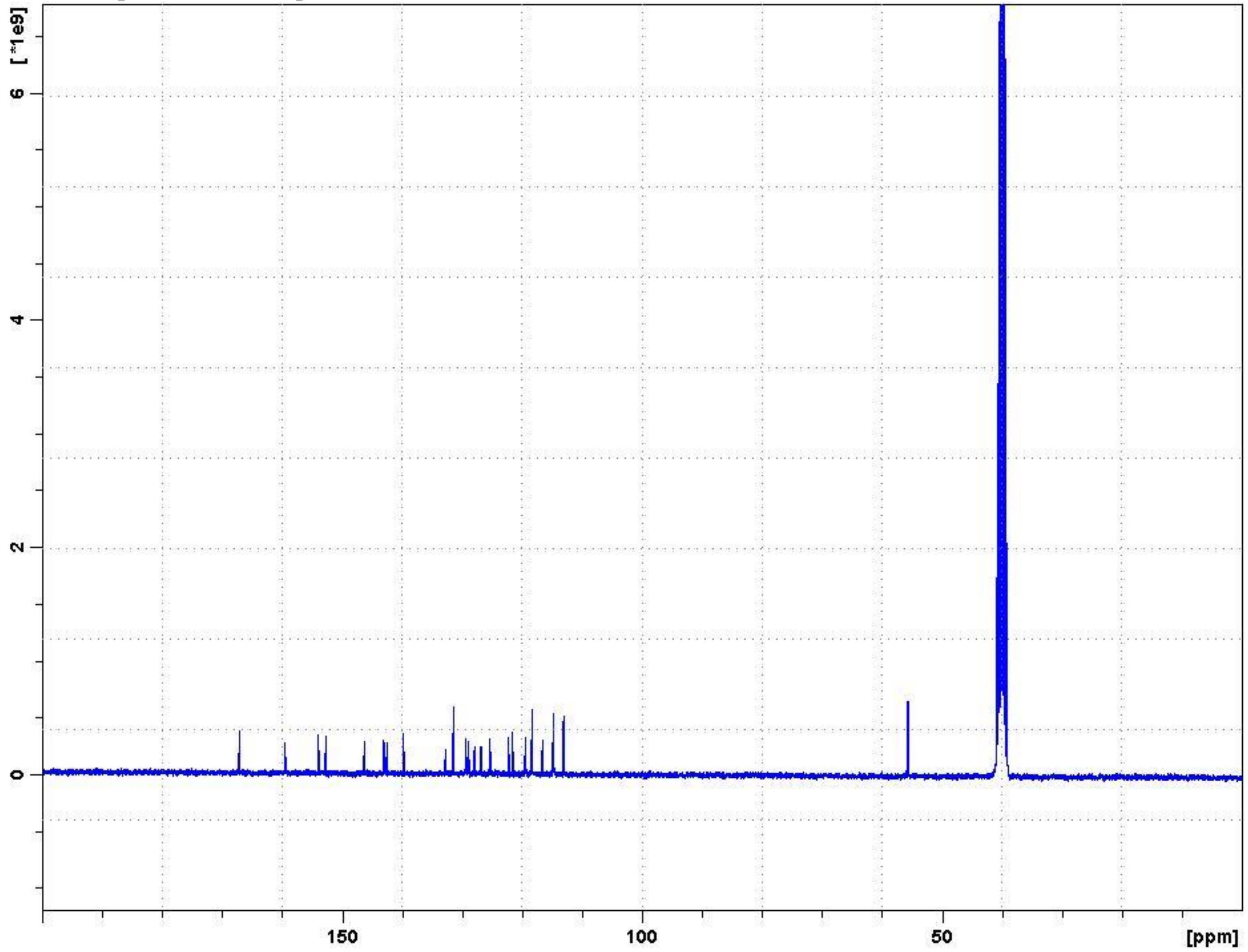

4-[4-[(E)-[(2-fluorophenyl)hydrazono]methyl]-3-(2-oxochromen-3-yl)pyrazol-1-yl]benzoic acid (11)

^{1}H NMR spectrum

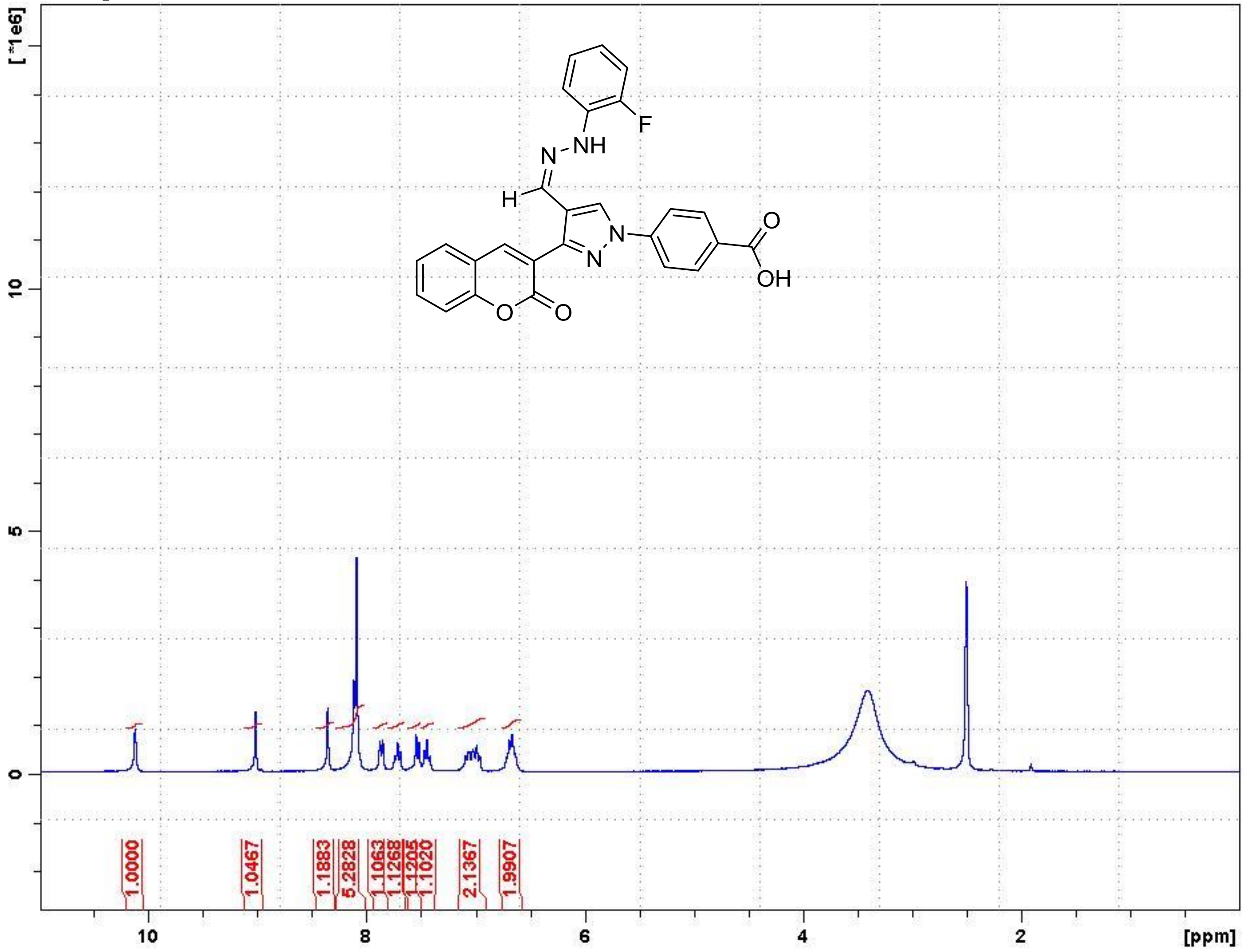

^{13}C NMR spectrum of compound **11**

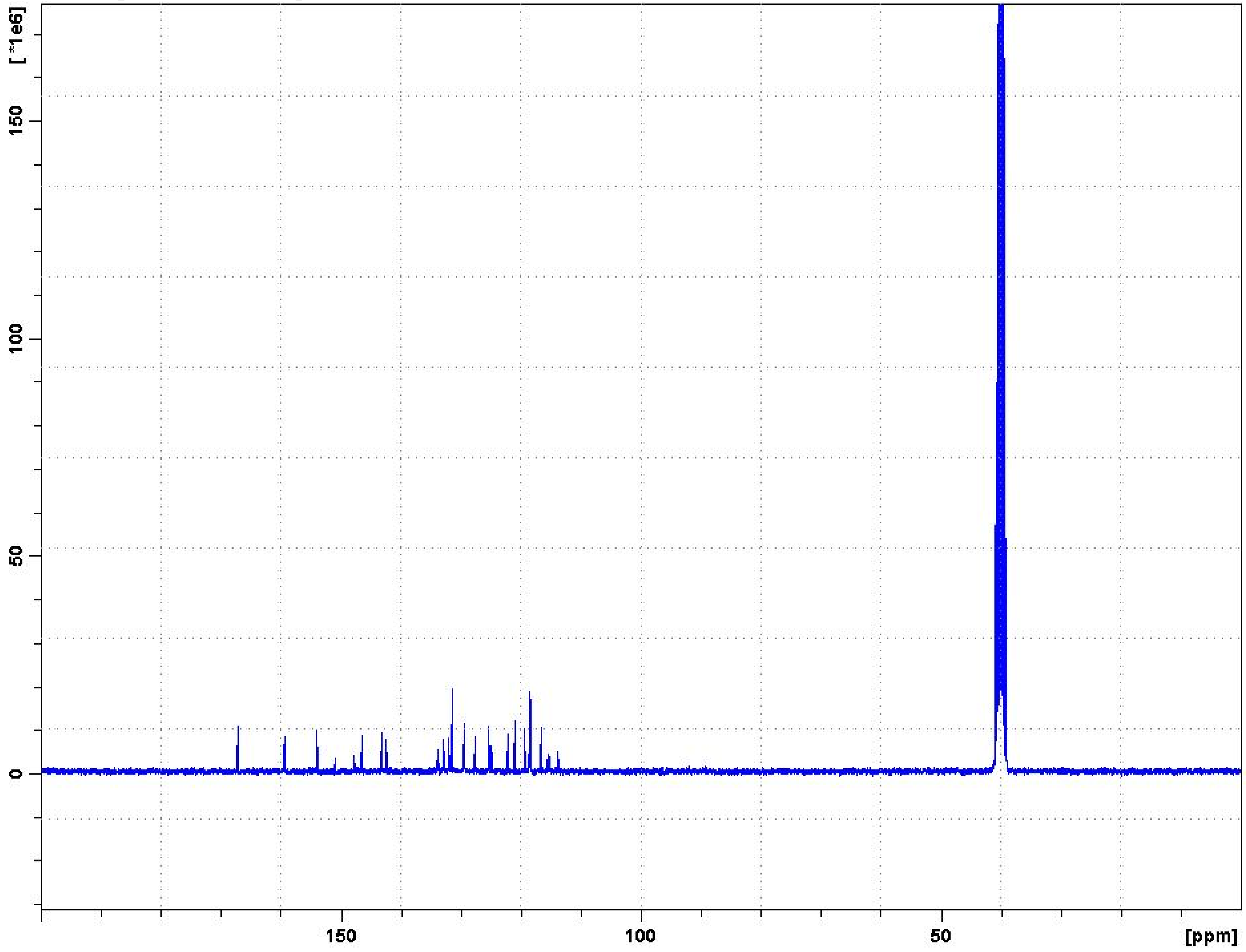

4-[4-[(E)-[(3-fluorophenyl)hydrazono]methyl]-3-(2-oxochromen-3-yl)pyrazol-1-yl]benzoic acid (12)

^{1}H NMR spectrum

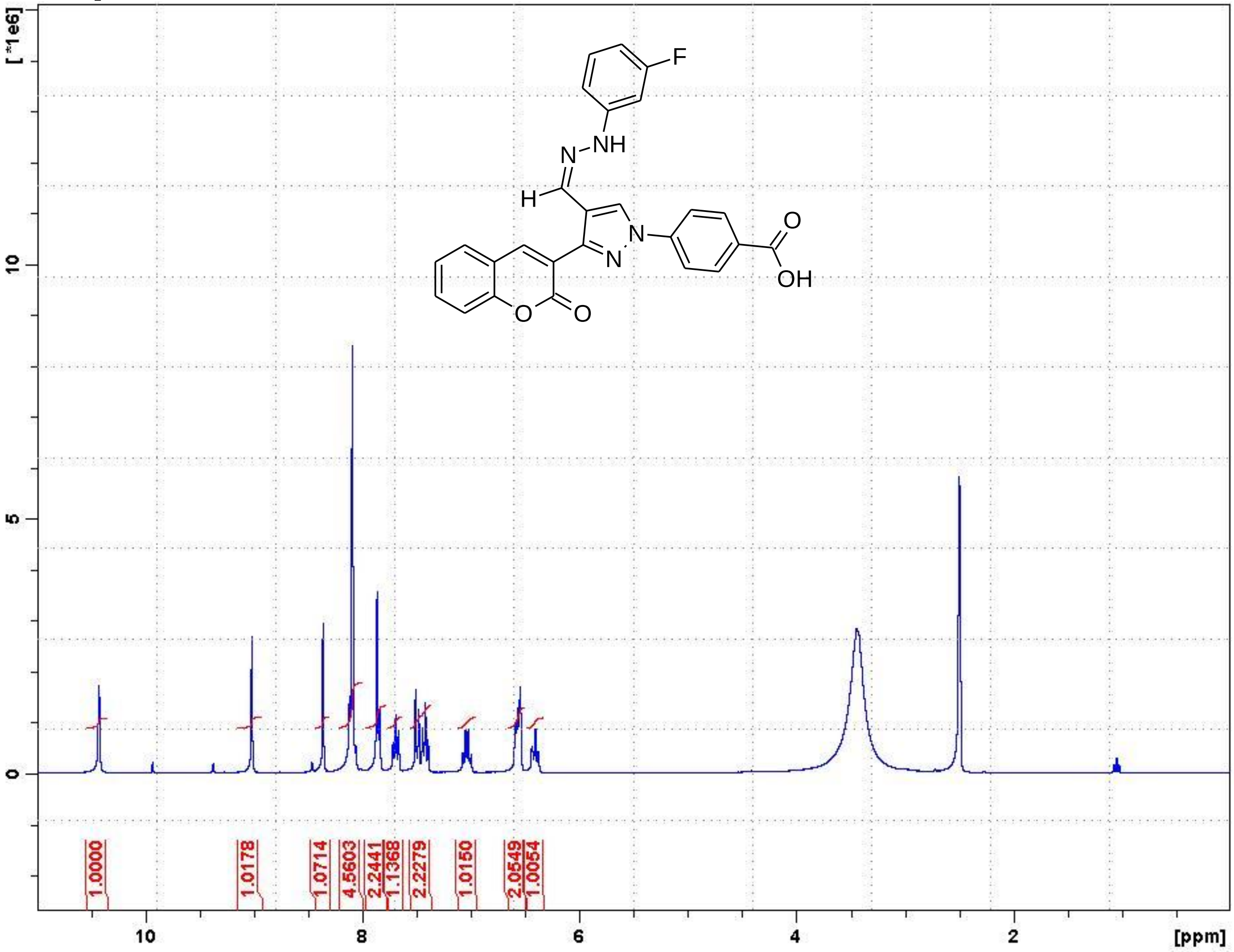

^{13}C NMR spectrum of compound **12**

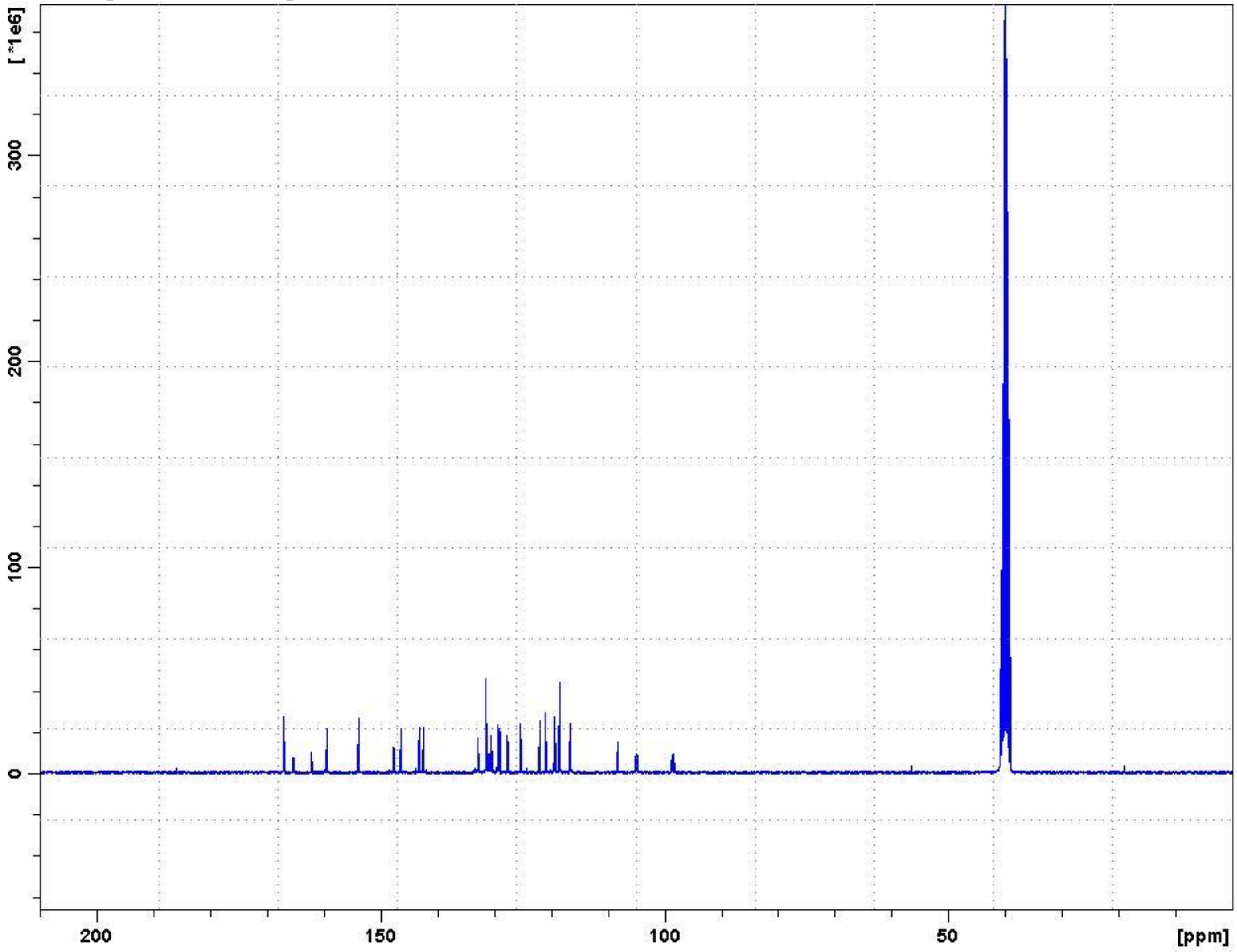

4-[4-[(E)-[(4-fluorophenyl)hydrazono]methyl]-3-(2-oxochromen-3-yl)pyrazol-1-yl]benzoic acid (13)

^{1}H NMR spectrum

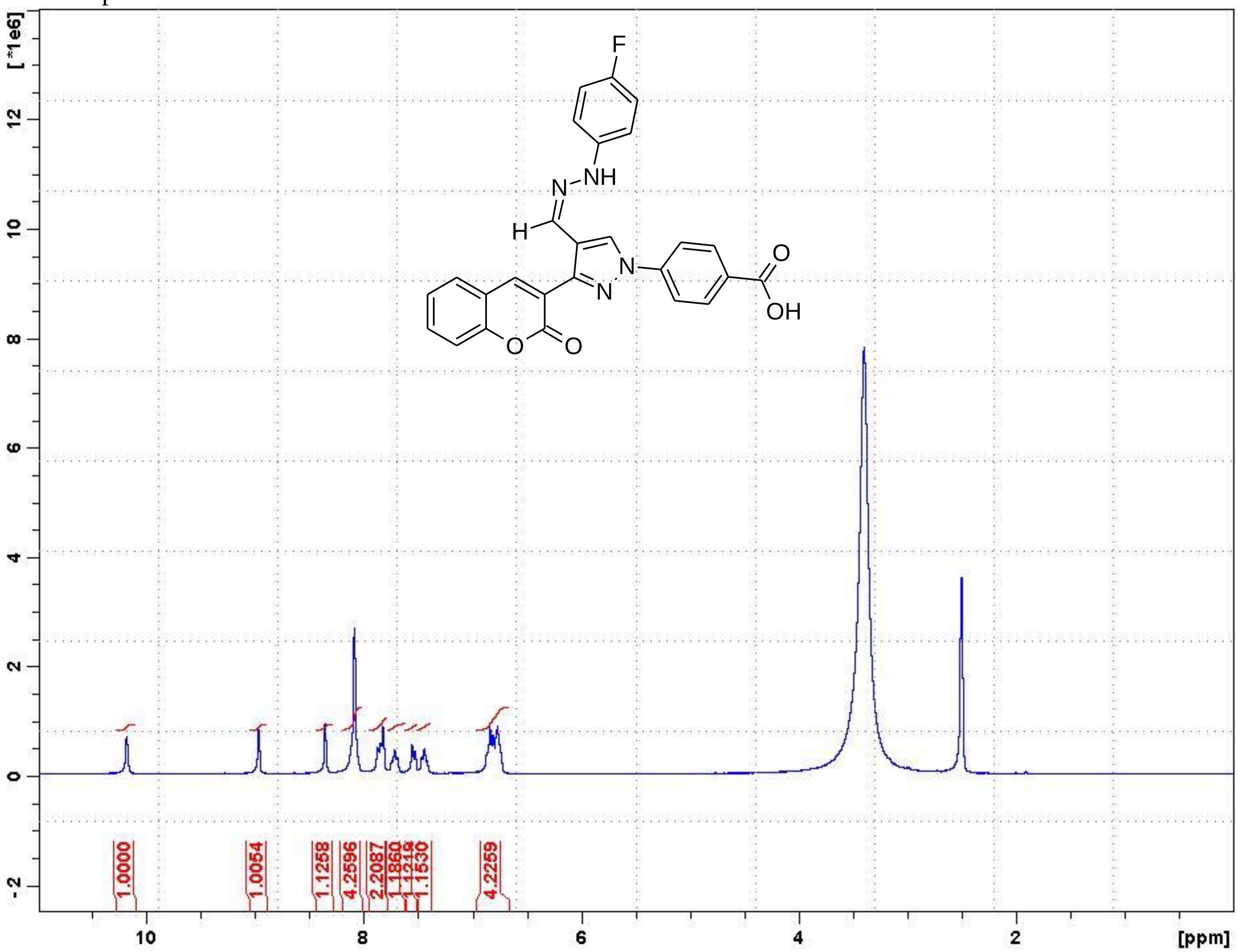

^{13}C NMR spectrum of compound **13**

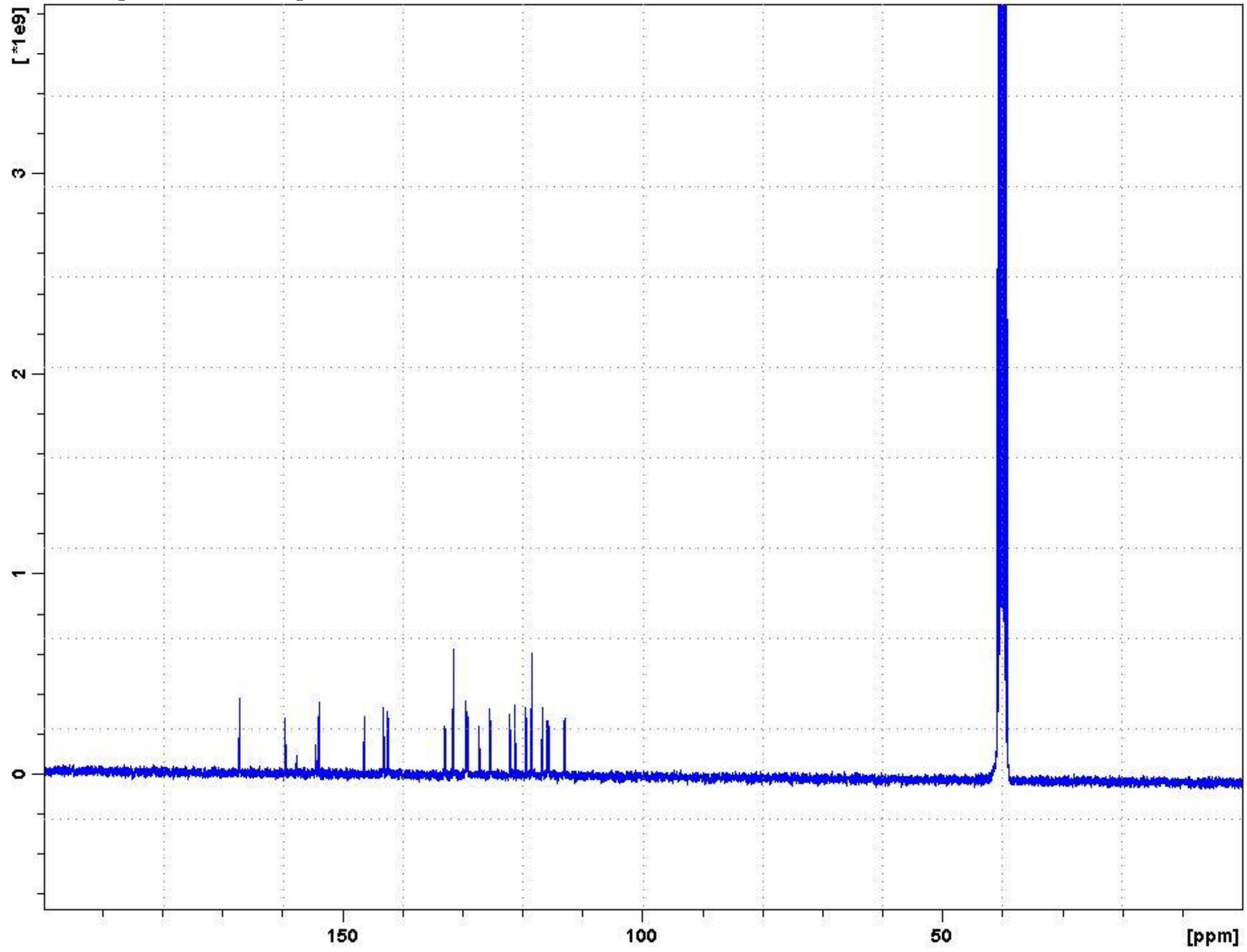

4-[4-[(E)-[(3-chlorophenyl)hydrazono]methyl]-3-(2-oxochromen-3-yl)pyrazol-1-yl]benzoic acid (14)

^{1}H NMR spectrum

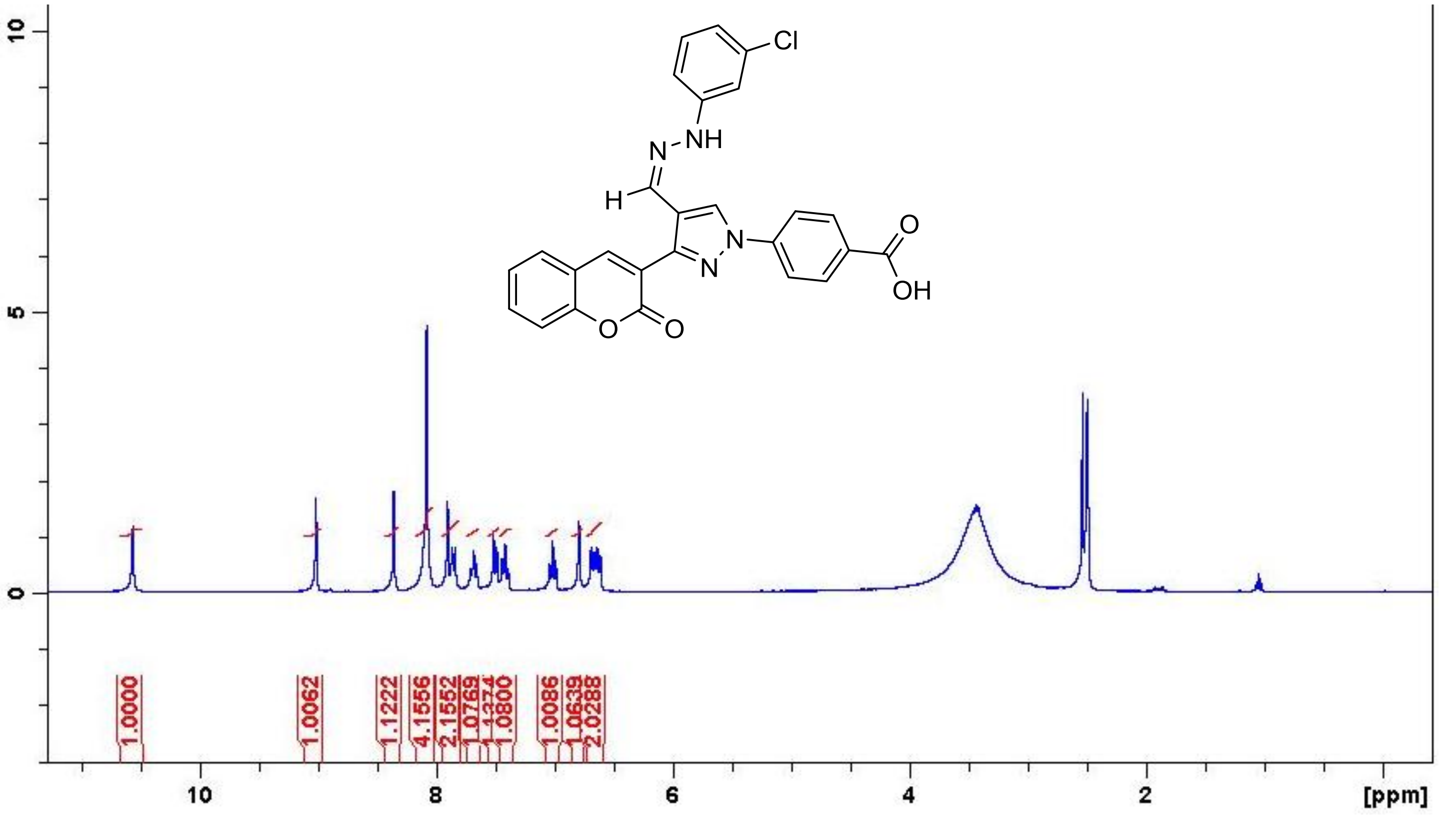

^{13}C NMR spectrum of compound **14**

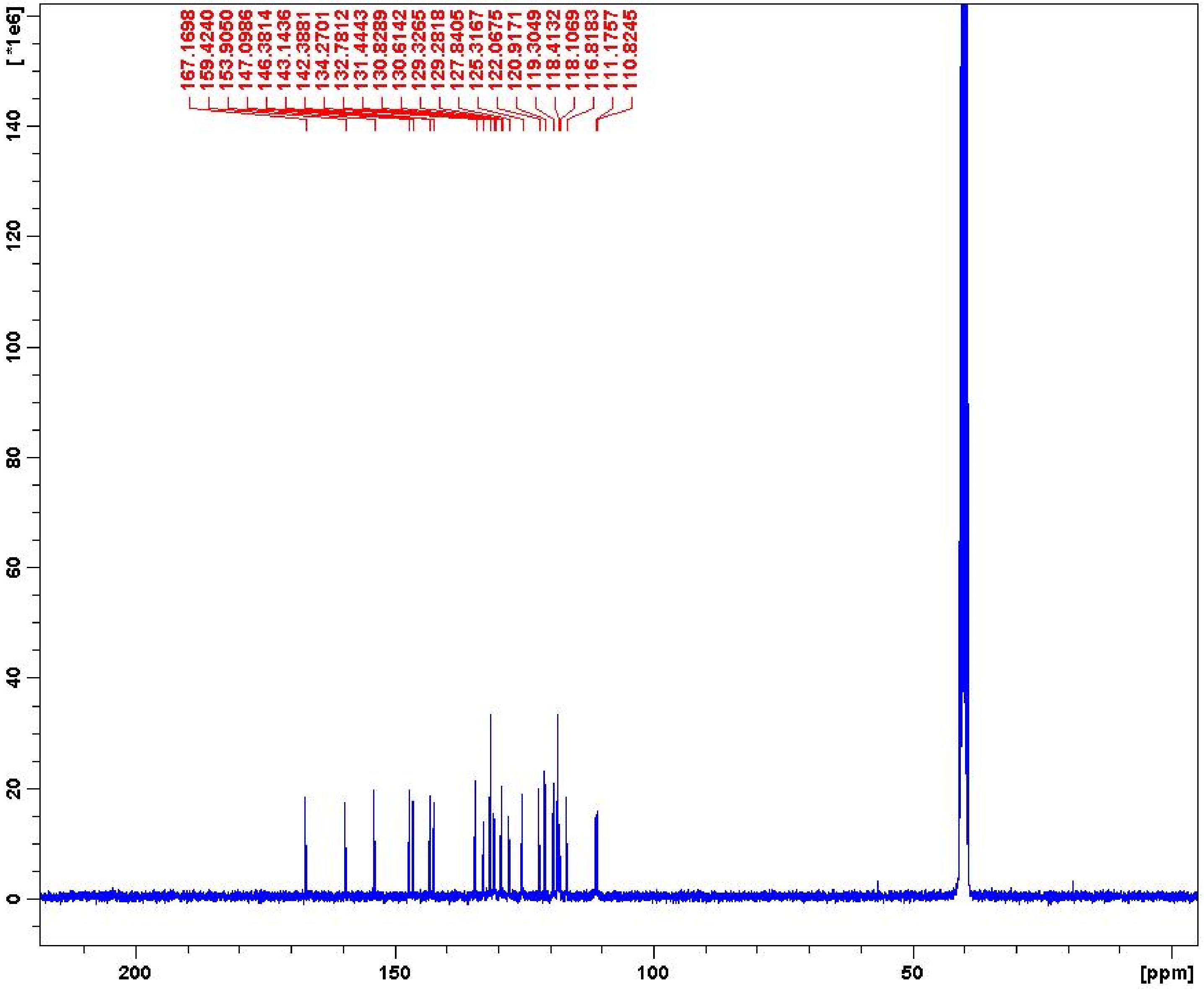

4-[4-[(E)-[(4-chlorophenyl)hydrazono]methyl]-3-(2-oxochromen-3-yl)pyrazol-1-yl]benzoic acid (15)

^{1}H NMR spectrum

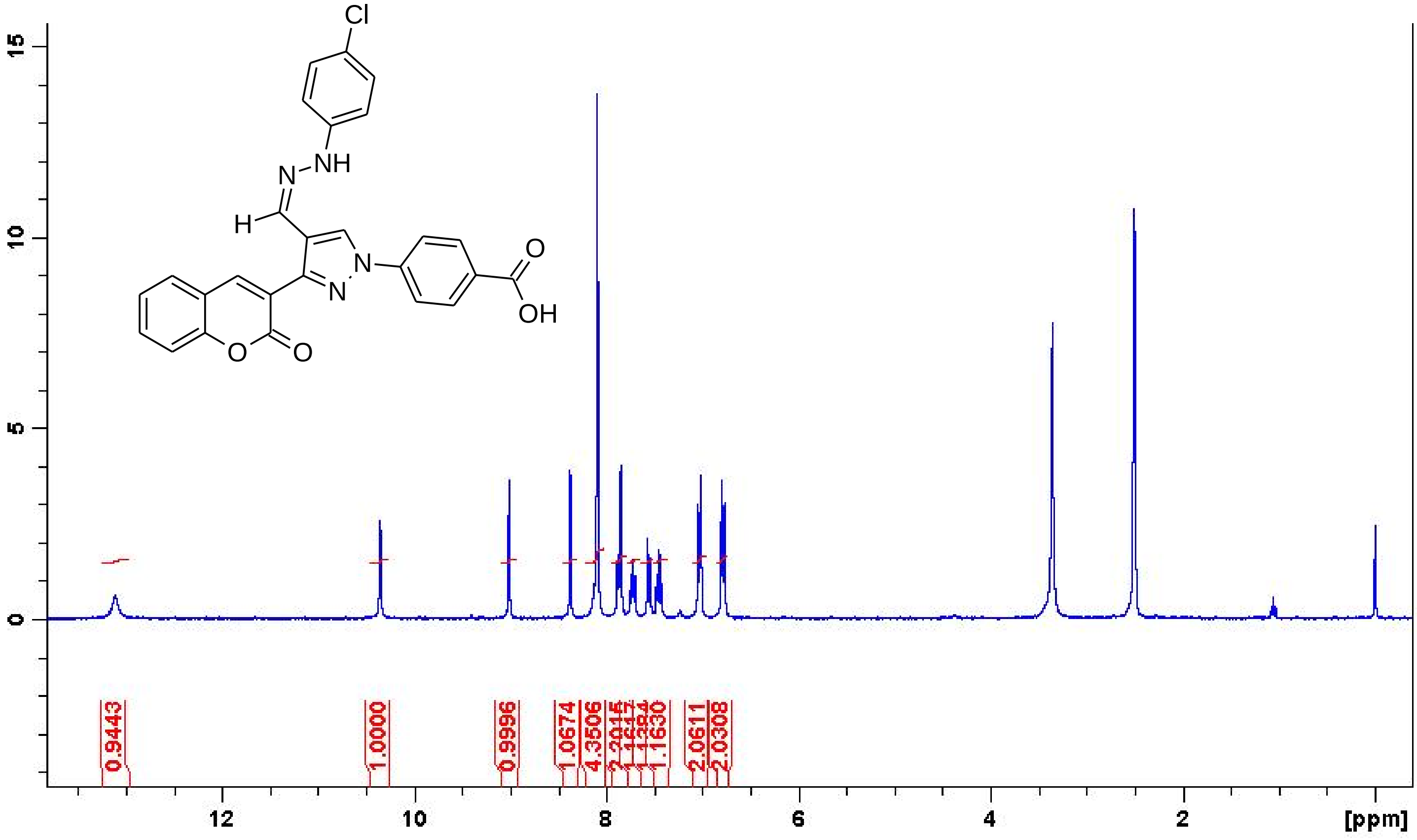

^{13}C NMR spectrum of compound **15**

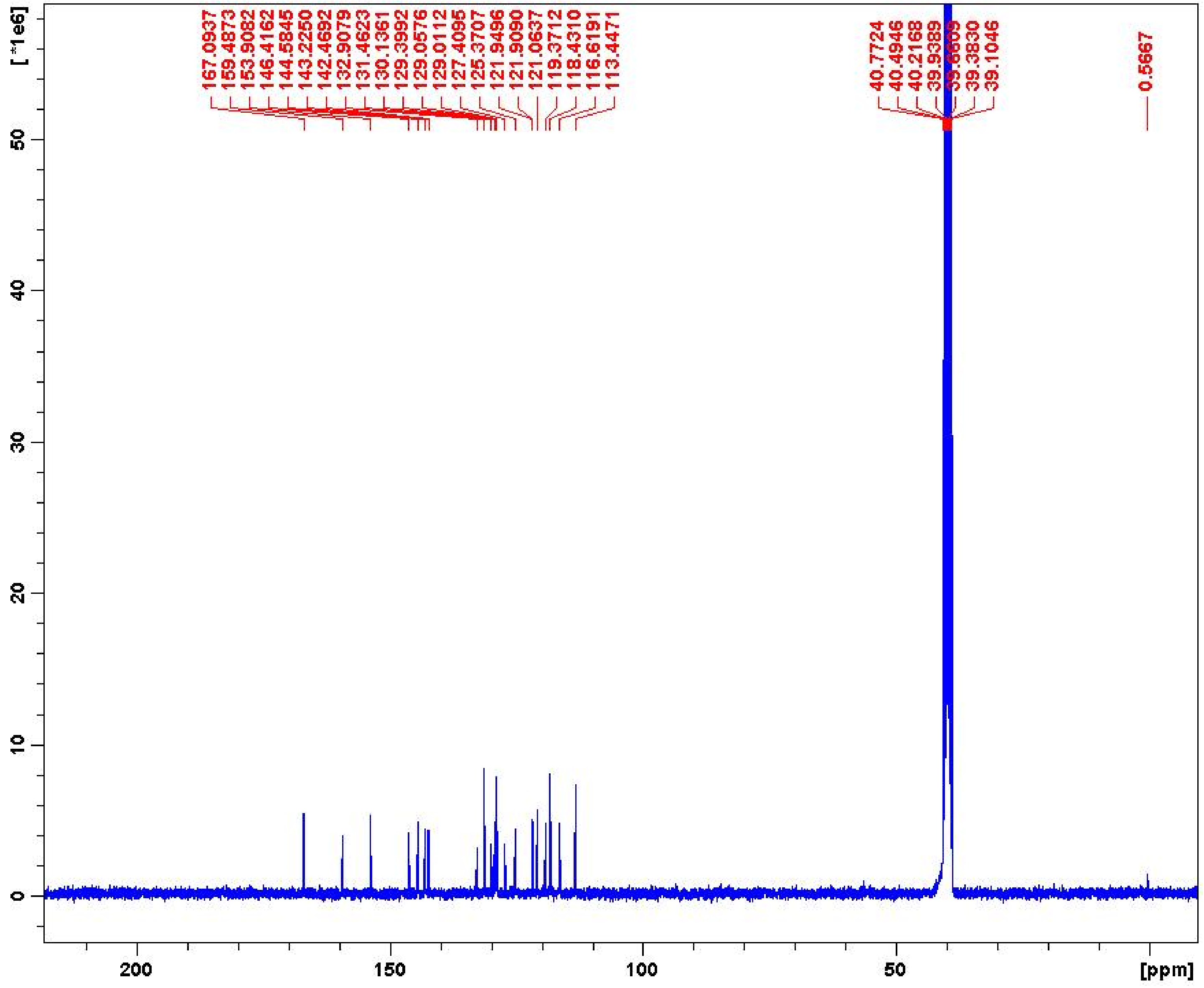

4-[4-[(E)-[(3-bromo)hydrazono]methyl]-3-(2-oxochromen-3-yl)pyrazol-1-yl]benzoic acid (16)

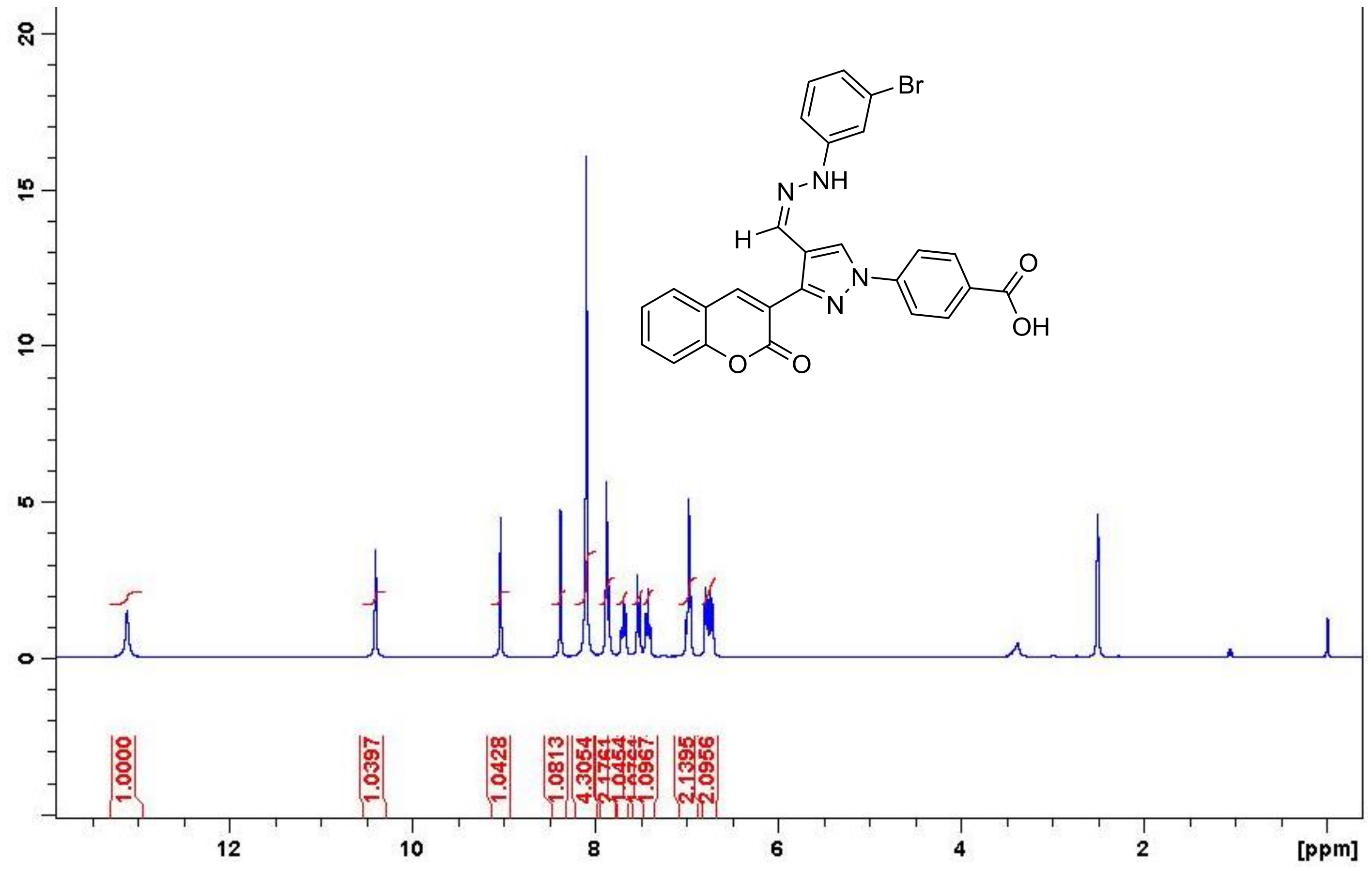

^{13}C NMR spectrum of compound **16**

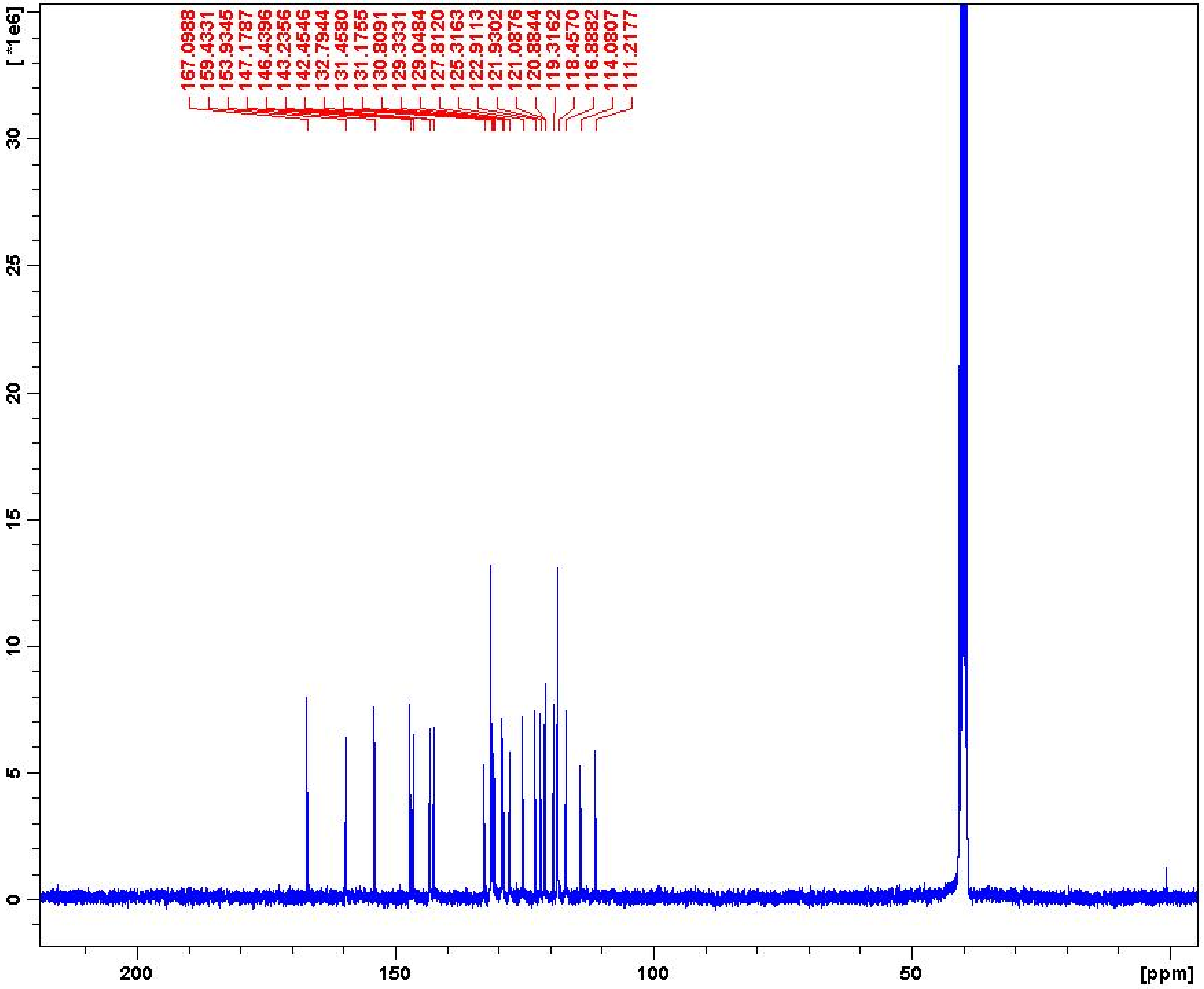

4-[4-[(E)-[(4-bromophenyl)hydrazono]methyl]-3-(2-oxochromen-3-yl)pyrazol-1-yl]benzoic acid (17)

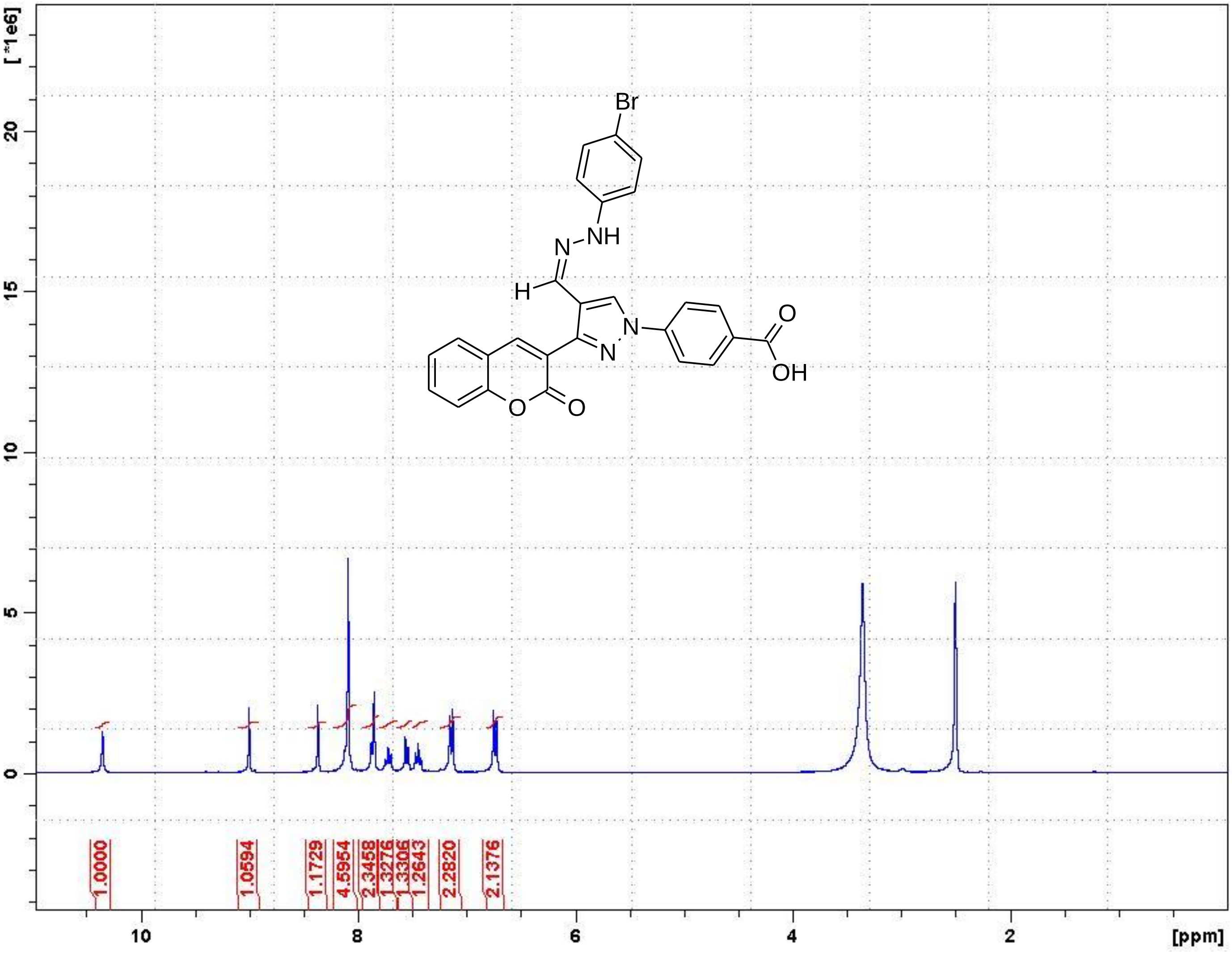

^{13}C NMR spectrum of compound **17**

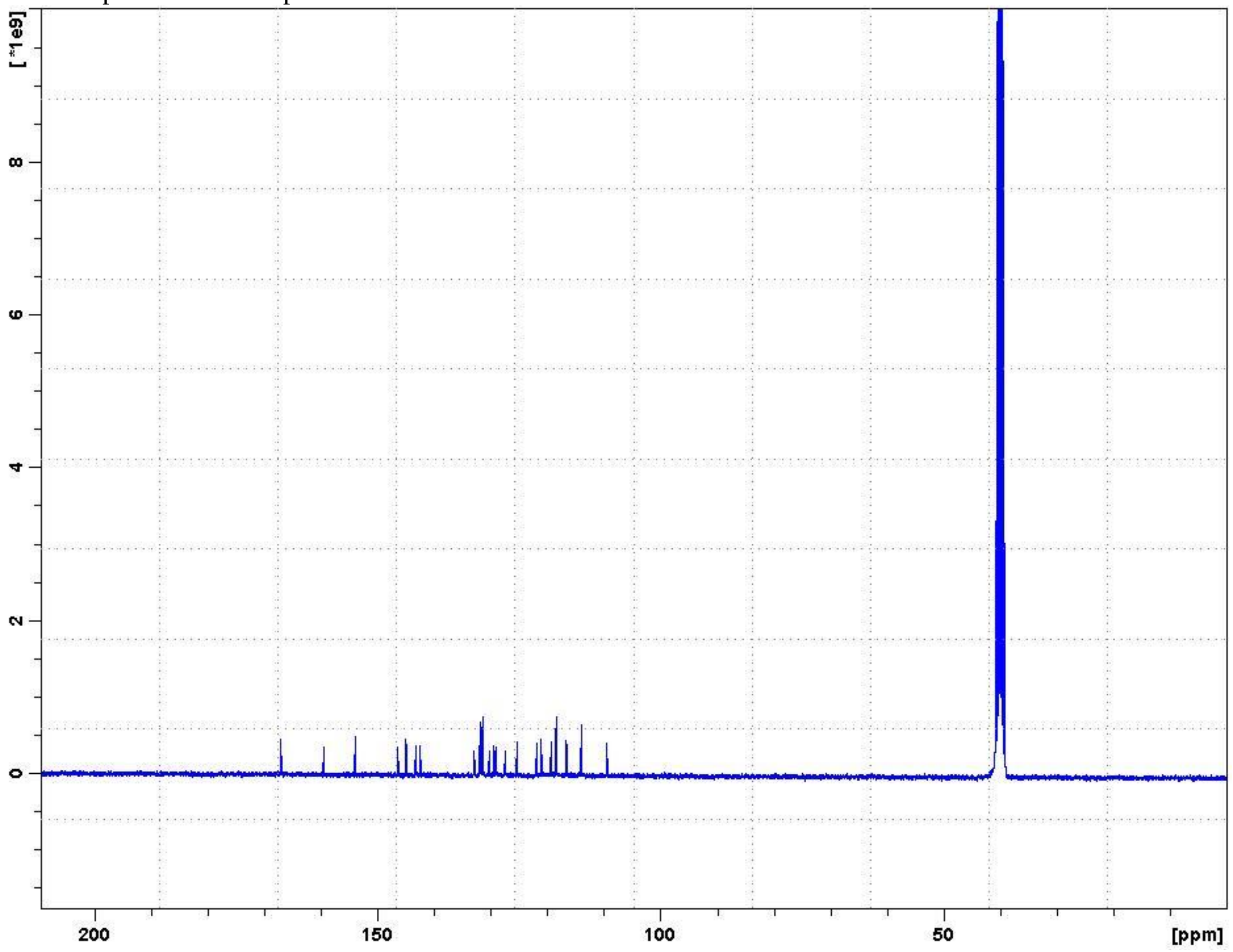

4-[4-[(E)-[(2,5-difluorophenyl)hydrazono]methyl]-3-(2-oxochromen-3-yl)pyrazol-1-yl]benzoic acid (18)

^{1}H NMR spectrum

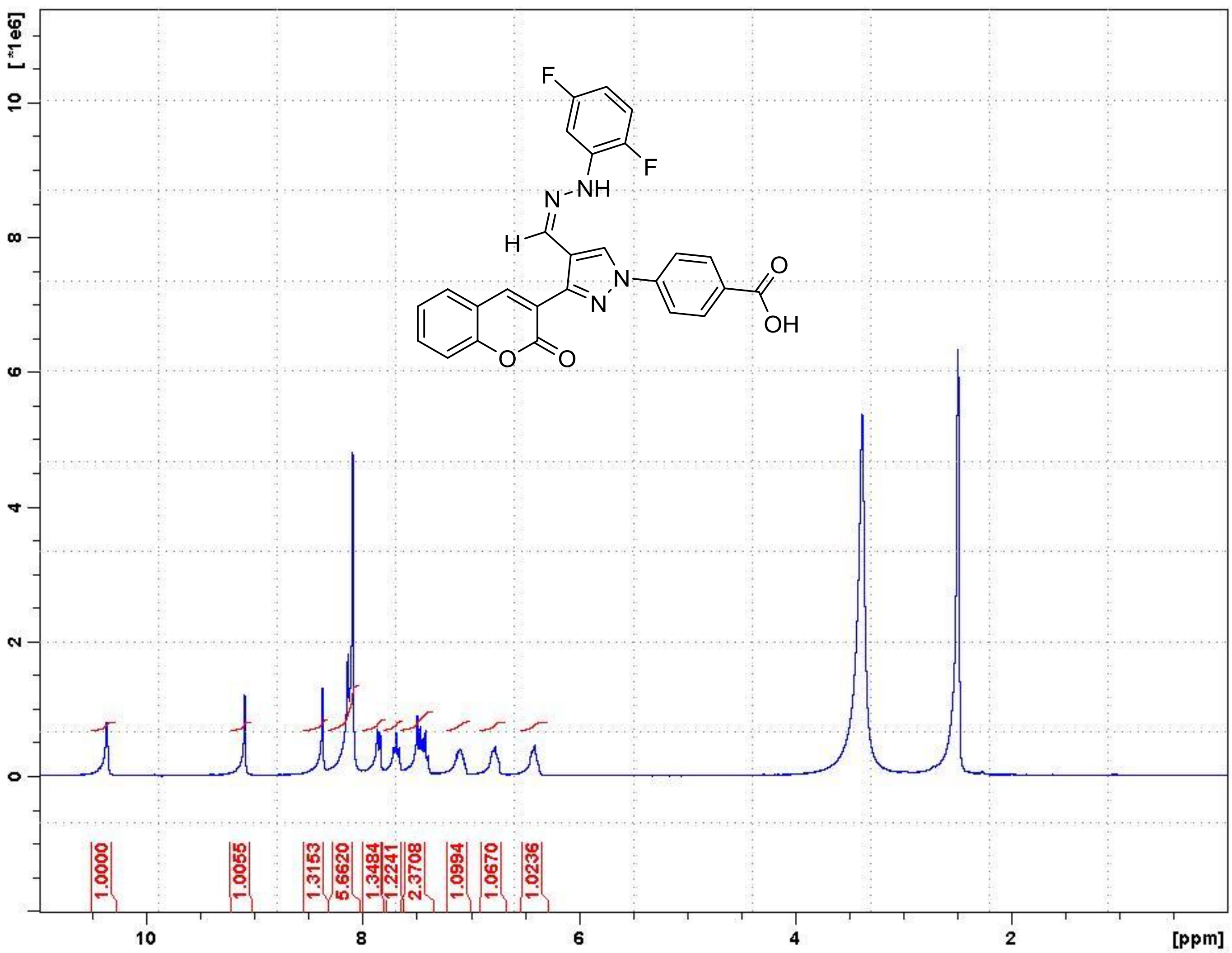

^{13}C NMR spectrum of compound **18**

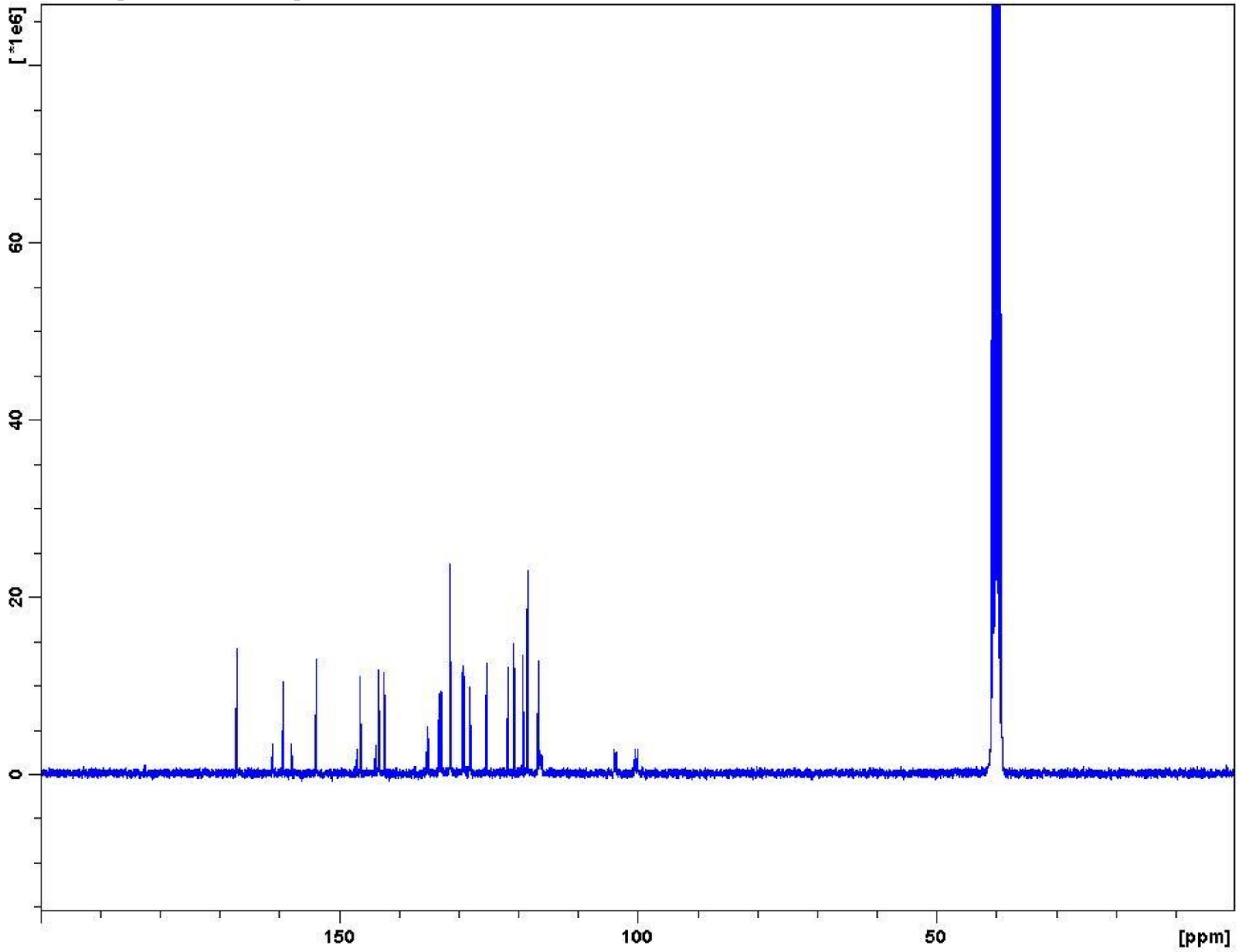

4-[3-(2-oxochromen-3-yl)-4-[(E)-[(2,3,5,6-tetrafluorophenyl)hydrazono]methyl]pyrazol-1-yl]benzoic acid (19)

^{1}H NMR spectrum

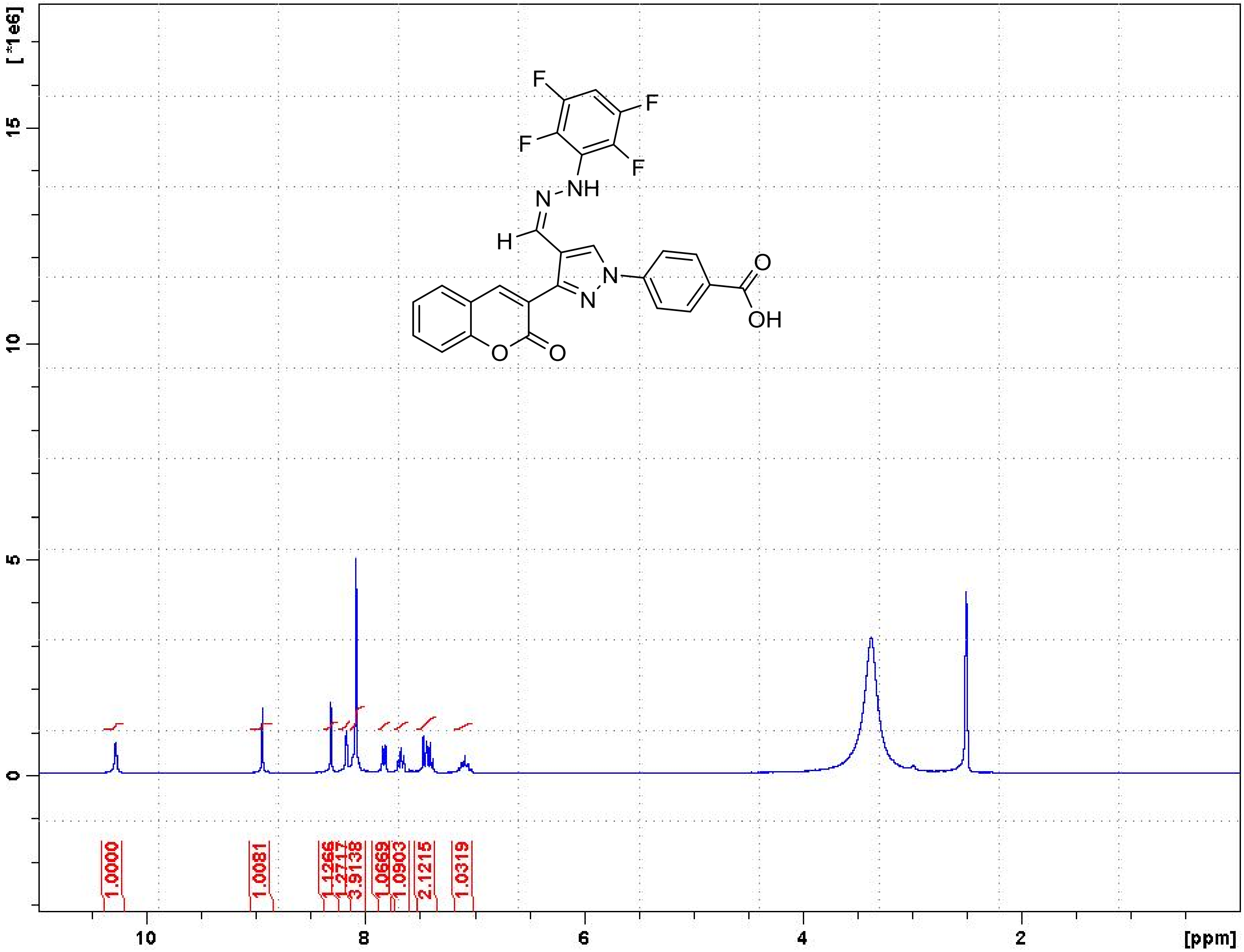

^{13}C NMR spectrum of compound **19**

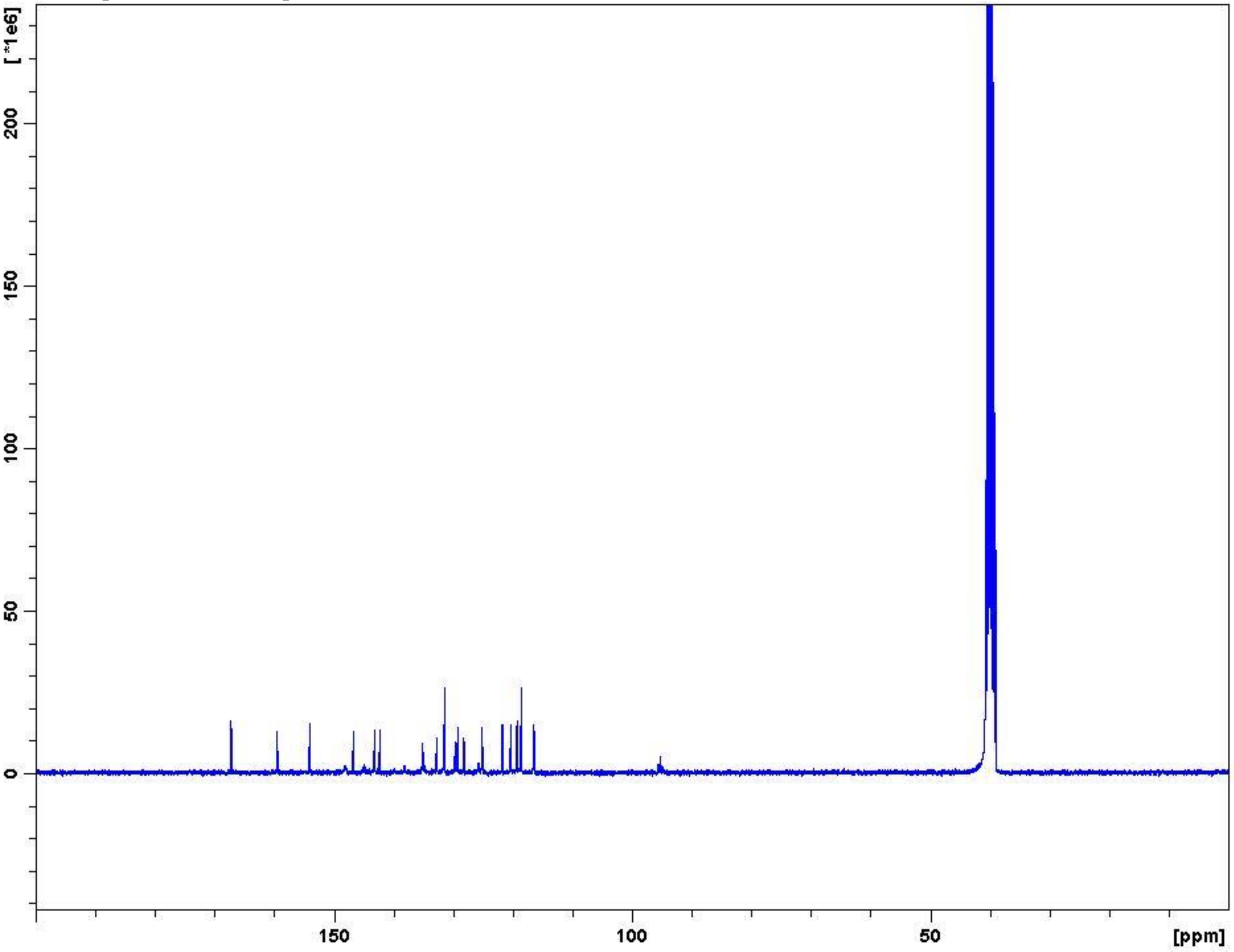

4-[3-(2-oxochromen-3-yl)-4-[(E)-[(2,3,4,5,6-pentafluorophenyl)hydrazono]methyl]pyrazol-1-yl]benzoic acid (20)

^{1}H NMR spectrum

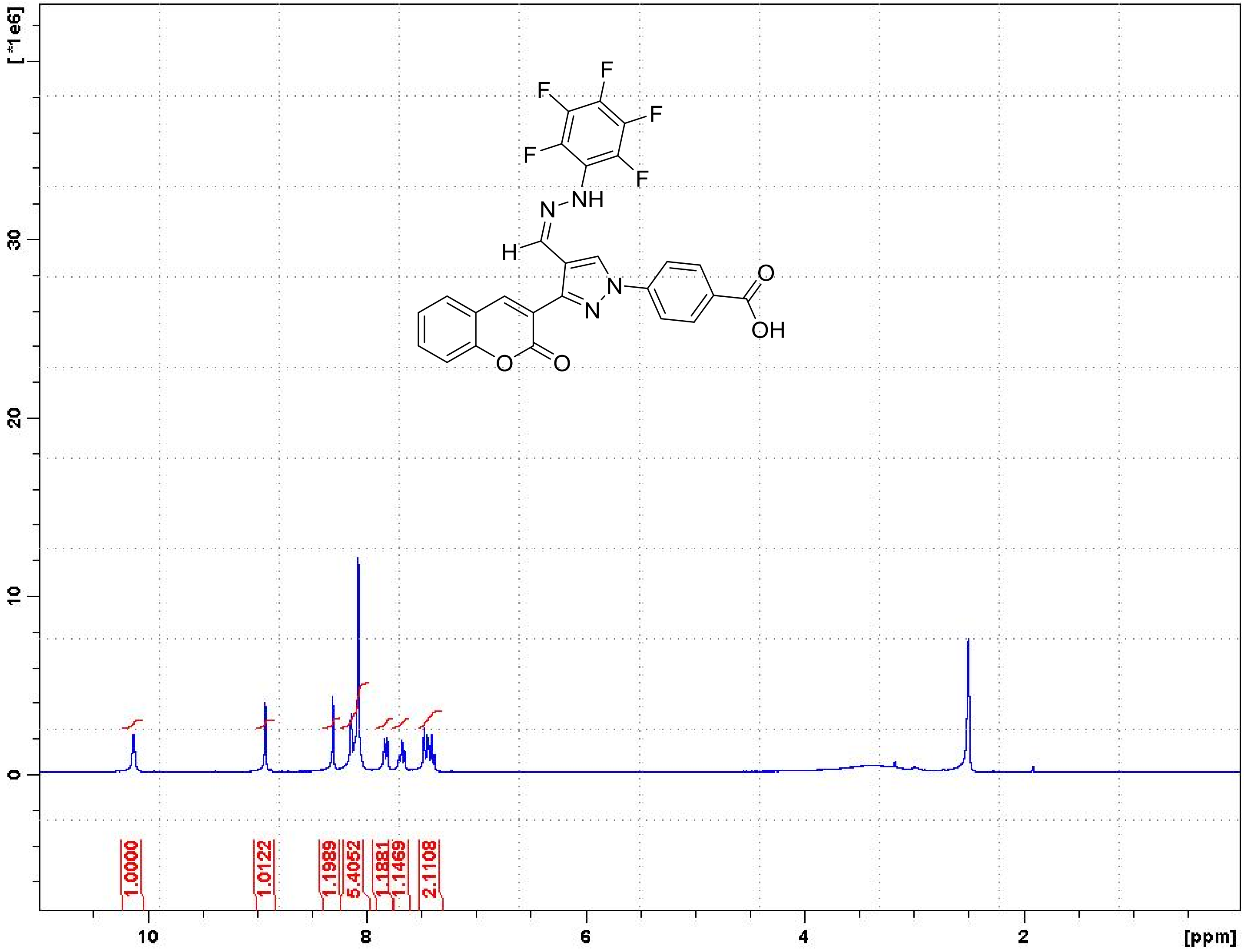

^{13}C NMR spectrum of compound **20**

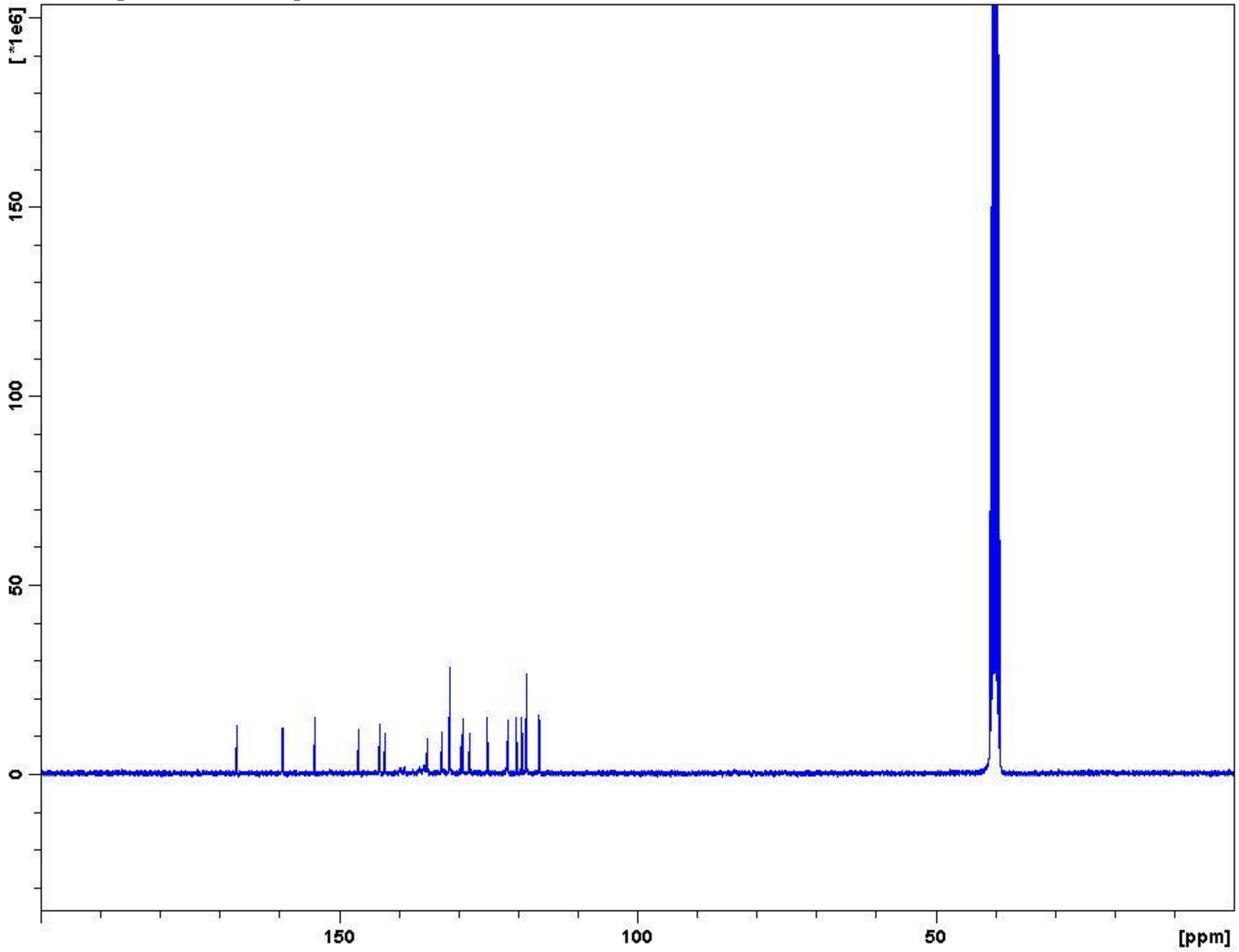

4-[4-[(E)-[(2,4-dichlorophenyl)hydrazono]methyl]-3-(2-oxochromen-3-yl)pyrazol-1-yl]benzoic acid (21)

^{1}H NMR spectrum

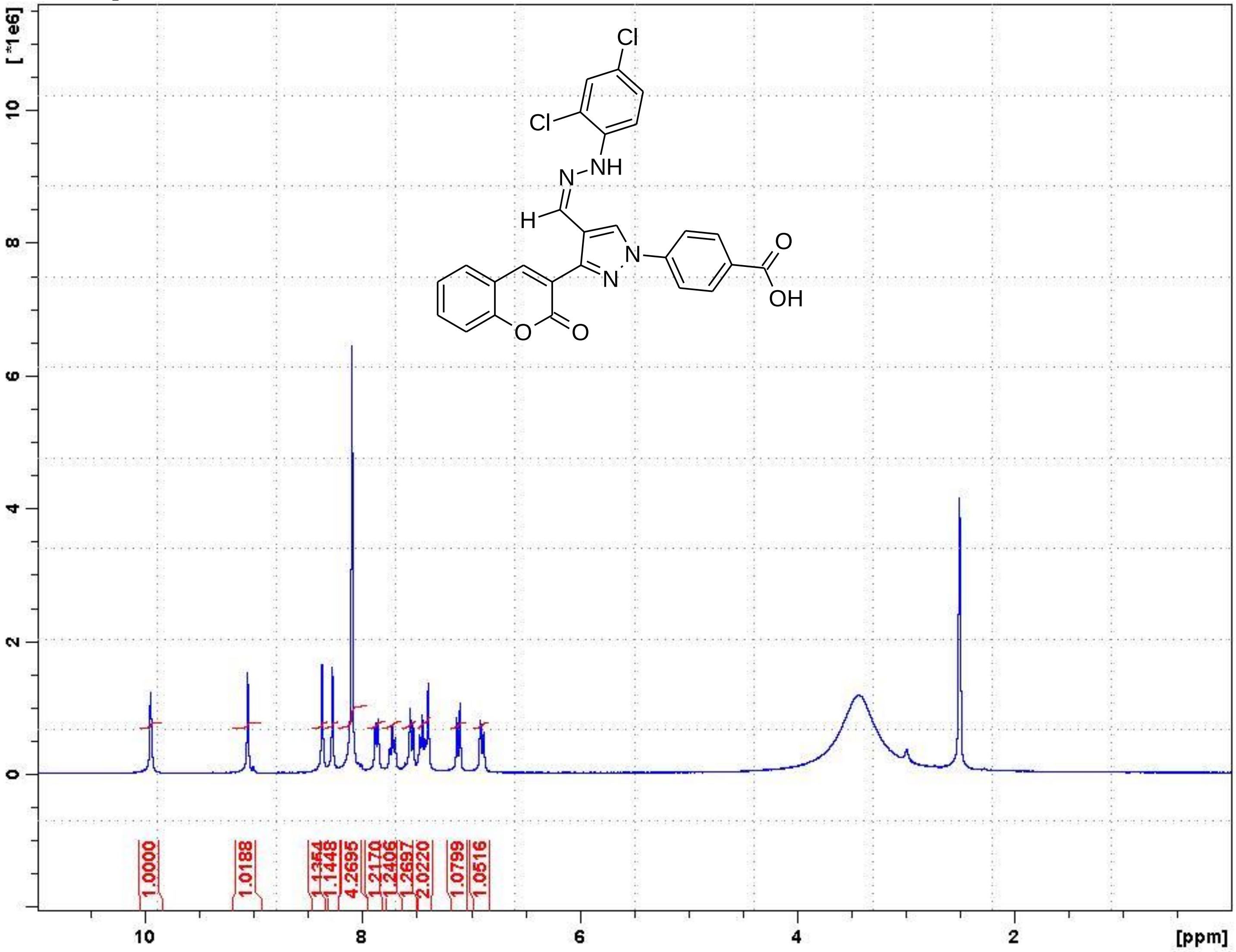

^{13}C NMR spectrum of compound **21**

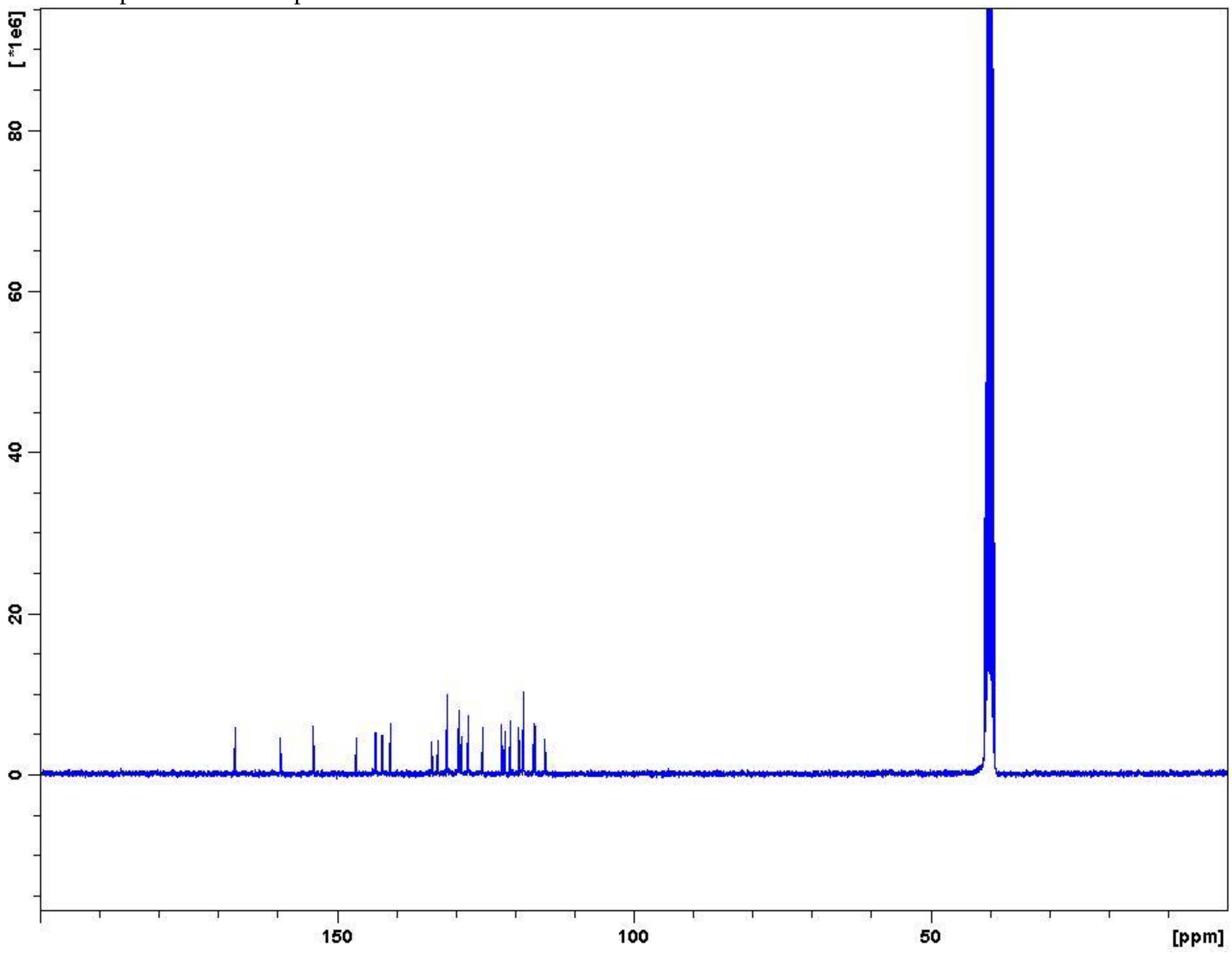

4-[4-[(E)-[(3-chloro-2-fluoro-phenyl)hydrazono]methyl]-3-(2-oxochromen-3-yl)pyrazol-1-yl]benzoic acid (22)

^{1}H NMR spectrum

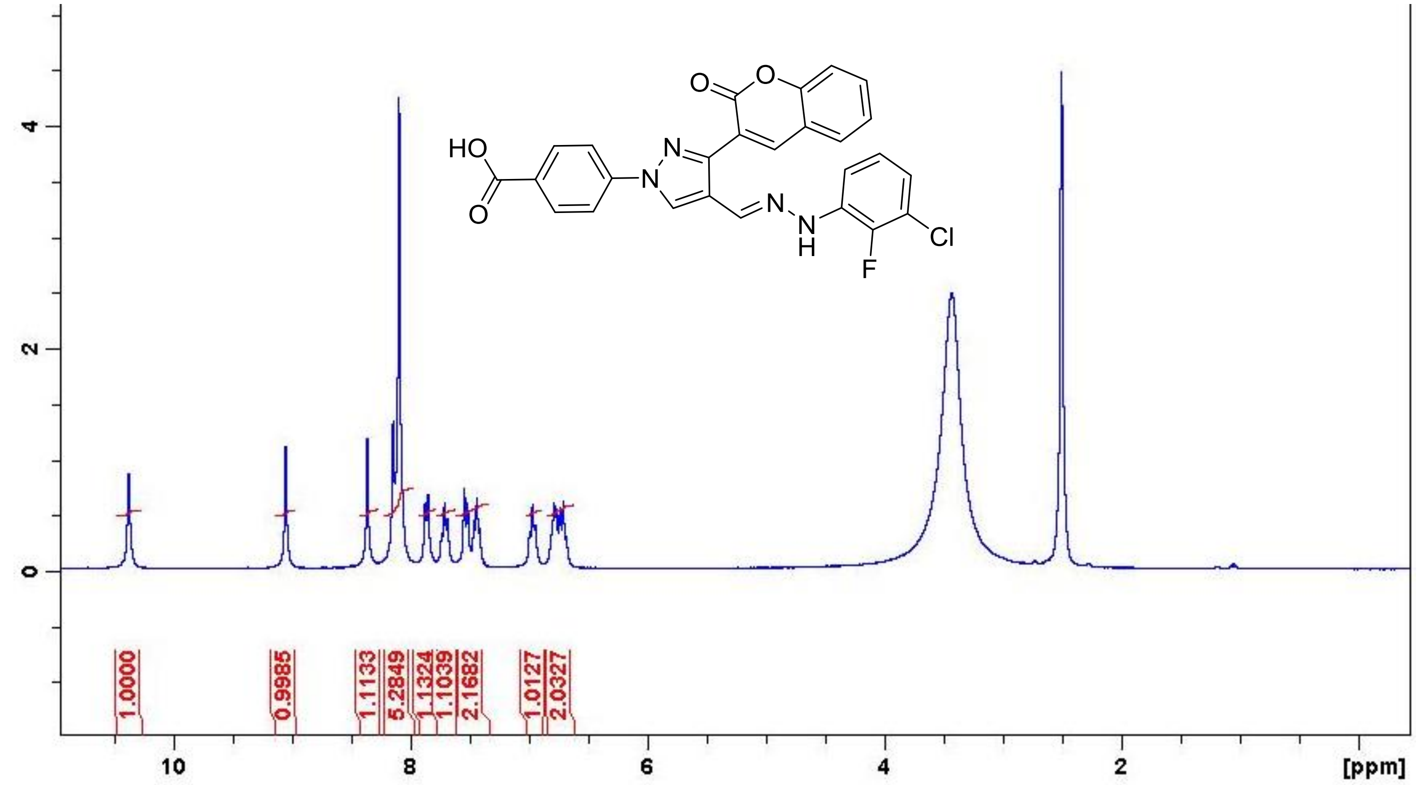

^{13}C NMR spectrum of compound **22**

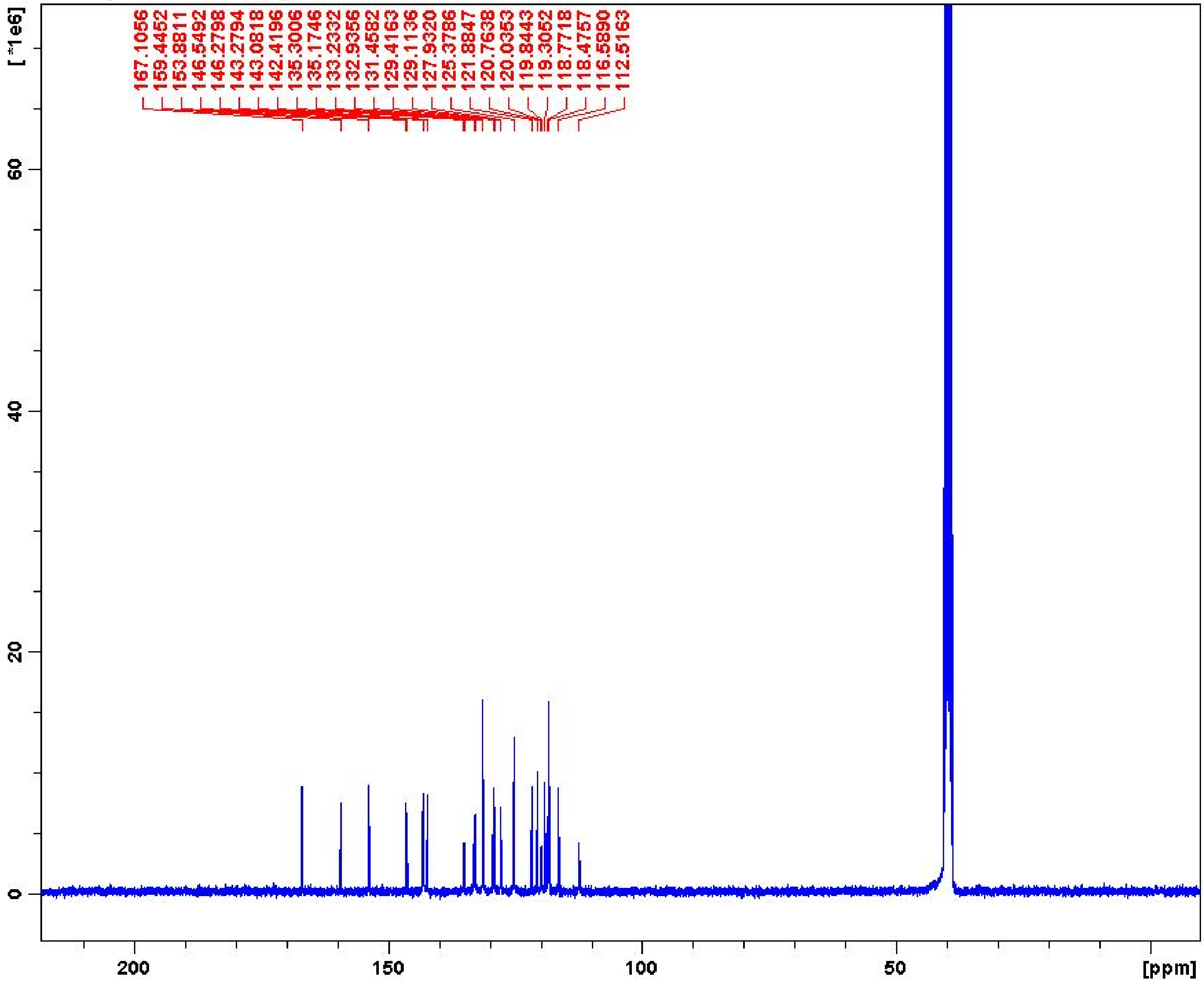

4-[4-[(E)-[(3-chloro-4-fluoro-phenyl)hydrazono]methyl]-3-(2-oxochromen-3-yl)pyrazol-1-yl]benzoic acid (23)

^{1}H NMR spectrum

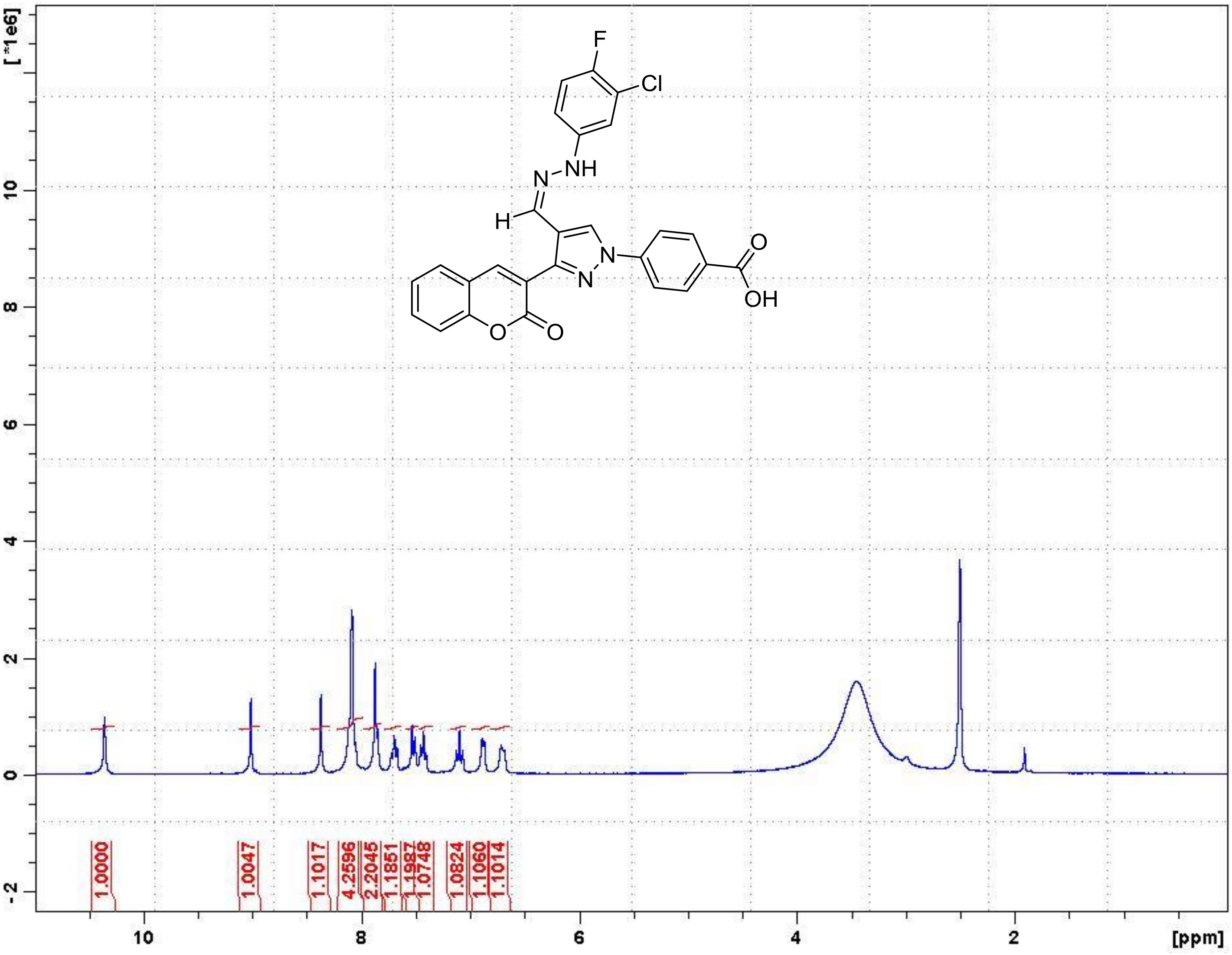

^{13}C NMR spectrum of compound **23**

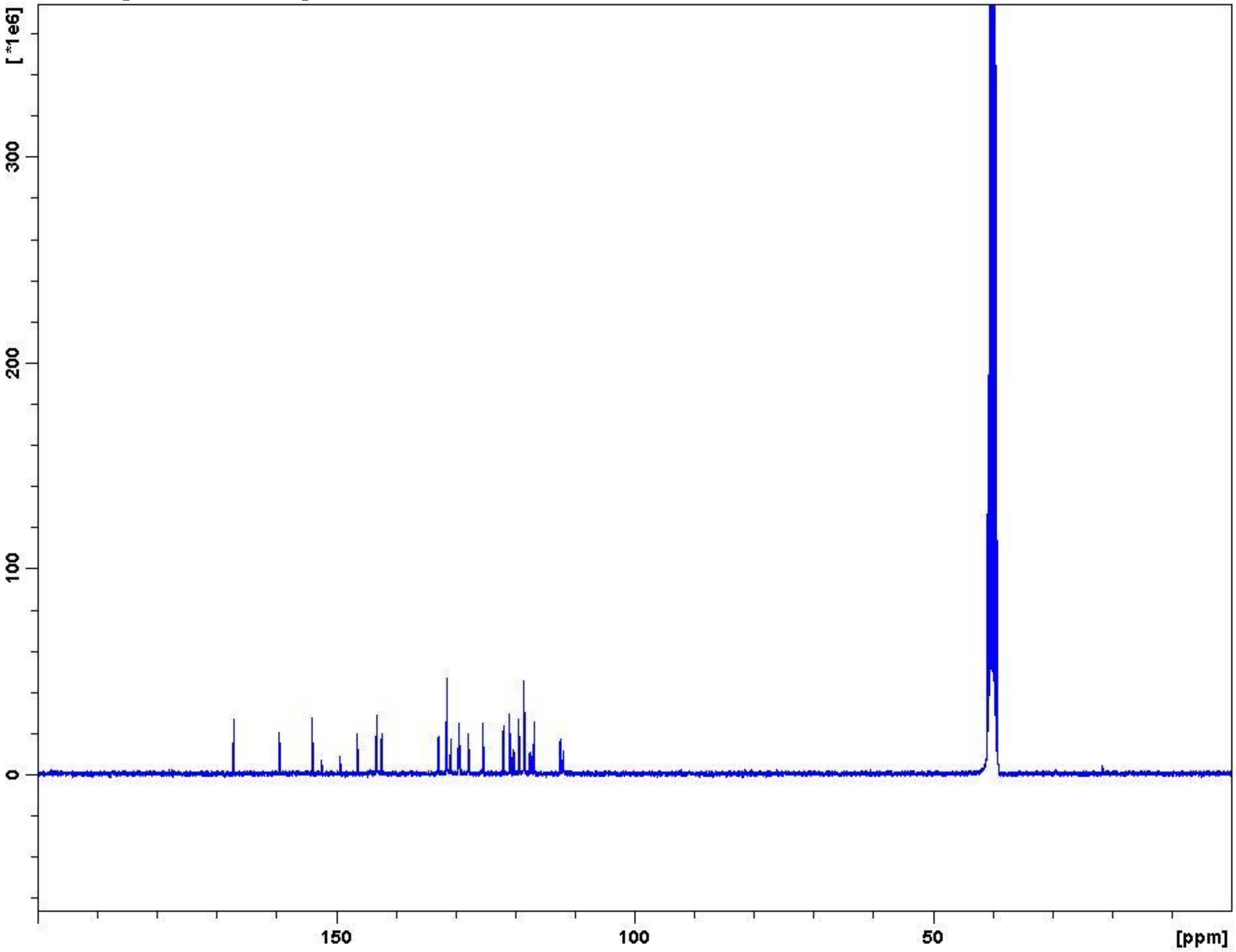

4-[3-(2-oxochromen-3-yl)-4-[(E)-[[4-(trifluoromethyl)phenyl]hydrazono]methyl]pyrazol-1-yl]benzoic acid (24)

^{1}H NMR spectrum

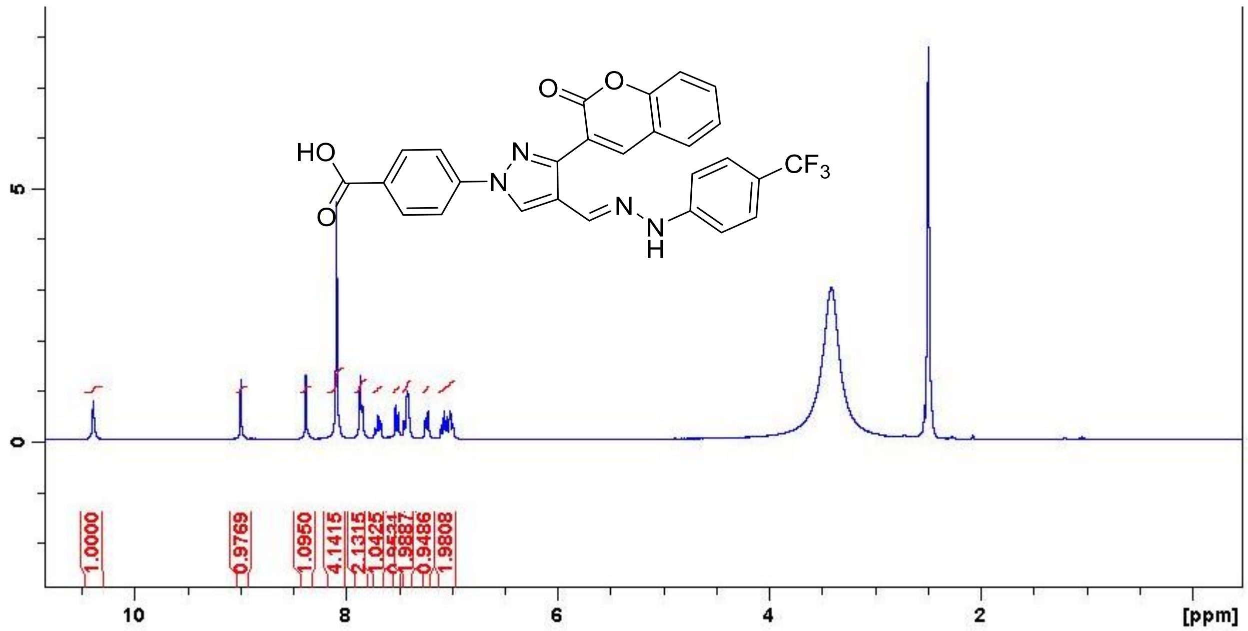

^{13}C NMR spectrum of compound **24**

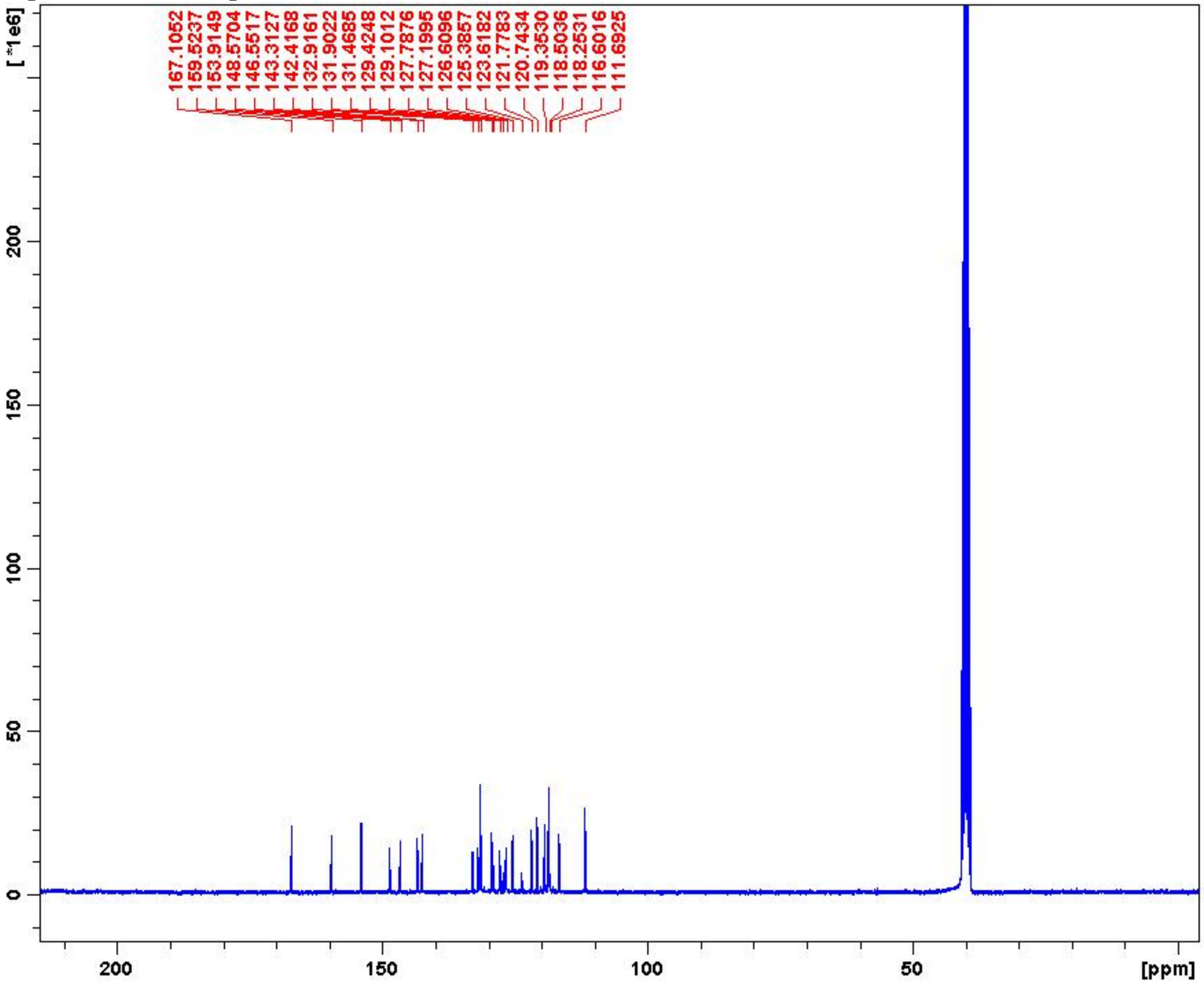

4-[4-[(E)-[(4-nitrophenyl)hydrazono]methyl]-3-(2-oxochromen-3-yl)pyrazol-1-yl]benzoic acid (25)

^{1}H NMR spectrum

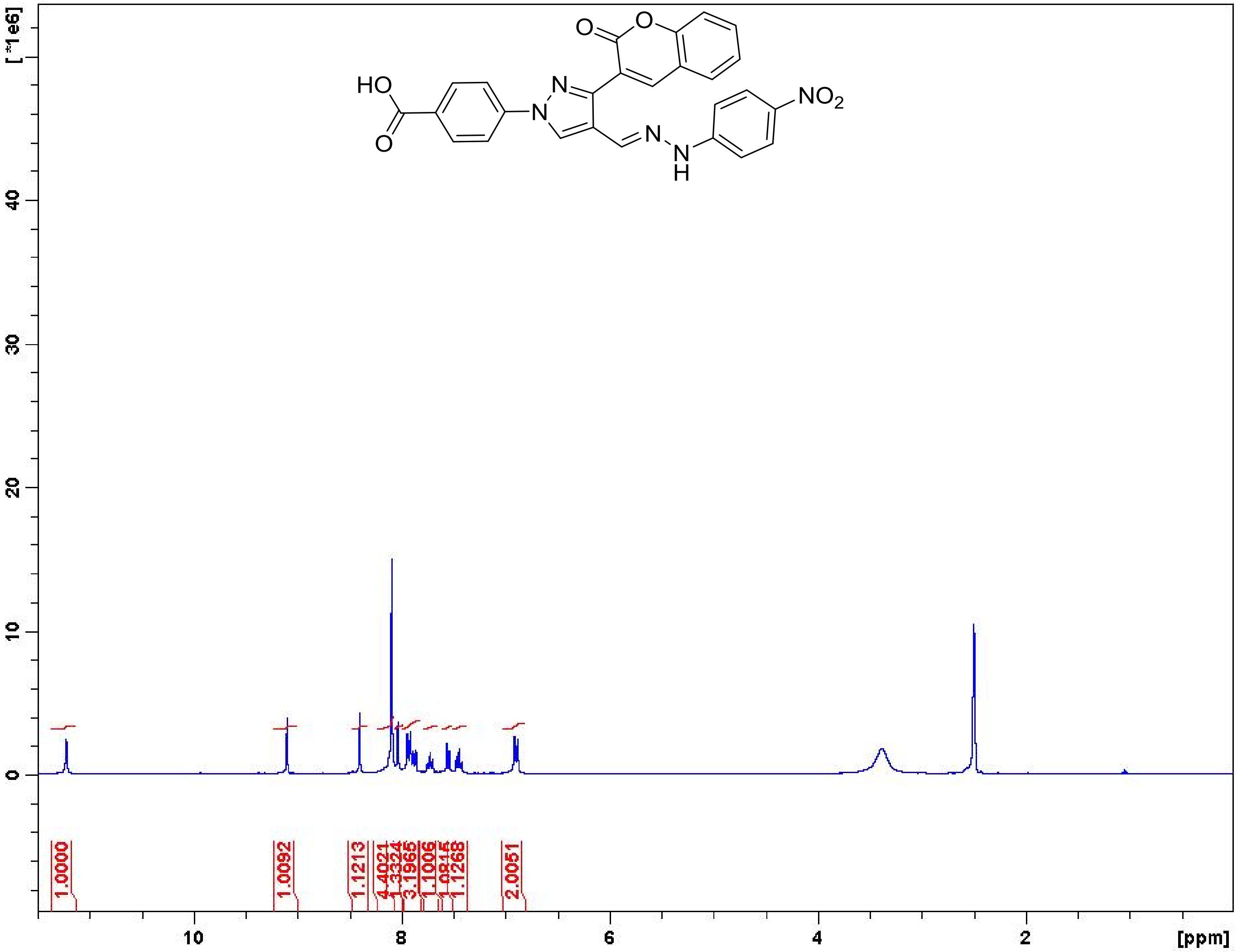

^{13}C NMR spectrum of compound **25**

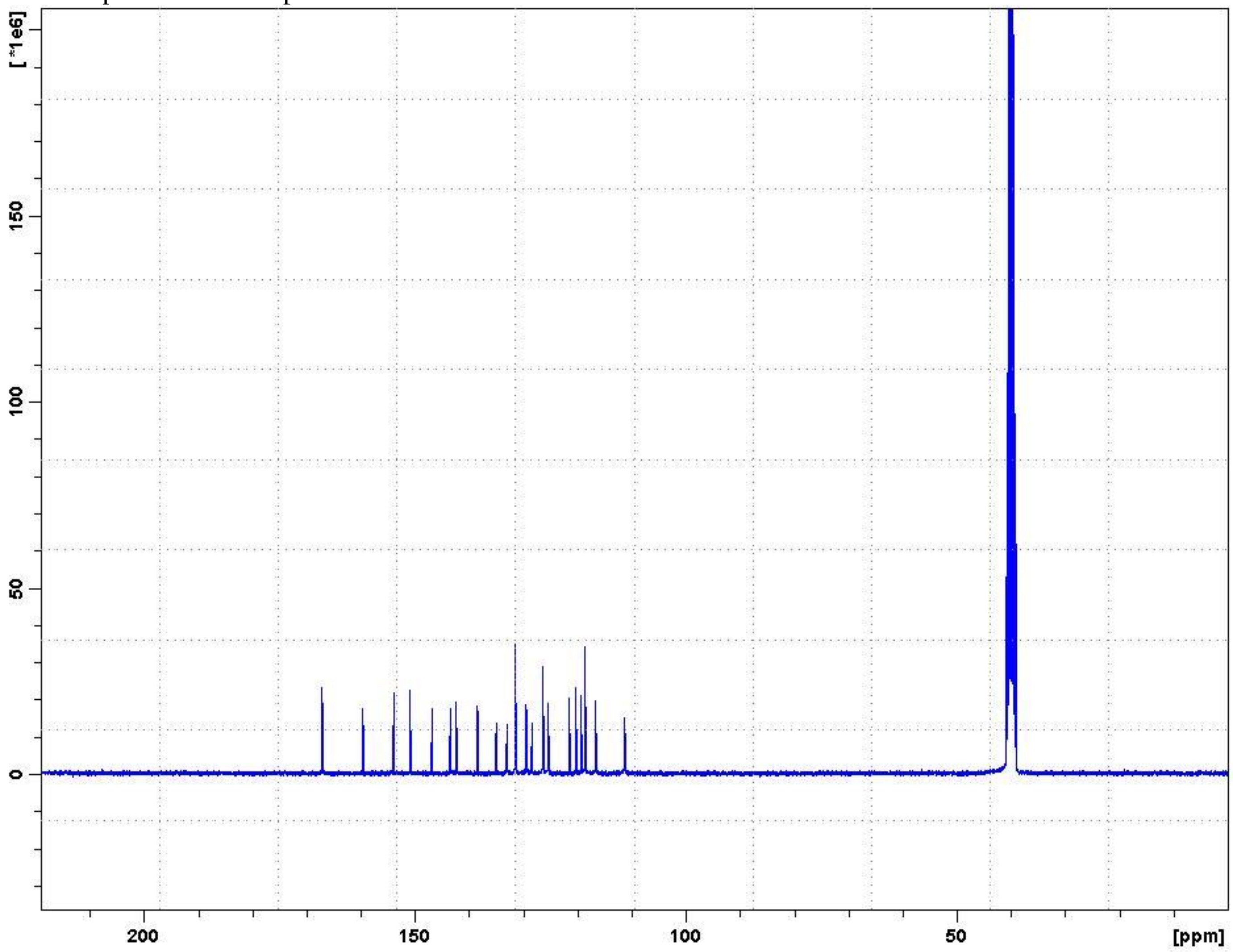

4-[(2E)-2-[[1-(4-carboxyphenyl)-3-(2-oxochromen-3-yl)pyrazol-4-yl]methylene]hydrazino]benzoic acid (26)

^{1}H NMR spectrum

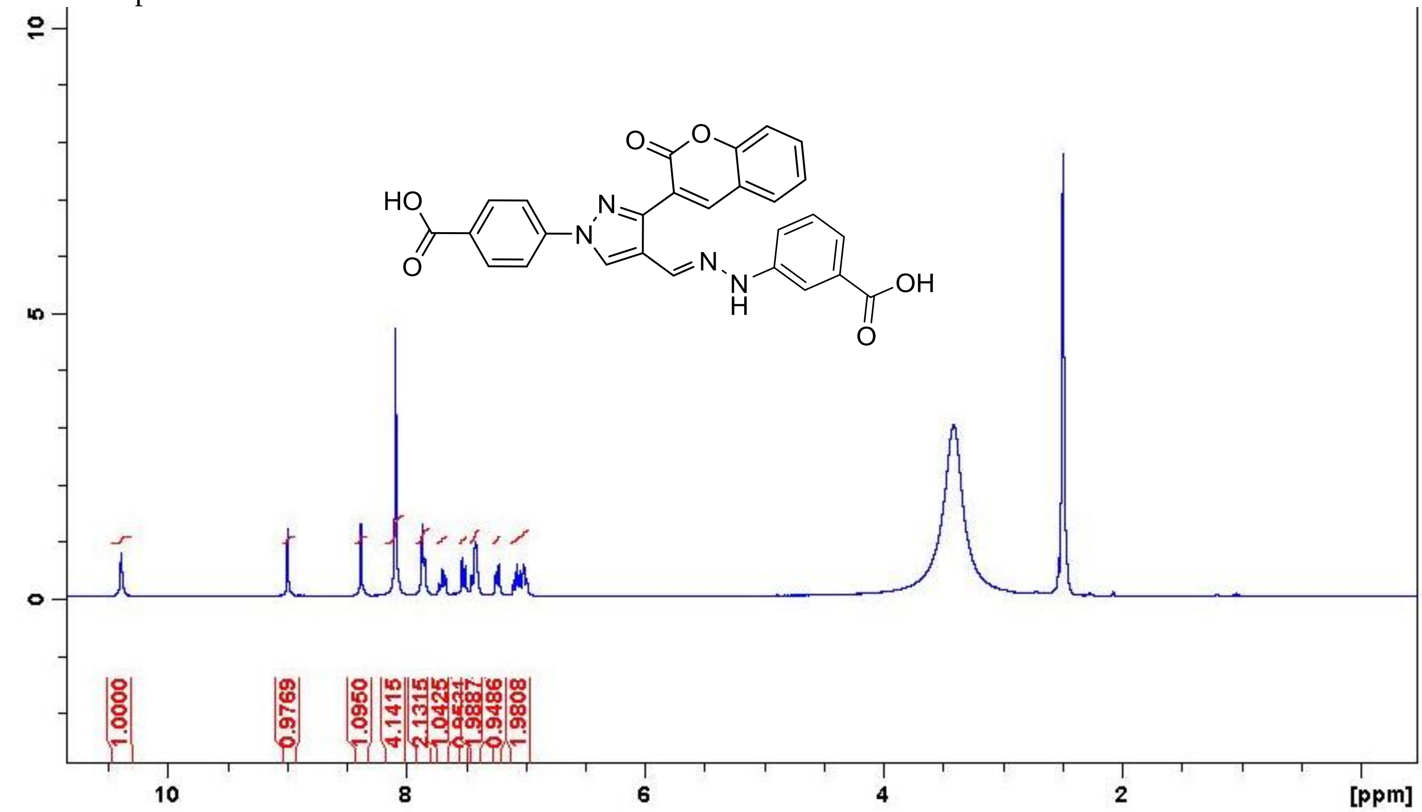

^{13}C NMR spectrum of compound **26**

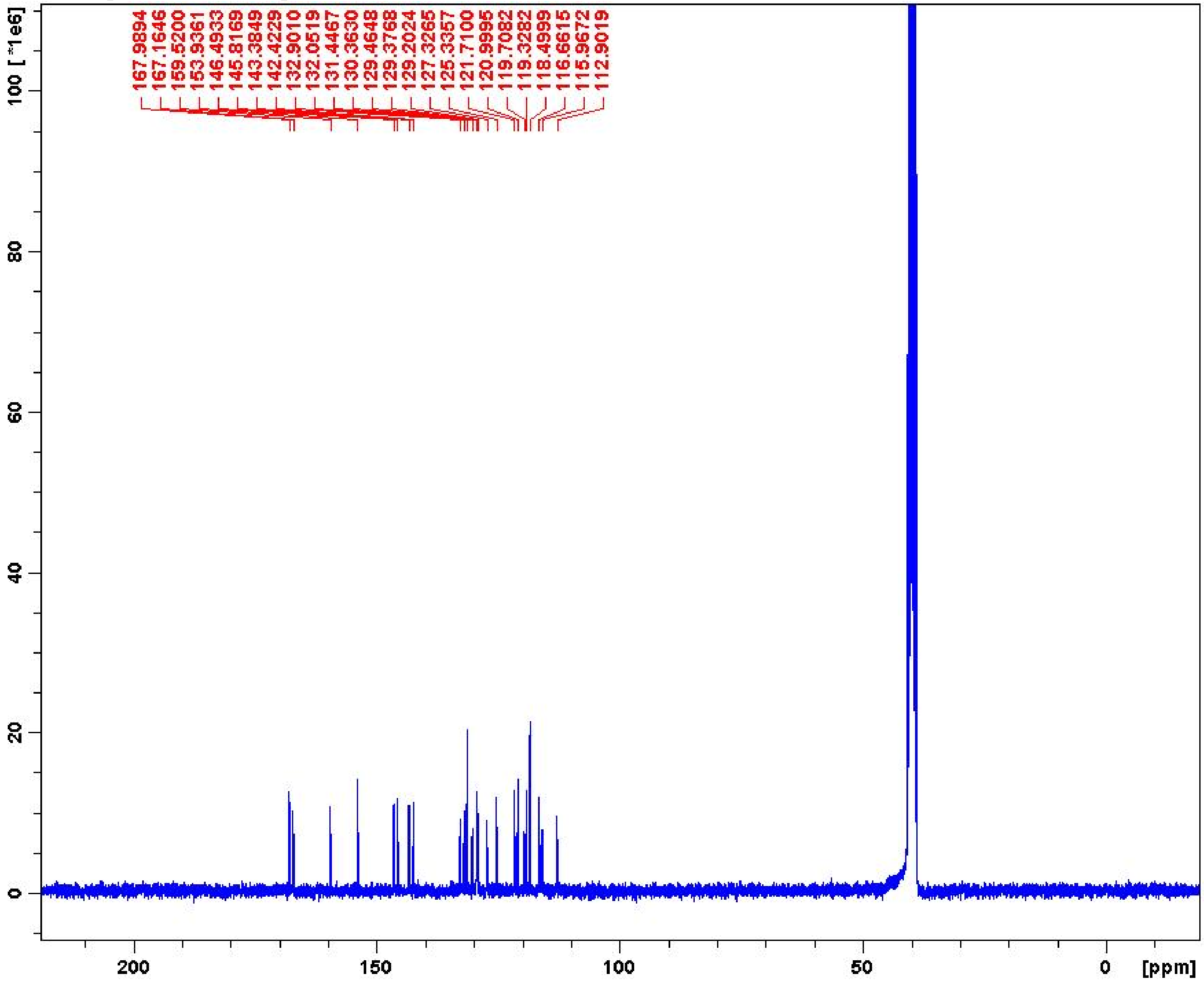

4-[(2E)-2-[[1-(4-carboxyphenyl)-3-(2-oxochromen-3-yl)pyrazol-4-yl]methylene]hydrazino]benzoic acid (27)

^{1}H NMR spectrum

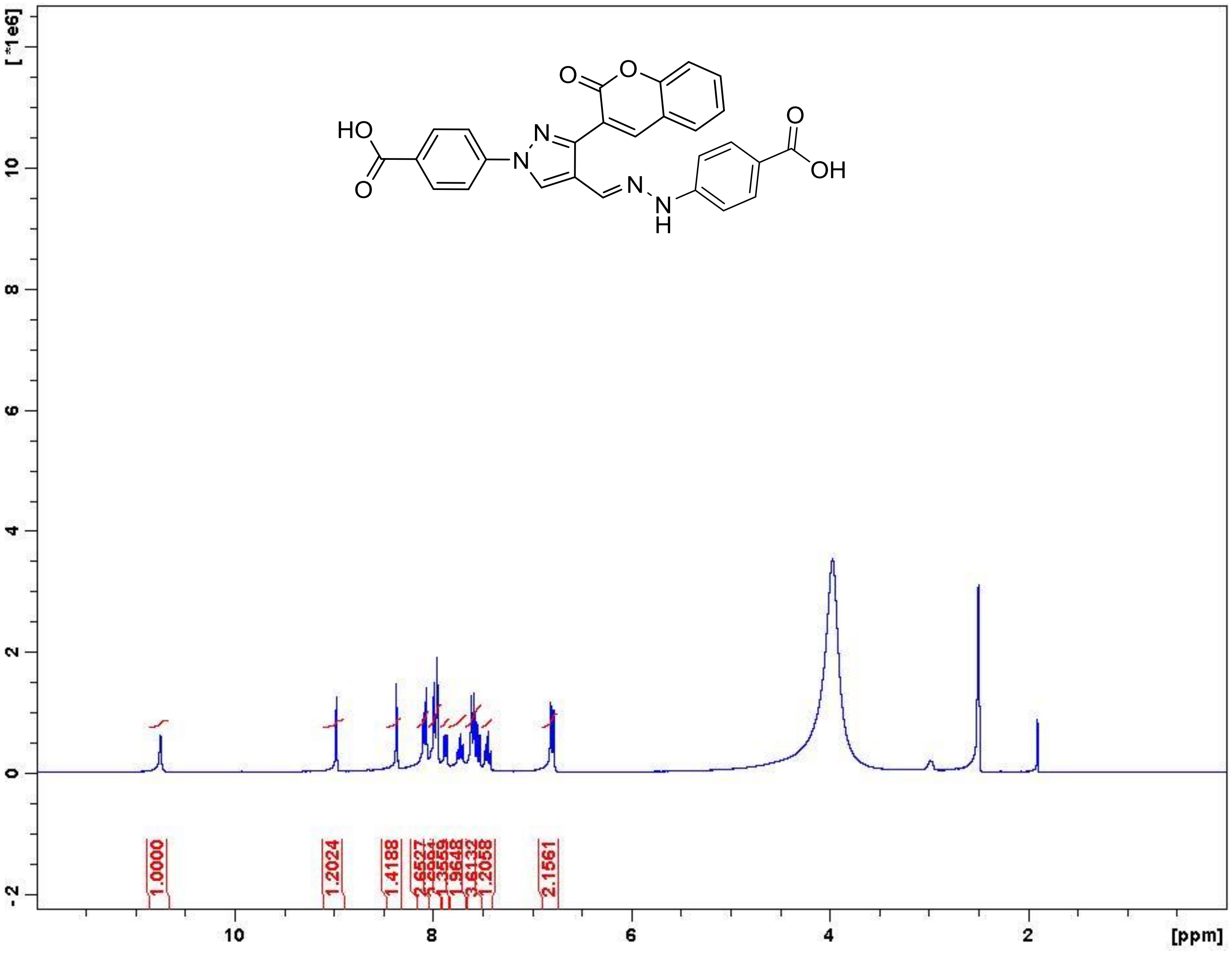

^{13}C NMR spectrum of compound **27**

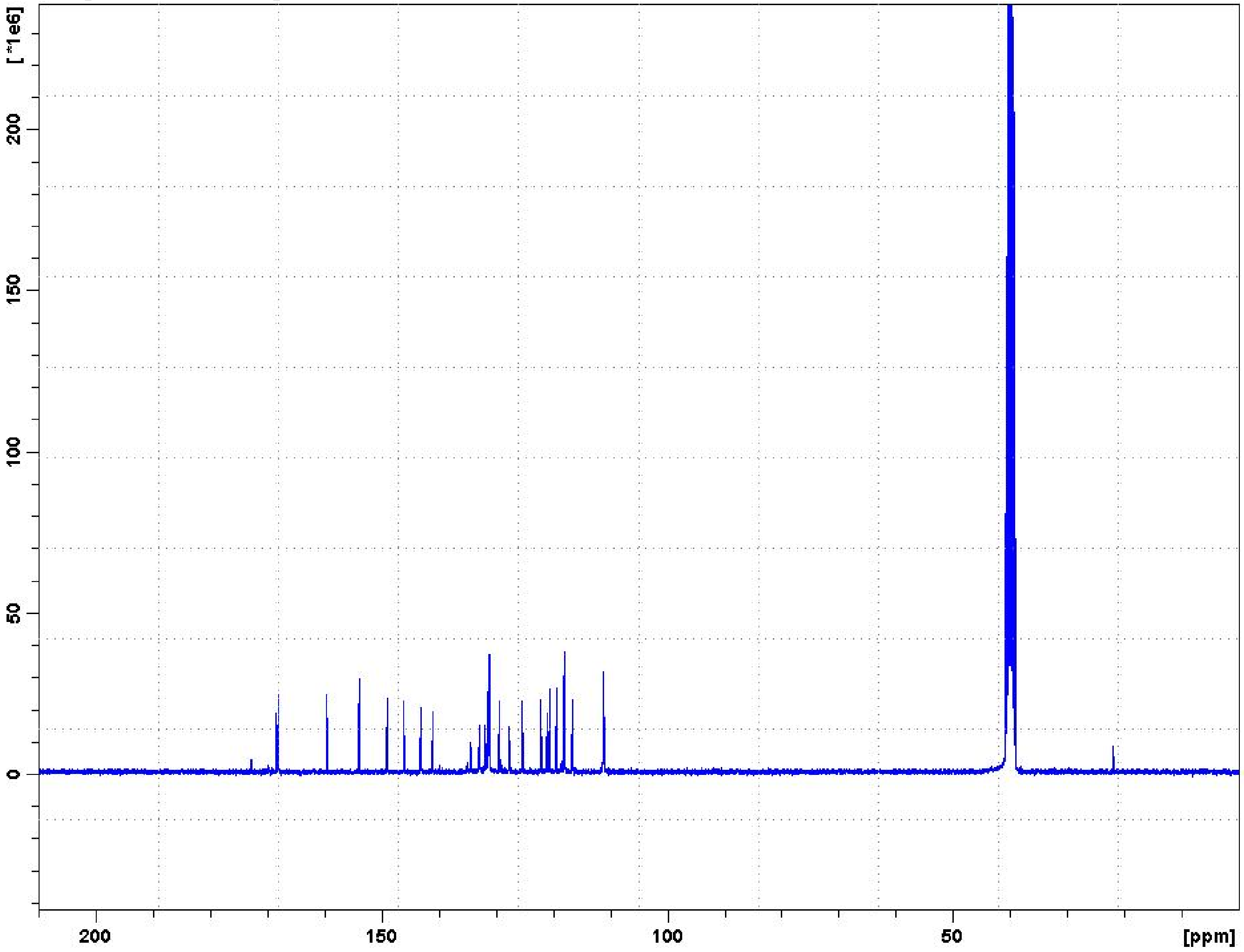

.

4-[(2E)-2-[[1-(4-cyanophenyl)-3-(2-oxochromen-3-yl)pyrazol-4-yl]methylene]hydrazino]benzoic acid (28)

^{1}H NMR spectrim

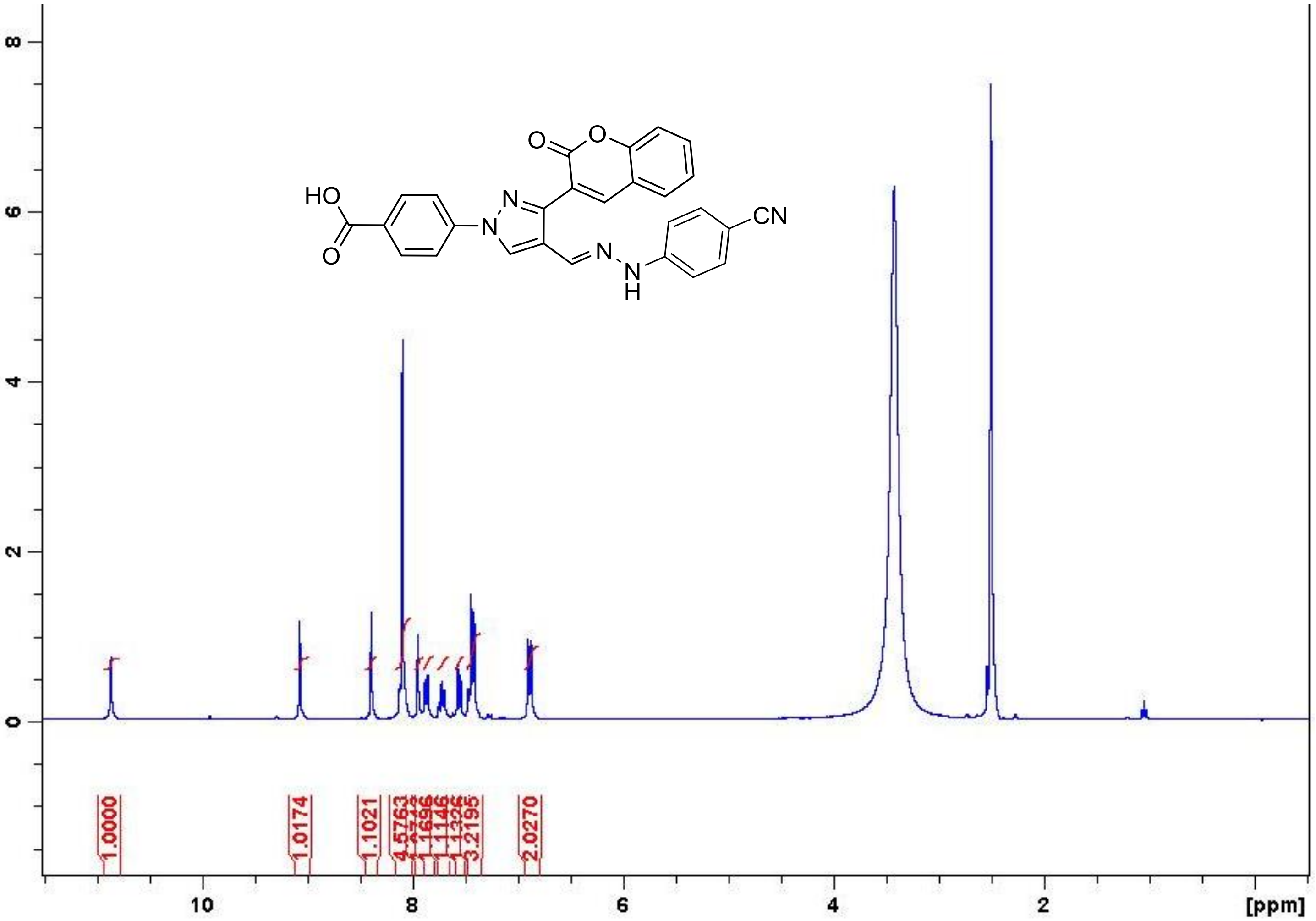

^{13}C NMR spectrum of compound **28**

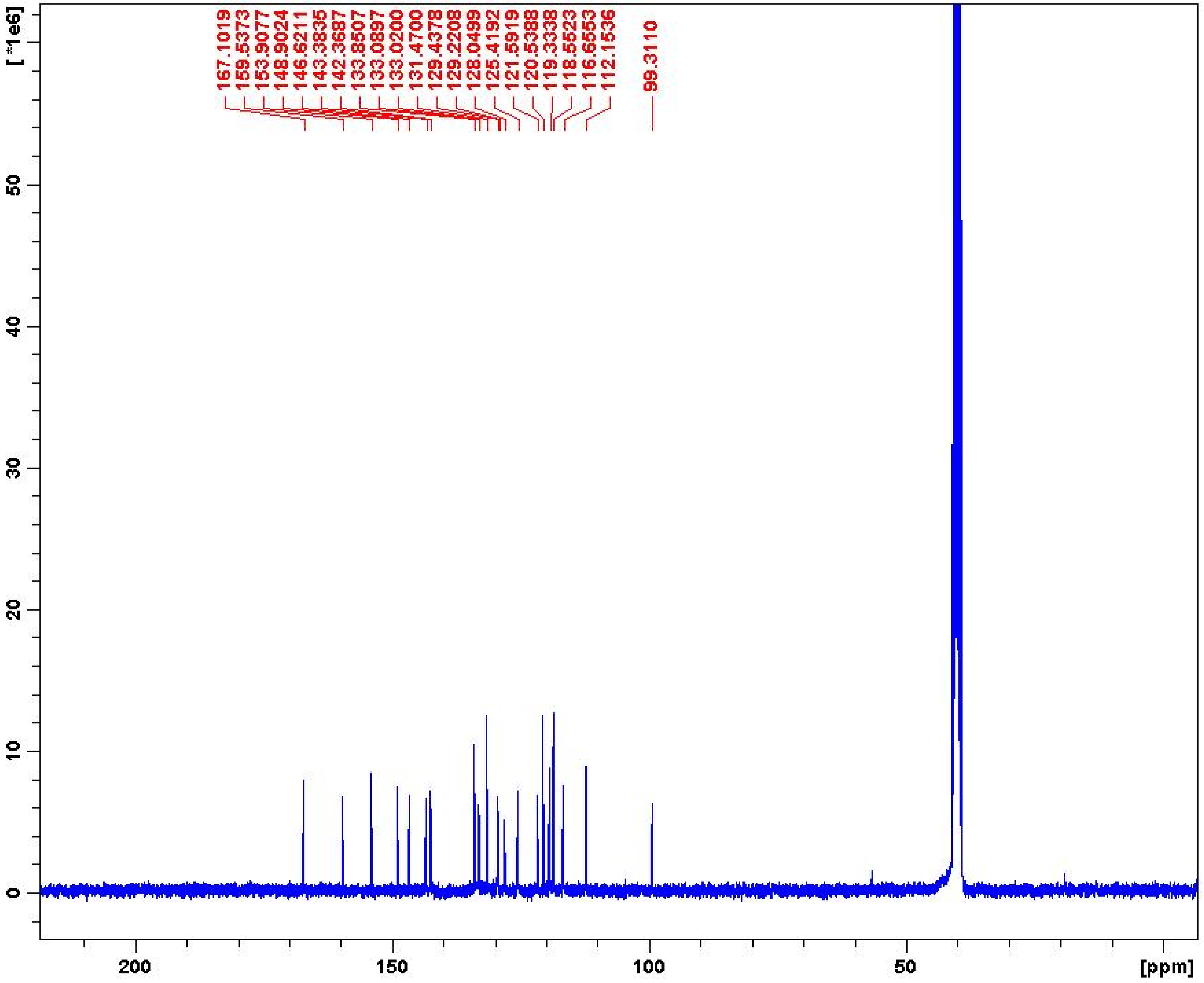
167.1019
159.5373
153.9077
148.9024
146.6211
143.3835
142.3687
133.8507
133.0897
133.0200
131.4700
129.4378
129.2208
128.0499
125.4192
121.5919
120.5388
119.3338
118.5523
116.6553
112.1536
99.3110
[*1e6]
50
40
30
20
10
0
200
150
100
50
[ppm]

4-[4-[(E)-(methylhydrazono)methyl]-3-(2-oxochromen-3-yl)pyrazol-1-yl]benzoic acid (29)

^{1}H NMR spectrum

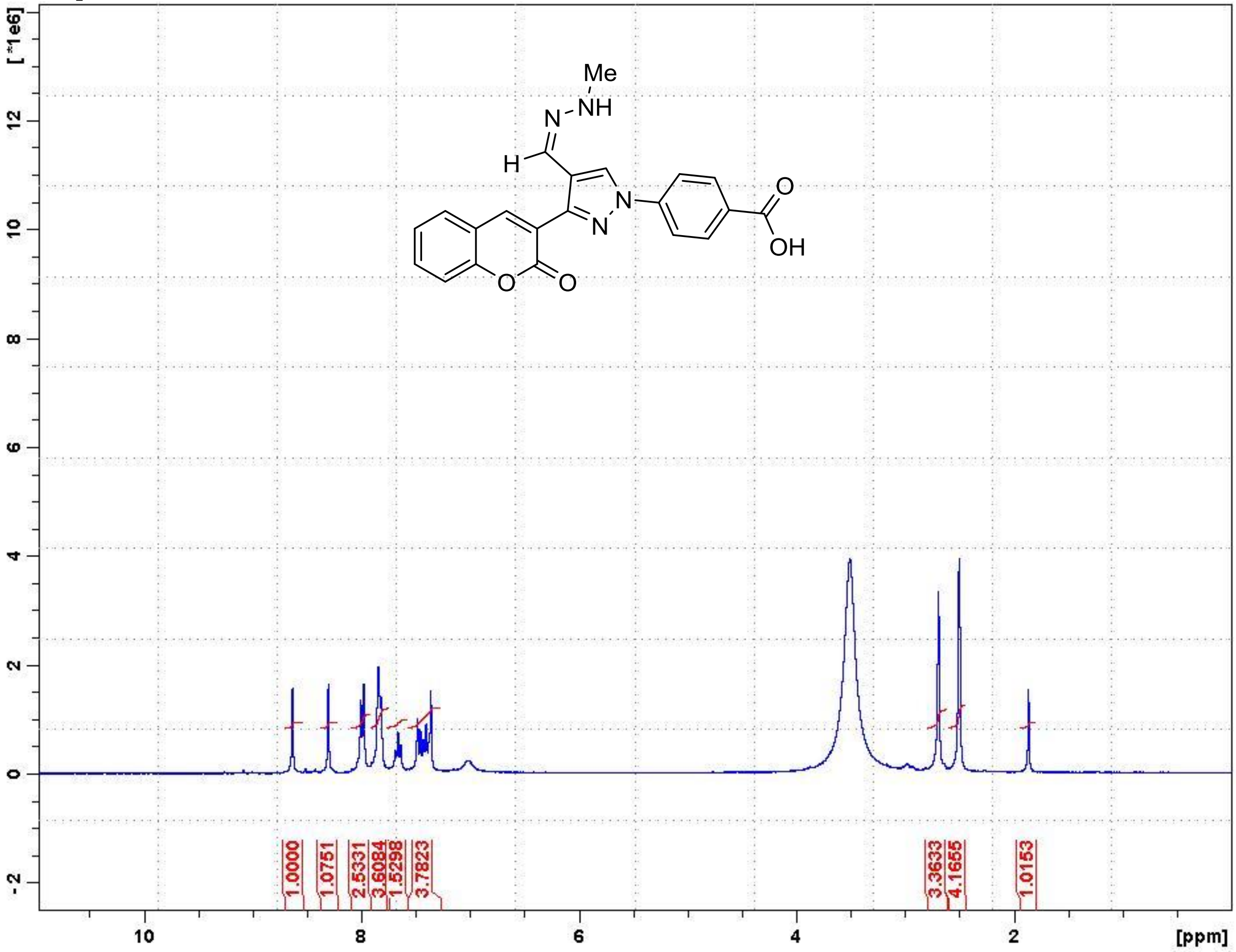

^{13}C NMR spectrum of compound **29**

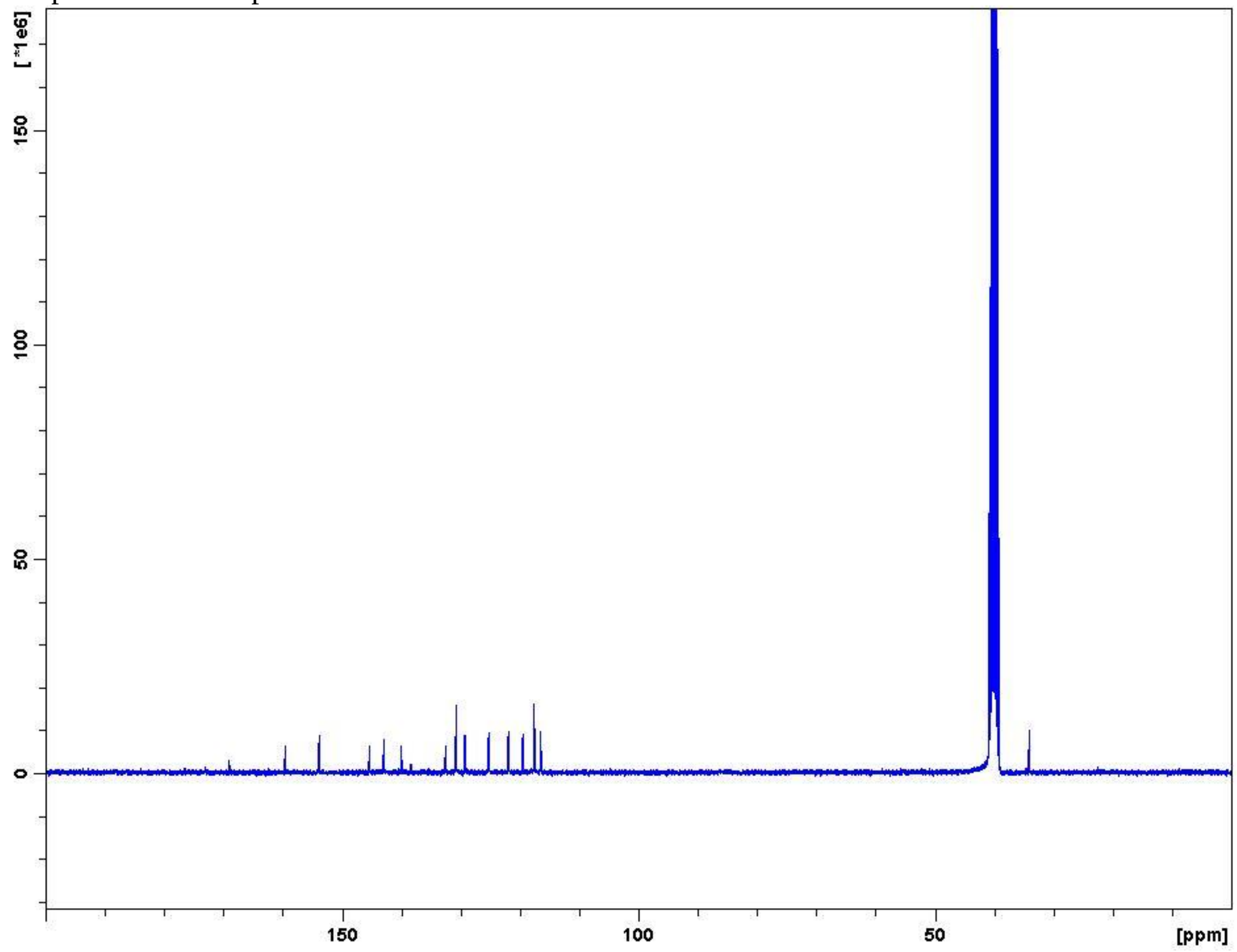

4-[3-(2-oxochromen-3-yl)-4-[(E)-1-piperidyliminomethyl]pyrazol-1-yl]benzoic acid (30)

^{1}H NMR spectrum

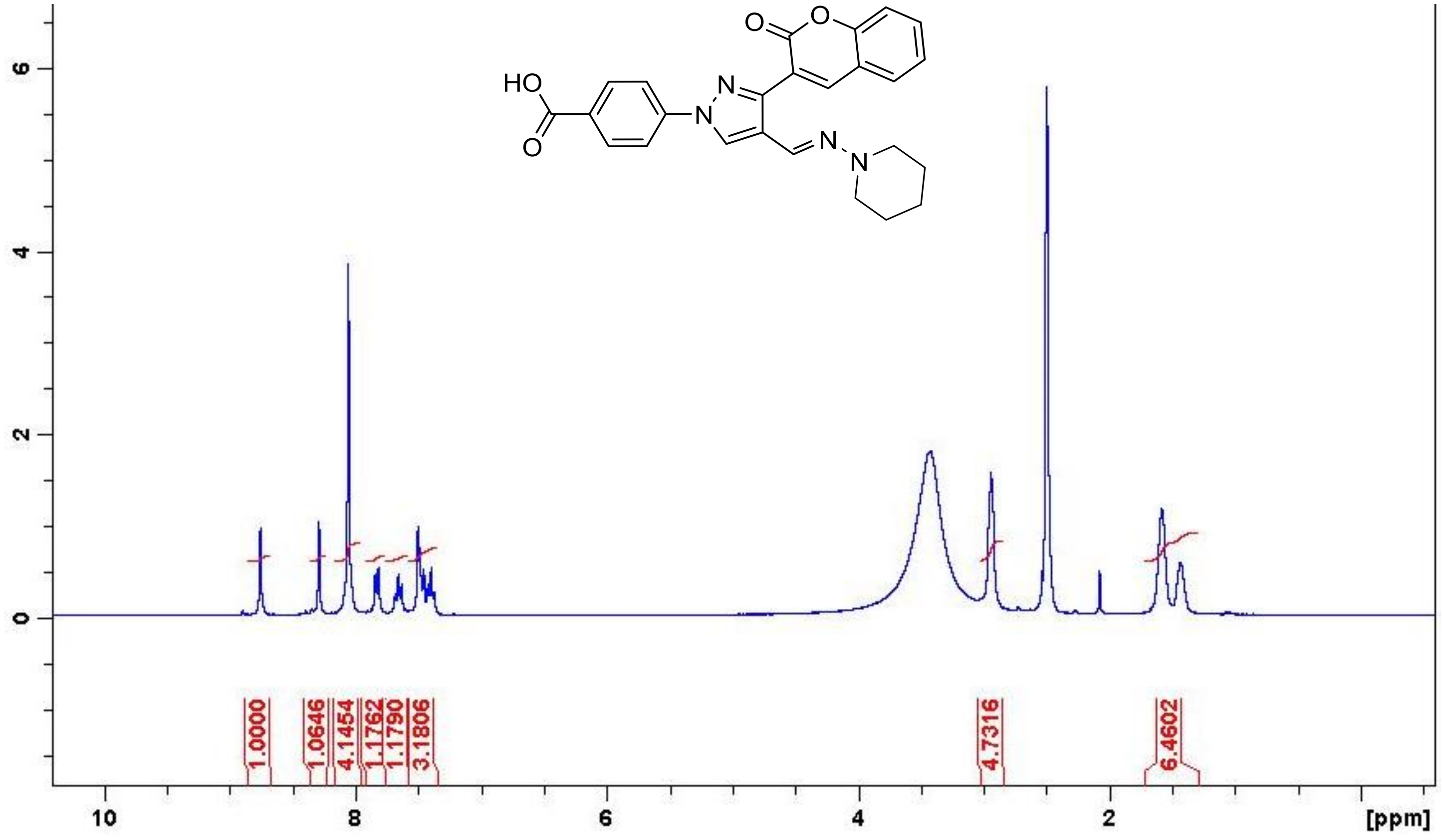

^{13}C NMR spectrum of compound **30**

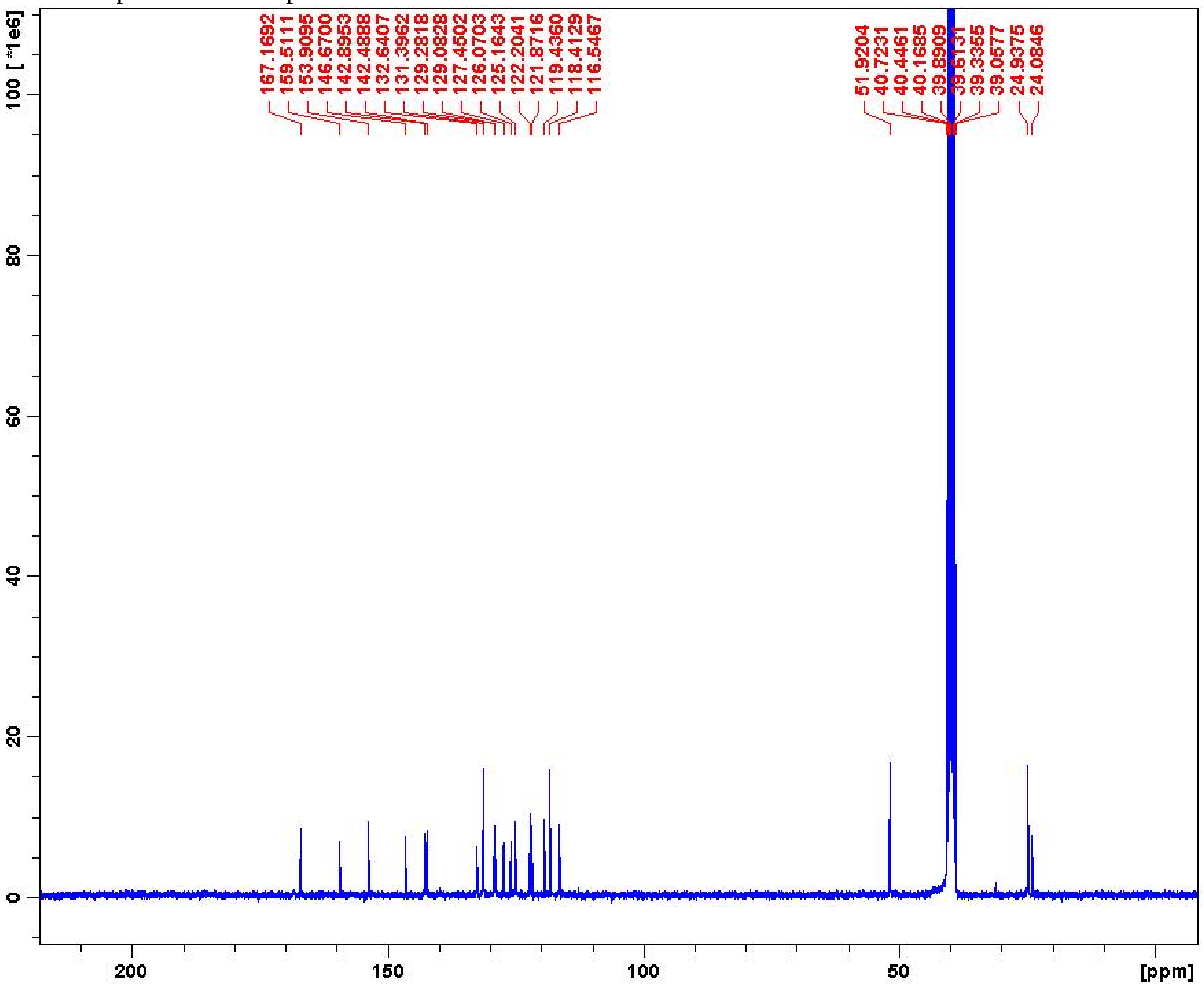

4-[4-[(E)-(benzylhydrazono)methyl]-3-(2-oxochromen-3-yl)pyrazol-1-yl]benzoic acid (31)

^{1}H NMR spectrum

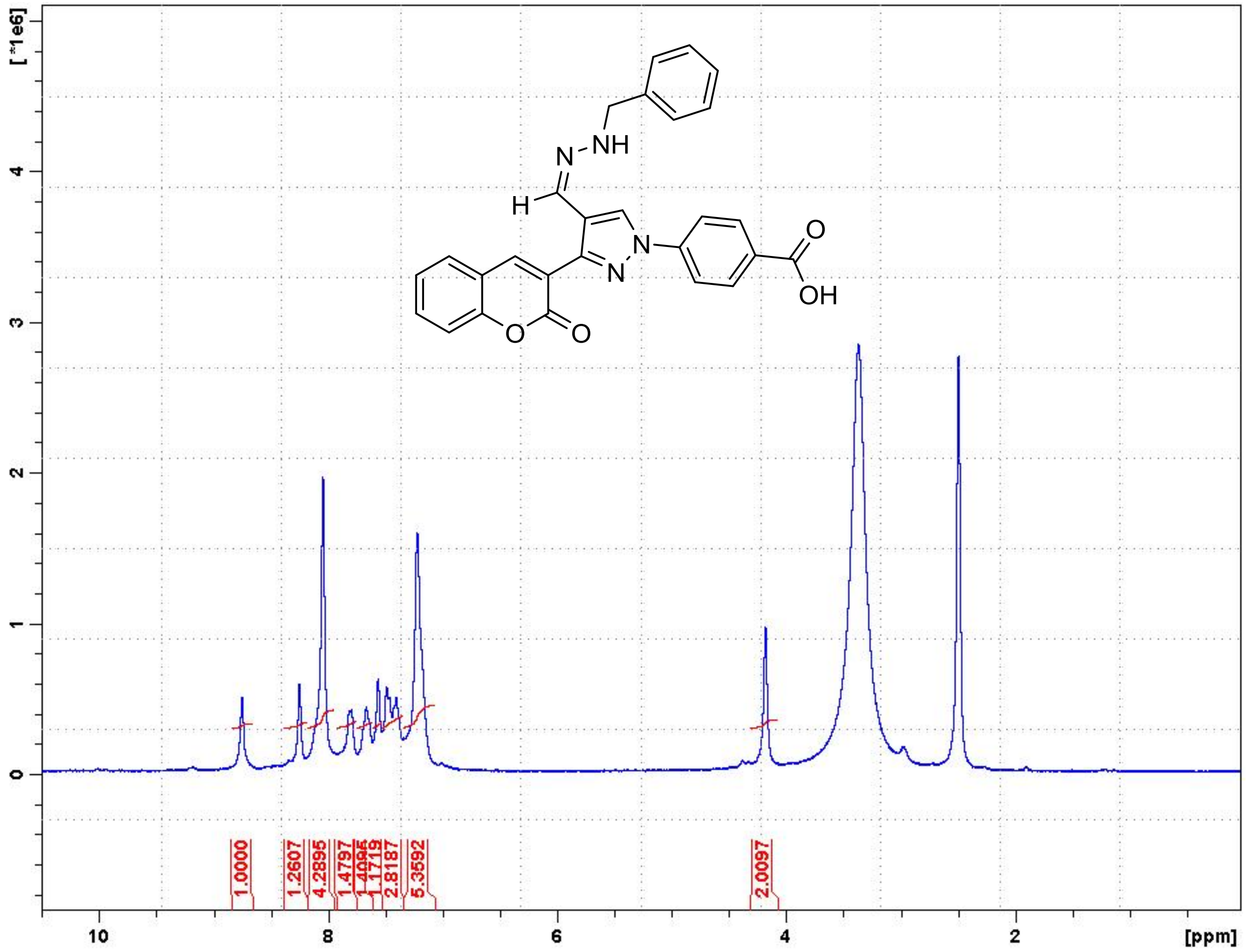

^{13}C NMR spectrum of compound **31**

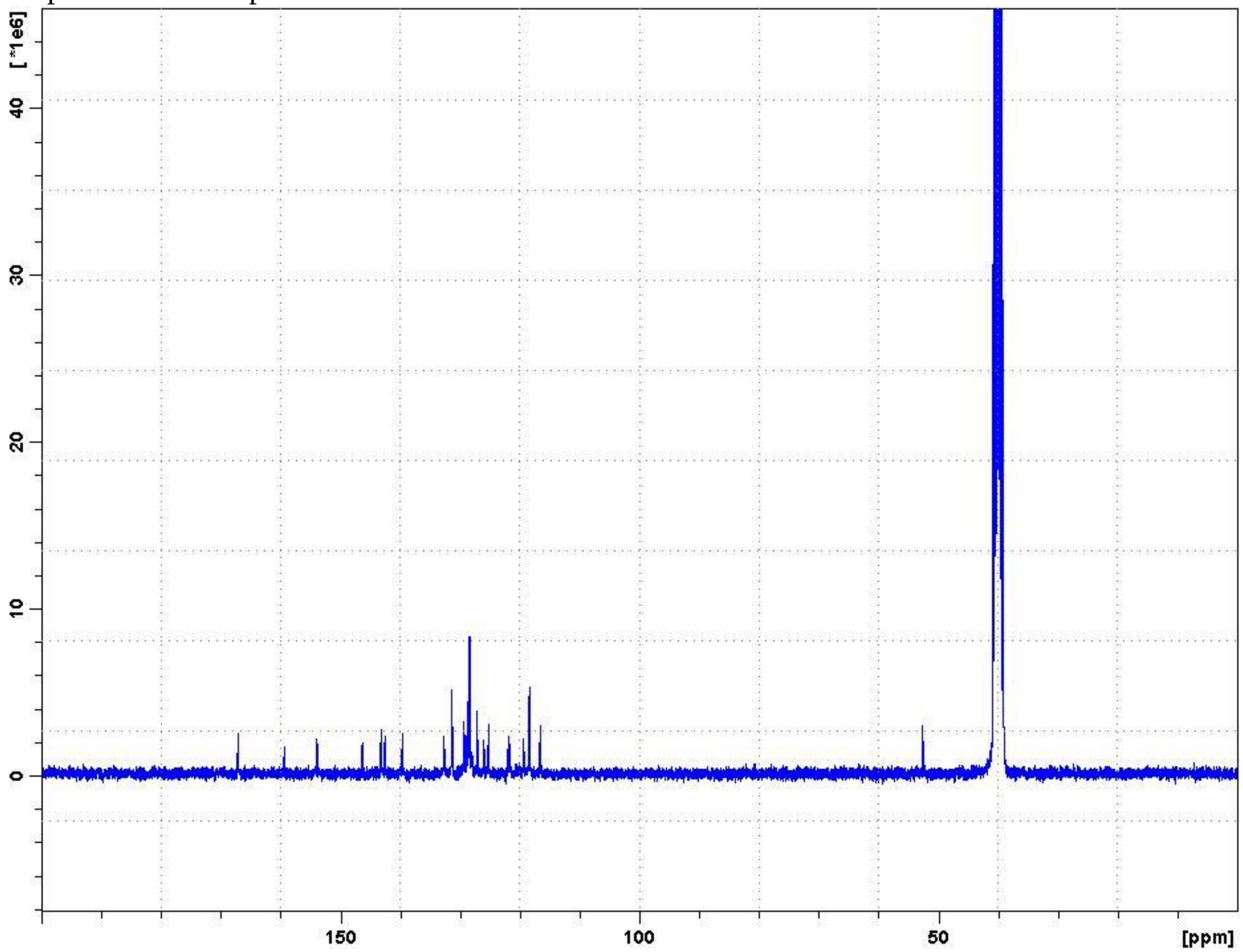

References:

1. Brider, J.; Rowe, T.; Gibler, D. J.; Gottsponer, A.; Delancey, E.; Branscum, M. D.; Ontko, A.; Gilmore, D.; Alam, M. A., Synthesis and antimicrobial studies of azomethine and N-arylamine derivatives of 4-(4-formyl-3-phenyl-1H-pyrazol-1-yl)benzoic acid as potent anti-methicillin-resistant Staphylococcus aureus agents. *Med. Chem. Res.* **2016,** *25* (11), 2691-2697.
2. Allison, D.; Delancey, E.; Ramey, H.; Williams, C.; Alsharif, Z. A.; Al-khattabi, H.; Ontko, A.; Gilmore, D.; Alam, M. A., Synthesis and antimicrobial studies of novel derivatives of 4-(4-formyl-3-phenyl-1H-pyrazol-1-yl)benzoic acid as potent anti-Acinetobacter baumannii agents. *Bioorg. Med. Chem. Lett.* **2017,** *27* (3), 387-392.
3. Zakeyah, A. A.; Whitt, J.; Duke, C.; Gilmore, D. F.; Meeker, D. G.; Smeltzer, M. S.; Alam, M. A., Synthesis and antimicrobial studies of hydrazone derivatives of 4-[3-(2,4-difluorophenyl)-4-formyl-1H-pyrazol-1-yl]benzoic acid and 4-[3-(3,4-difluorophenyl)-4-formyl-1H-pyrazol-1-yl]benzoic acid. *Bioorg. Med. Chem. Lett.* **2018,** *28* (17), 2914-2919.
4. Sarker, S. D.; Nahar, L.; Kumarasamy, Y., Microtitre plate-based antibacterial assay incorporating resazurin as an indicator of cell growth, and its application in the in vitro antibacterial screening of phytochemicals. *Methods (San Diego, Calif.)* **2007,** *42* (4), 321-324.
